# Switchable Oxidative Reactions of *N*-allyl-2-Aminophenols: Palladium-Catalyzed Alkoxyacyloxylation vs an Intramolecular Diels–Alder Reaction

**DOI:** 10.1021/acs.orglett.1c02539

**Published:** 2021-09-27

**Authors:** Sabrina Giofrè, Manfred Keller, Leonardo Lo Presti, Egle M. Beccalli, Letizia Molteni

**Affiliations:** † DISFARM, Sezione di Chimica Generale e Organica “A. Marchesini”, Università degli Studi di Milano, Via Venezian 21, 20133 Milano, Italy; ‡ Institut für Organische Chemie, Albert-Ludwigs-Universität Freiburg, Albertstrasse 21, 79104 Freiburg im Breisgau, Germany; § Dipartimento di Chimica, Università degli Studi di Milano, Via Golgi 19, 20133 Milano, Italy

## Abstract

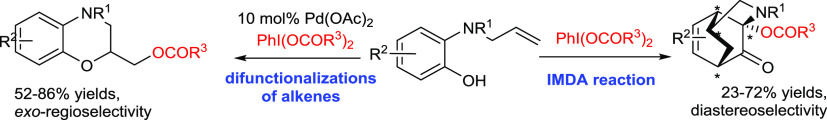

The Pd(II)-catalyzed reaction of *N*-allyl-2-aminophenols in the presence of PhI(OCOR)_2_ as the oxidant resulted in an alkoxyacyloxylation process, with the formation of functionalized dihydro-1,4-benzoxazines. The reaction performed in the absence of palladium catalyst switched to an intramolecular Diels–Alder reaction (IMDA) pathway, which was the result of an oxidative dearomatization of the 2-aminophenol, nucleophilic addition, and Diels–Alder reaction cascade, highlighting the role of the oxidant as both a nucleophilic donor and an oxidizing agent.

The construction of functionalized heteropolycyclic molecules in one step, with the advantage to avoid isolation of intermediates is an attractive goal compared to traditional stepwise synthesis.^1^ Moreover, the formation of several bonds combined in one pot complies with the concept of green chemistry in terms of timesaving and due to the reduced waste production.^2^ The interest of a domino processes may be increased by using transition metal catalysis;^3^ in particular, the use of palladium(II) catalyst in oxidative conditions offers the possibility to use unactivated substrates as the unsaturated systems.^4^ In this case, in addition to the catalyst and the solvent, the oxidant may also have a crucial role in the outcome of the reaction. The synthetic strategies for the construction of oxygen-containing heterocycles applying the palladium(II)-catalyzed reactions through the formation of the intramolecular C–O bond, starting from alcohols, phenols, or carboxylic acids, are known in the literature,^5^ while the domino processes are less explored as the alkoxyacyloxylation.^6^

Continuing our studies on the C–O bond formation exploiting Pd-catalyzed intramolecular reactions in oxidative conditions,^7^ we set out to study the reactivity of phenols bearing an unsaturated pendant, to investigate the regioselectivity and the stereoselectivity in the cyclization step. Different regioselective pathways are dependent on the *exo*- or *endo*-cyclization processes arising from the length and rigidity of the linking alkyl chain. Moreover, the choice of a substrate that is easily oxidizable, such as a phenol, in oxidative conditions, represented a challenge due to the different possible pathways that the reaction can follow.

In particular, in the present study the reported oxidative reactions allowed the construction of two different heteropolycyclic systems starting from the same substrates but mildly tuning the reaction conditions (Scheme 1.1). In both cases, the reported processes showed easy synthetic pathways to achieve important building blocks for bioactive compounds and natural products endowed with cytotoxic activity as cluvenone and gambogic acid (Scheme 1.2).^8^

**Scheme 1:**
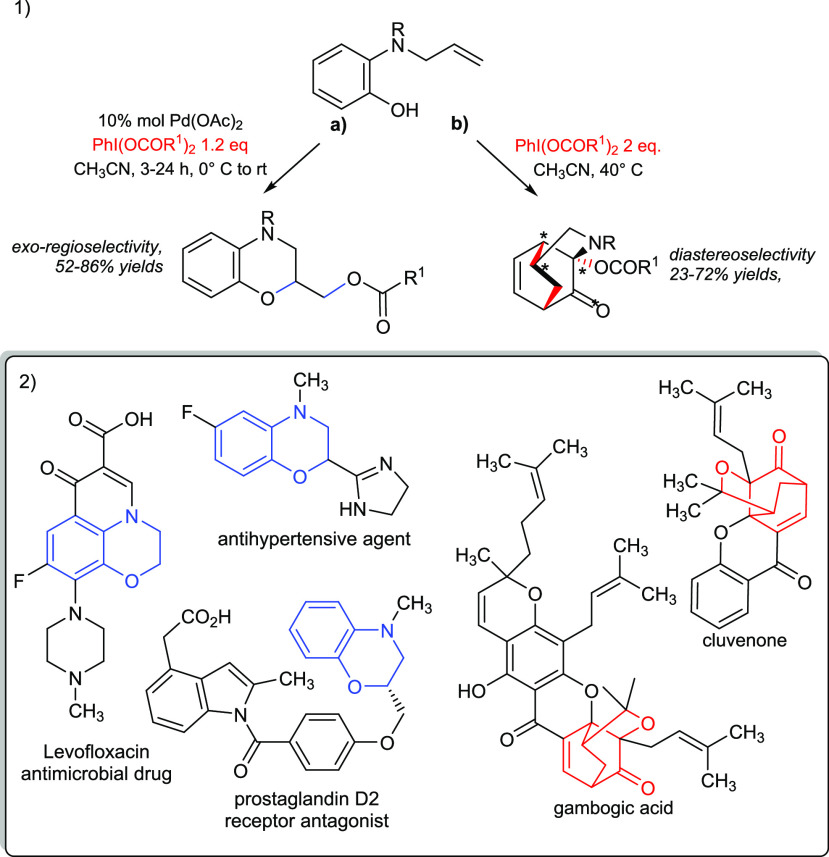
(1) Switchable Oxidative Reaction of *N*-Allyl-2-aminophenols and (2) Natural Products and Bioactive Compounds Containing the Dihydro-1,4-benzoxazine and Tricyclic Frameworks

The study started using *N*-allyl-*N*-tosyl 2-aminophenol **1a** as a substrate to test different reaction conditions. The use of Pd(OAc)_2_ in the presence of PIDA as oxidant in CH_3_CN as solvent at room temperature (RT) afforded dihydro-1,4-benzoxazine **3** as the result of the alkoxyacetoxylation process (Table 1, entry 1).

**Table 1:**
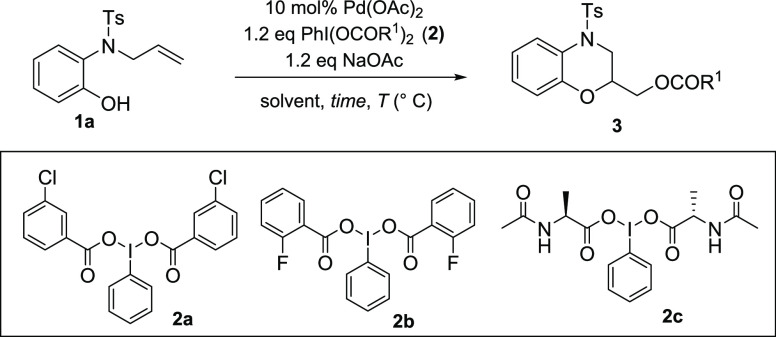

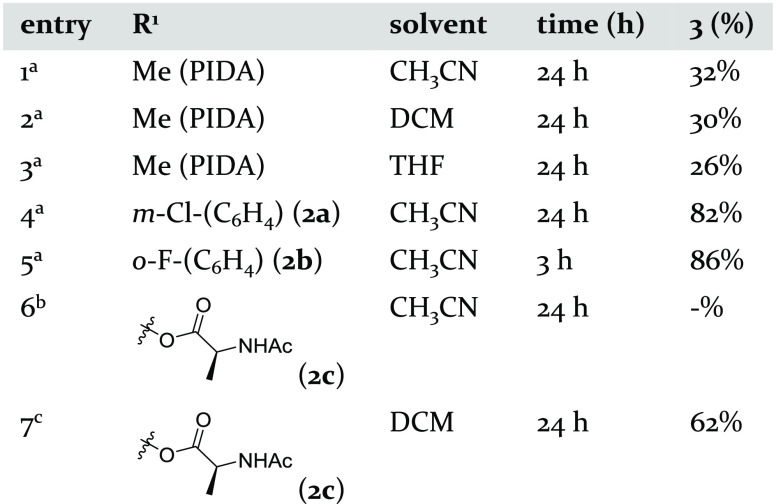
Alkoxyacyloxylation Reaction on *N*-Allyl-*N*-Ts-2-aminophenol **1a**

a Reaction conditions: 10 mol % Pd(OAc)_2_, 1.2 equiv of **2a** or **2b** in CH_3_CN at RT.

b Reaction conditions: 10 mol % Pd(OAc)_2_, 1.2 equiv of **2c** in CH_3_CN at RT.

c Reaction conditions: 10 mol % Pd(OAc)_2_, 1.2 equiv of **2c** in DCM at 40 °C.

The product was obtained with complete regioselectivity through a 6-*exo*-*trig* cyclization but in low yield. Different solvents, as DCM and THF, did not improve the yields (entries 2 and 3).With the purpose to increase yields, different functionalized hypervalent iodine reagents **2** were synthesized, due to the greater reactivity shown in the literature in alkene addition reaction^9^ or C–H functionalization reaction.^10^ Indeed, employing oxidizing agent **2a** (entry 4) and **2b** (entry 5), instead of the commercially available PIDA, the alkoxyacyloxylation reaction afforded compounds **3aa** and **3ab**, in 82 and 86% yield, respectively, in acetonitrile as solvent. The products obtained as a difunctionalization of the double bond showed the involvement of the oxidant agents also as nucleophilic donor (see the Supporting Information). Afterward, hypervalent amino acid type **2c** was prepared and used in the same reaction. In this case, the acetonitrile was not compatible with the solubility of **2c** and resulted in no reaction (entry 6). Performing the reaction in DCM as solvent at 40 °C, desired product **3ac** was instead achieved in good yield (62%), even if as an inseparable mixture of the two diastereoisomers (entry 7). The best reaction conditions were then applied to different substrates with similar good results in term of yields: *N*-Boc-derivative **1b** gave dihydro-1,4-benzoxazine **3ba**, substituted 2-amino phenols **3c**–**e** afforded the cyclized products (**3cb**–**eb**) in very good yields, 3-amino-β-naphthol **3f** gave the product of alkoxyacyloxylation **3fb**, and 2-aminobenzyl alcohol **4a** afforded tetrahydro-1,4-benzoxazepine **4aa** (Scheme 2).

**Scheme 2:**
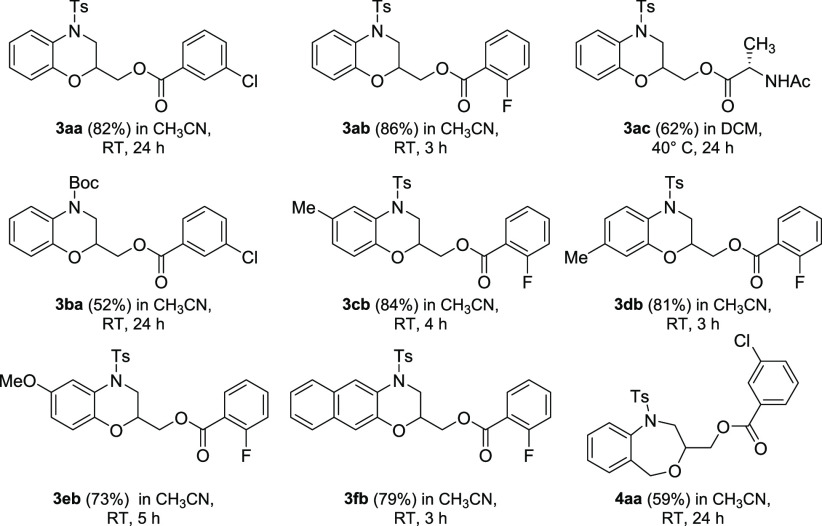
Scope for the Pd-Catalyzed Alkoxyacyloxylation Reaction

Regarding the stereocontrol of the cyclization step, few examples are reported in the literature on the difunctionalization of alkenes involving the C–O bond formation.^11^ Conversely, good enantioselectivity was reported in the aminoacetoxylation process by the crucial use of the pyridine-oxazoline (Pyox) ligands with a sterically bulky group at the C-6 position of the pyridine.^12^ Starting our investigations with the reported reaction conditions, 10 mol % Pd(OAc)_2_ PIDA in DCM (0.6 M) and ligand **L1**,^12a^ the alkoxyacetoxylated product was not even achieved (entry 1, Table 2). Also, the replacement with the more reactive PhI(mcba)_2_ did not afford the expected results (entries 2 and 3, Table 2). Only when we employed acetonitrile as solvent (entry 4) was expected ester derivative **3aa** achieved in excellent yield but with a very low enantiomeric excess. The explanation of this behavior may depend on the acetonitrile properties, through the interaction of the nitrile with the palladium species affecting the formation of the Pd–ligand complex.^13^ In order to improve the stereoselectivity, the reaction of **1a** was performed at −20 °C, with better stereoselectivity but low yield (entry 5). Using a mixture 1:5 of CH_3_CN/toluene as solvent, in the presence of **L2**, no product **3aa** was formed but a different compound **5aa** was observed (entry 6, Table 2). Replacing ligands **L1** and **L2** with commonly used ligand **L3**, product **3aa** was barely achieved, and compound **5aa** was the major product (entry 7). Similar results were obtained with *N*-oxide ligand **L4** (entry 8), known to be able to combine with Lewis acids to catalyze asymmetric difunctionalization of alkenes.^14^ Remarkably, the use of **L5** ligand afforded compound **5aa** as the exclusive product (entry 9). The analytical and spectroscopic data revealed the structure of **5aa** as a tricyclic product, confirmed by X-ray diffraction analysis.

**Table 2:**
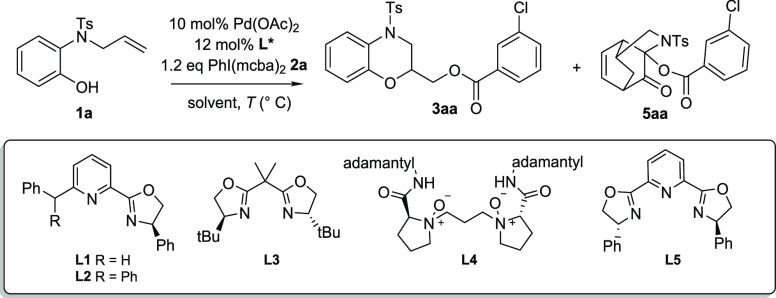
Investigations on the Stereoselective Pd-Catalyzed Alkoxyacyloxylation of *N*-Allyl-*N*-Ts-2-aminophenol **1a**

entry	lig	solvent	T(° C)	**3aa** (%)	**5aa** (%)
1^a^	**L1**	DCM	RT		
2	**L1**	DCM	RT		
3	**L2**	DCM	0 °C		
4	**L2**	CH_3_CN	0 °C	59% er 55:45	
5	**L2**	CH_3_CN	–20 °C	23% er 65:35	
6	**L2**	CH_3_CN/CH_3_Ph 1:5	0 °C	traces	29%
7	**L3**	CH_3_CN	RT	15%	49%
8	**L4**	CH_3_CN	RT	10%	43%
9	**L5**	CH_3_CN	RT		64%
10	b	CH_3_CN	40 °C		71%

a In the presence of PIDA instead of PhI(mcba)_2_.

b In the absence of Pd.

The use of not suitable Pd(II)-ligands favored a reaction promoted exclusively by the hypervalent iodine. Indeed, under the conditions of Table 2, entries 7–9, compound **5aa** was achieved as major or exclusive product. Thus, the explanation could rely on the inability of ligands **L3**–**L5** to keep the electrophilicity of Pd(II), kidnapping the palladium and promoting a hypervalent iodine-based reaction.^15^ In fact, repeating the reaction in the absence of palladium and in the presence only of hypervalent iodine **2b**, compound **5aa** was obtained as the exclusive product with 71% yields (entry 10, Table 2). Thus, the mechanism suggested for the formation of functionalized tricyclic system **5aa** is reported in Scheme 3. The first step includes an oxidation/dearomatization of the 2-aminophenol induced by the coordination of the iodine to the phenolic oxygen with the formation of *ortho*-quinone form **B**. The subsequent attack of the nucleophile gives intermediate **D**, followed by the intramolecular Diels–Alder reaction involving the allyl substituent, affording the tricyclic system. The process is fully diastereoselective with the formation of only one diastereoisomer. The good result still achieved the formation of product **5aa** in the presence of Pd-catalyst and **L5** (entry 9, Table 2), may be also due to the stabilization of the quinone intermediate **C**, mediated by the Pd-ligand complex, according to a mechanism proposed by Sigman^16^ (Scheme 3).

**Scheme 3:**
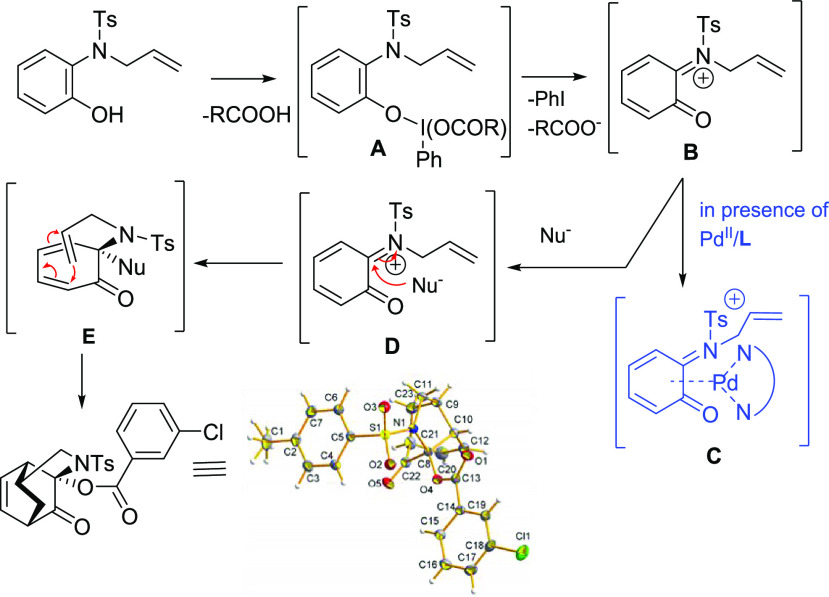
Proposed Mechanism for the Formation of Compound **5aa**

In the last years, the intramolecular Diels–Alder reactions in combination with other reactions have been fully investigated in domino/tandem processes,^17^ but to the best of our knowledge, no use of oxidant agent as nucleophile was reported. Thus, we described a mild procedure for the achievement of different functionalized tricyclic structures, simply varying the hypervalent iodine species (Scheme 1.1b). The results are reported in Table 3. By using the hypervalent iodine agents, **2a**–**c**, corresponding α-amino-functionalized tricycles **5aa**–**ac** were obtained in good yields (entries 2–5). When a good nucleophile was employed, such as the benzimidazole, the reaction could be carried out by using PhI(OAc)_2_ and 1.2 equiv of benzimidazole in CH_3_CN (entry 6). Hence, the reaction was applied on *N*-allyl-2-aminophenol bearing different *N*-protecting groups, **1b**,**i**,**j**, and on different substituted substrates, **1c**–**g**. While the Boc-derivative (**1b**) was degraded and the trifluoroacetic-group (**1i**) was not reactive, amide **1j** gave good results. Regarding the substitution on the ring, only *para*-substitution at the amino group was tolerated, affording products **5da** and **5ga** with 52 and 57% yields, respectively. In order to check the need to prefunctionalize the hypervalent iodine, a control reaction was carried out in the presence of PIDA and *m*-chlorobenzoic acid as an external nucleophile (entry 3, Table 3). The achievement of a mixture of both functionalized systems, with the *m*-chlorobenzoate and the acetoxy group, respectively, confirmed the need to preinstalled the nucleophile of interest into the iodine(III).

**Table 3:**
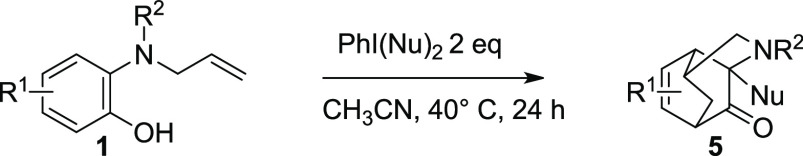

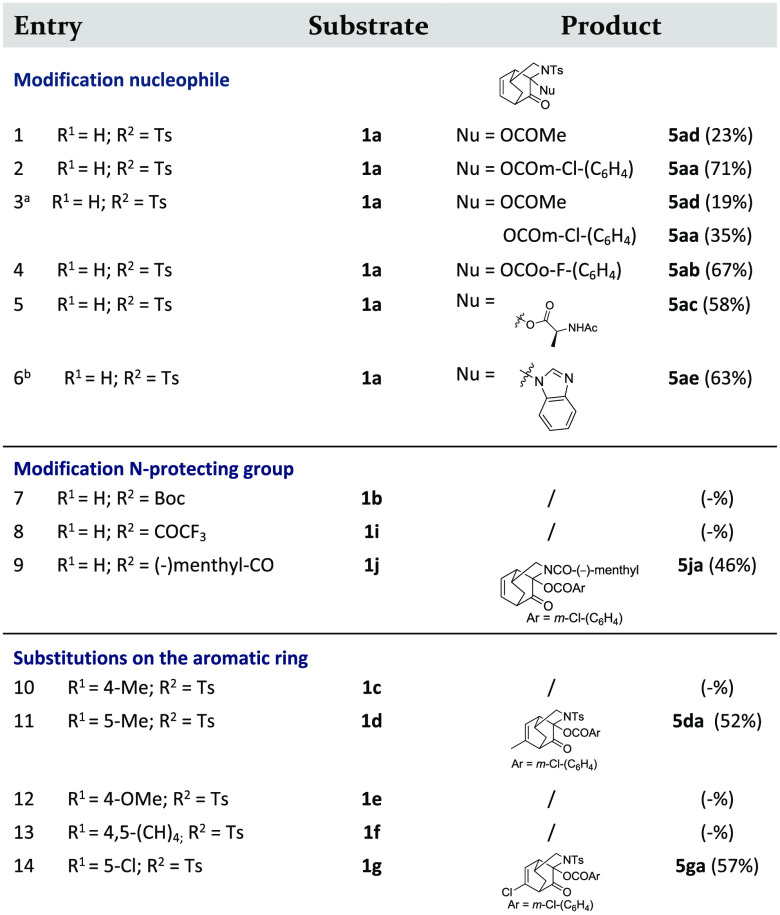
Scope of the Intramolecular Diels–Alder Reaction

a PhI(OAc)_2_ (1.5 equiv) and *m*-chlorobenzoic acid (1.5 equiv) were used instead of PhI(mcba)_2_.

b PhI(OAc)_2_ (2 equiv) and benzimidazole (1.5 equiv) were used.

In conclusion, we described a useful reactivity of *N*-allyl-2-aminophenols under Pd-catalysis in oxidative conditions, by the use of uncommon hypervalent iodines. By tuning the reaction conditions, it was possible to switch between the two processes, the intra/interdifunctionalization of the double bond resulting in the methylacyloxylated dihydro-1,4-benzoxazines and the functionalized tricyclic system achieved through dearomatization of the substrate and intramolecular Diels–Alder reaction (IMDA).

## Supporting Information Available

Experimental procedures, compound characterization data including copies of ^1^H and ^13^C NMR spectra (PDF)

## Accession Codes

CCDC 2078893 contains the supplementary crystallographic data for this paper. These data can be obtained free of charge via www.ccdc.cam.ac.uk/data_request/cif, or by emailing data_request@ccdc.cam.ac.uk, or by contacting The Cambridge Crystallographic Data Centre, 12 Union Road, Cambridge CB2 1EZ, UK; fax: +44 1223 336033.
