# Supplementary material for: Switchable Oxidative Reactions of N-allyl-2-Aminophenols: Palladium-Catalyzed Alkoxyacyloxylation vs an Intramolecular Diels–Alder Reaction
Source: Org Lett. 2021 Sep 27;23(20):7698–702. doi: 10.1021/acs.orglett.1c02539 (PMC8524420; doi:10.1021/acs.orglett.1c02539)

Supporting Information

Switchable Oxidative Reactions of *N*-allyl-2-Aminophenols: Palladium-Catalyzed Alkoxyacyloxylation *vs* an Intramolecular Diels-Alder Reaction

Sabrina Giofrè,[a] Manfred Keller,[c] Leonardo Lo Presti,[b] Egle M. Beccalli [a]*, Letizia Molteni [a]

[a]*DISFARM, Sezione di Chimica Generale e Organica "A. Marchesini", Università degli Studi di Milano, Via Venezian 21, 20133, Milano (Italy).*

[b]*Dipartimento di Chimica, Università degli Studi di Milano, Via Golgi 19, 20133, Milano (Italy).*

[c]*Institut für Organische Chemie, Albert-Ludwigs-Universität Freiburg, Albertstr. 21, 79104 Freiburg im Breisgau (Germany)*

Corresponding author:

Egle Maria Beccalli: egle.beccalli@unimi.it

Contents

General Information 2
General Procedures 3
General procedure for the preparation of C-6 modified Pyox ligand (GP1) 3
General procedure for the preparation of differently substituted hypervalent iodine (III) (GP2) 4
General procedure for the Ts-protection (GP3) 4
General procedure for the *N*-allylation of Ts-protected aminophenol (GP4) 4
General procedure for the racemic intramolecular alkoxyacyloxylation (GP5) 5
General procedure for the asymmetric intramolecular alkoxyacyloxylation (GP6) 5
General procedure for the intramolecular Diels Alder reaction (GP7) 5
Proposed mechanism of the Pd(II)-catalyzed alkoxyacyloxylation 6
Large-scale synthesis 6
Effect of substitution on the intramolecular Diels-Alder reaction 7
Further Procedures and Analytical Data of Unknown Compounds 7
X-Ray Crystallography (Prof. Leonardo Lo Presti) 28
Single crystal X-ray diffraction analysis of the compound 5aa 28
^{1}H NMR and ^{13}C NMR spectra 32

General Information

Melting points were determined by the capillary method with a Büchi B-540 apparatus and are uncorrected.

Chemicals were purchased from Sigma Aldrich and FluoroChem and used without any further purification.

IR spectra were measured with a Jasco FT/IR 5300 spectrometer, using ATR Sampling.

Elemental analyses were executed on Perkin-Elmer CHN Analyzer Series II 2400.

Nuclear Magnetic Resonance Spectroscopy (NMR)

^{1}H NMR and ^{13}C in open capillary tubes. NMR spectra were recorded with: AVANCE 400 Bruker spectrometer at 400 and 100 MHz, Varian Oxford 300 MHz spectrometer at 300 and 75 MHz and AVANCE 500 Bruker spectrometer at 500 and 125 MHz, respectively. Chemical shifts are given as δ values in ppm relative to residual solvent peaks ($CHCl_3$) as the internal reference, and the coupling constants J are reported in Hertz (Hz).

^{13}C NMR spectra are ^{1}H-decoupled and the determination of the multiplicities was achieved by the APT pulse sequence or by HSQC 2D NMR. Further investigations have been made by COSY and HSQC NMR.

High Pressure Liquid Chromatography (HPLC)

The chiral HPLC analysis were carried out by using Merck LaChrom Hitachi Pump L-7100, Hewlett Packard Series 1050 DAD, and the following chiral column: OD-H (250 x 4.60 mm, 5 µm).

Optical rotations were measured on a Perkin–Elmer 343 polarimeter at 20° C (concentration in g/mL).

Mass spectrometry

ESI mass spectra were recorded on a LCQ Advantage spectrometer from Thermo Finningan and values are reported in m/z.

Mass spectra were recorded in the analytic department of the Institute of Organic Chemistry at the University of Freiburg. Ions were given as mass to charge ratios (m/z) with relative intensities in parentheses.

High resolution mass spectrometry (HRMS) of compound **5aa** was recorded in the analytic department of the Institute of Organic Chemistry at the University of Freiburg and performed on an Executive mass spectrometer (ESI or APCI) with orbitrap analyzer from Thermo Fisher Scientific Inc. The analyzer was externally calibrated and had a resolution of M/ΔM = 20 0000 - 100 000.

General Procedures

General procedure for the preparation of C-6 modified Pyox ligand (GP1)

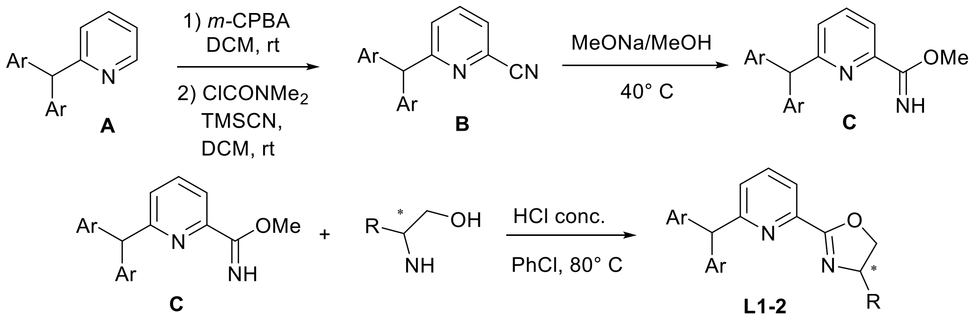

Following the procedure,[1] in a 250 mL round bottom flask, pyridine (2 mmol, 1 eq) was dissolved in DCM (0.3 M). Then *m*-CPBA (meta-chloroperoxybenzoic acid, 1.7 eq) was added, and the mixture was stirred at room temperature. After the reaction was completed, K_2CO_3 (5 eq) was added. The mixture was stirred for 30 min at room temperature. After filtered through a celite pad, the filtrate was concentrated *in vacuo* to give the desired *N*-oxide, which was used without further purification. To a solution of the crude *N*-oxide in DCM (0.7 M), dimethylcarbamic chloride (1.3 eq) was added at room temperature. The mixture was stirred for 10 min, then TMSCN (1.3 eq) was added. The mixture was stirred at room temperature. After the reaction was completed, the mixture was quenched with 10% aq K_2CO_3, and the mixture was extracted two times with DCM. The combined organic layers were washed with brine, dried over $NaSO_4$, and filtered. After concentration, the crude residue was purified by column chromatography on silica gel or crystallization to afford product **B**.

In a 50mL round bottom flask, **B** (1 eq) was dissolved in MeOH (1.5 M). Then NaOMe (0.3 eq) was added. The reaction mixture was stirred at 40° C (oil bath) overnight. After that, the solvent was removed *in vacuo*, and the residue was dissolved in EtOAc. The solution was washed with water and brine, then the organic phase was separated and dried over $NaSO_4$. After filtration, the filtrate was concentrated *in vacuo* to give the imine **C**, which was used without any further purification. The crude imine (1 eq) and (*R*)-2-amino-2-phenyl ethan-1-ol (1 eq) or (*S*)-phenylalaninol were weighted into a 100 mL round bottom flask, then dissolved in PhCl (0.5 M). Concentrated HCl (2 drops) was added to the solution and the mixture was heated to 80° C (oil bath) under nitrogen atmosphere. After the reaction was completed, the organic solvent was removed *in vacuo*. The residue was purified by column chromatography on silica gel or crystallography to afford ligand **L1-L2**.

---

[1] C. Chen, P. M. Pflgger, P. Chen, G. Liu, *Angew. Chem. Int. Ed.* **2019**, 58, 2392–2396

General procedure for the preparation of differently substituted hypervalent iodine (III) (GP2)

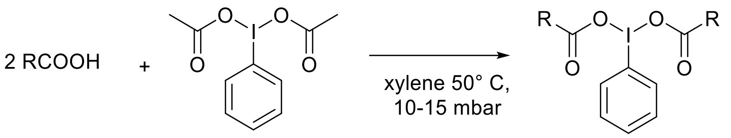

Following the procedure,[2] to a round-bottom flask, $PhI(OAc)_2$ (10 mmol, 1.0 eq.) and the corresponding acid (20 mmol, 2 eq) were dissolved in xylene (0.2 M) and the flask was heated to 50° C (water bath of rotary evaporator) under reduced pressure (about 10.15 mbar) using a diaphragm pump. When the xylene was removed, a mixture of n-hexane/AcOEt 3:1 was used to wash the solid. The white solid was then filtered and dried *in vacuo*. The corresponding hypervalent iodine (III) **2a-d** was obtained and used directly in the following reaction without any further purification.

General procedure for the Ts-protection (GP3)

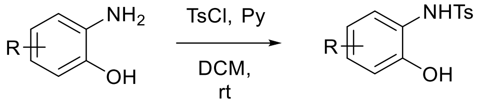

To a solution of a different substituted aniline (20 mmol, 1 eq) in DCM (0.2 M)), pyridine (3 eq) and tosyl chloride (1.3 eq.) were added at 0° C. The reaction mixture was allowed to warm to room temperature and stirred for 24 h. Then, the reaction mixture was rinsed with DCM, washed with 1 M HCl and brine. The collected organic phases were dried over Na_2SO_4, filtered and the solvent evaporated under reduced pressure. The crude product was purified by flash chromatography on silica gel or by crystallization.

General procedure for the *N*-allylation of Ts-protected aminophenol (GP4)

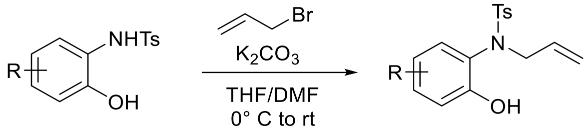

In a two-neck round-bottom flask the corresponding Ts-protected aminophenol (10 mmol, 1 eq.) was dissolved in a THF/DMF mixture (5:1, 0.2 M) and then K_2CO_3 (1.2 eq) was added. The reaction mixture was cooled to 0° C and afterwards, a solution of allyl bromide (1.2 eq) in THF (1 M) was added dropwise over 30 min. Then, the reaction was allowed to warm to room temperature and stirred for 24 h. Once the reaction has occurred as completely as possible, the mixture was filtered *in vacuo*, rinsed with AcOEt and washed with brine. The organic phase was dried over Na_2SO_4 and the solvent evaporated under reduced pressure. The crude product was purified by flash chromatography on silica gel or by crystallization.

[2] Y.Wang, L. Zhang, Y. Yang, P. Zhang, Z. Du, C. Wang, *J. Am. Chem. Soc.* **2013**, 135 (48), 18048-18051

General procedure for the racemic intramolecular alkoxyacyloxylation (GP5)

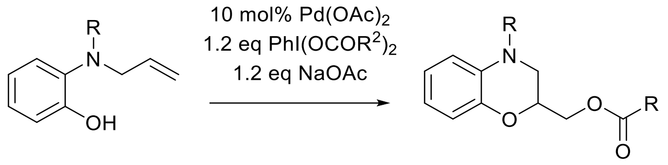

To a solution of the compound **1** (0.2 mmol, 1 eq) in the corresponding solvent (0.2 M), NaOAc (0.24 mmol, 1.2 eq), $Pd(OAc)_2$ (0.02 mmol, 10 mol%) and $PhI(OCOR^2)_2$ (0.24 mmol, 1.2 eq) were added subsequently at 0° C. The reaction mixture was stirred at rt for 3-24 h. Then, the solvent was evaporated *in vacuo*. The crude product was rinsed with EtOAc, washed with a saturated solution of $NaHCO_3$ (until pH = 8) and with brine. The organic phase was dried over Na_2SO_4, filtered and the solvent evaporated under reduced pressure. The crude product was purified by flash chromatography on silica gel.

General procedure for the asymmetric intramolecular alkoxyacyloxylation (GP6)

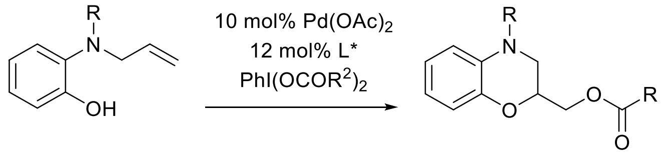

In a 5 mL glass vial $Pd(OAc)_2$ (0.03 mmol, 10 mol%) and the ligand **L1-5** (0.036 mmol, 12 mol%) were added and dissolved in the corresponding solvent (0.4 M). The complex was preformed, stirring the mixture at rt for 15 min. Then, compound **1** (0.3 mmol, 1 eq) and $PhI(mcba)_2$ (0.45 mmol, 1.2 eq) were added subsequently and the reaction was stirred at rt or cooled to 0° C or -20° C. The reaction mixture was stirred at the reported temperature for 48 h.

Once the reaction had occurred as completely as possible, the reaction mixture was immediately filtered through a plug of silica gel (3 cm) and washed with EtOAc/n-Hex 9:1. Then the collected fractions were evaporated *in vacuo*. The crude product was rinsed with EtOAc, washed with a saturated solution of $NaHCO_3$ (until pH = 8) and with brine. The organic phase was dried over Na_2SO_4, filtered and the solvent evaporated under reduced pressure. The crude product was purified by flash chromatography on silica gel.

General procedure for the intramolecular Diels Alder reaction (GP7)

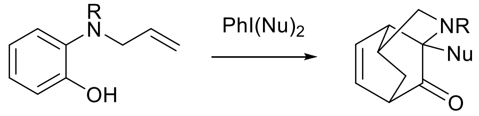

The compound **1** (0.3 mmol, 1 eq.) was dissolved in CH_3CN (0.04 M) and then $PhI(Nu)_2$ (0.6 mmol, 2 eq) was added [when benzimidazole was used as nucleophile, $PhI(OAc)_2$ (0.45 mmol, 1.5 eq) was added together with the benzimidazole (0.51 mmol, 1.7 eq)]. The reaction mixture was stirred for 24 h at 40° C (oil bath). Once the reaction had occurred as completely as possible, the solvent was evaporated *in vacuo*. The crude product was rinsed with EtOAc and washed with brine. The organic phase was dried over Na_2SO_4, filtered and the solvent evaporated under reduced pressure. The crude product was purified by flash chromatography on silica gel.

Proposed mechanism of the Pd(II)-catalyzed alkoxyacyloxylation

The proposed mechanism of the alkoxyacetoxylation process suggested metal coordination to the double bond and to the nucleophilic oxygen in order to obtain the alkoxypalladate specie **A**, which undergoes oxidation by **2** to afford the Pd(IV) intermediate **B**. Finally, the C-O bond formation leads to product and the regeneration of the catalytic reactive palladium form. In this process, the oxidizing reagent plays the dual role as nucleophilic donor and as oxidant species. On the other hand, an alternative mechanism involving the direct nucleophilic substitution of the palladium by the acyloxy group on the C(sp3) on the intermediate A, cannot be excluded.[3]

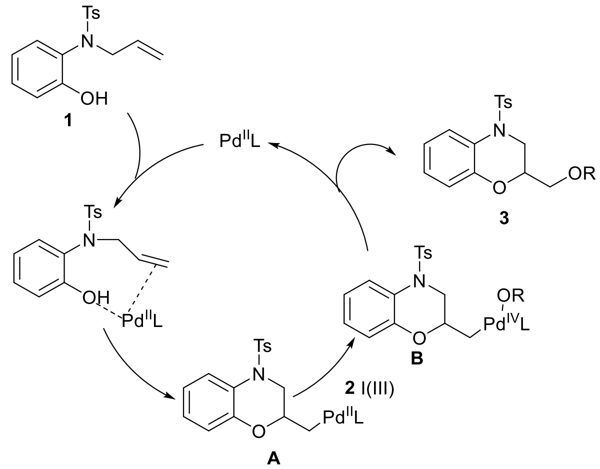

Large-scale synthesis

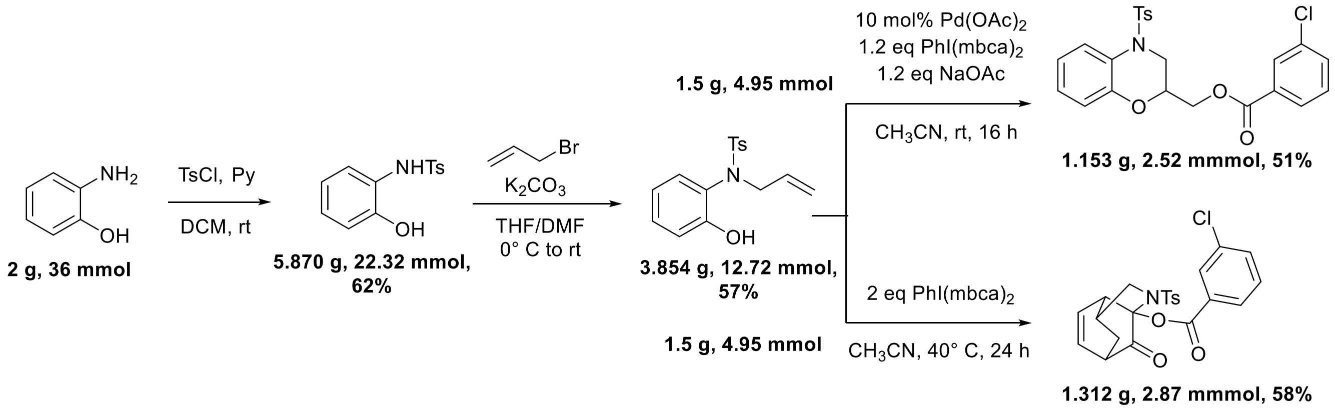

[3] a) Li, Y.; Song, Y. D.; Dong, V. M. *J. Am. Chem. Soc.*, **2008**, 130, 2962-2964; c) Wang, W.; Wang, F.; Shi, M. *Organometallics*, **2010**, 29, 928-933; f) Takenaka, K.; Dhage, Y.D.; Sasai, H. *Chem. Commun.*, **2013**, 49, 11224-11226;

Effect of substitution on the intramolecular Diels-Alder reaction

As mentioned in the manuscript, the tosyl is the most tolerated protecting group on the nitrogen for the intramolecular Diels-Alder reaction (6 examples, 52-71% yields). A reason may be ascribed to the less withdrawing properties of the sulphonyl group compared to the amide/carbamate group,[4] favouring the formation of the key intermediate **B**.

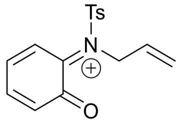

B

Indeed, in the case of the trifluoroacetyl substituted aminophenol **1i** the starting material was recovered. On the other hand, the differences observed between the Boc- and the menthyl-group are attributable to the different stability. In the reported reaction conditions, the former completely degraded, while the latter, more stable towards hydrolysis, afforded the desired product in 46% yields.

In the case of the *N*-allyl aminophenols with *p*-substitution to the oxygen group, **1c,e-f**, the presence of a substituent seems to hinder the formation of the key intermediate **B**, likely due to the formation of a *p*-quinone intermediate form. Indeed, with the methoxy-substituted derivative **1e** we could observe the demethylation of the methoxy group, as possible consequence of the *p*-quinone form. In the other two cases degradation was observed.

Further Procedures and Analytical Data of Unknown Compounds

(*R*)-2-(6-benzylpyridin-2-yl)-4-phenyl-4,5-dihydrooxazole (L1)

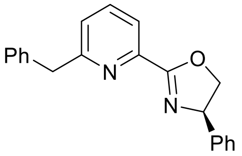

Compound **L1** was prepared according to the general procedure (GP1) and isolated as white solid (433 mg, 1.38 mmol, overall yield 69%) after flash chromatography (n-Hex/AcOEt 6:1 → 4:1) for the first purification step and (n-Hex/AcOEt 3:1) for the second one.

The data are in good agreement with those reported in the literature.[5]

---

[4] I. Chataigner, C. Panel, H. Gerard, S. R. Piettre, *Chem. Commun.*, **2007**, 3288–3290

[5] X. Qi, C. Chen, C. Hou, L. Fu, P. Chen, G. Liu, *J. Am. Chem. Soc.*, **2018**, 140 (24), 7415-7419

(*R*)-2-(6-benzhydrylpyridin-2-yl)-4-phenyl-4,5-dihydrooxazole (L2)

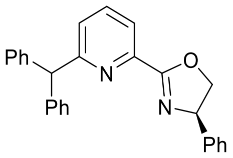

Compound **L2** was prepared according to the general procedure (GP1) and isolated as white solid (491 mg, 1.26 mmol, overall yield 63%) after flash chromatography (Hex:AcOEt 8:1) for the first purification step and (Hex:AcOEt 4:1) for the second one

The data are in good agreement with those reported in the literature.[1]

phenyl-λ3-iodanediyl bis(3-chlorobenzoate) (2a)

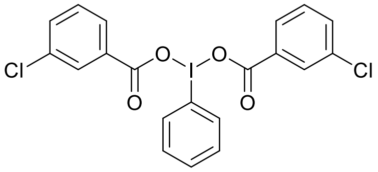

Compound **2a** was prepared according to the general procedure (GP2) and isolated as white solid (4,98 g, 9.67 mmol, yield 97%).

The data are in good agreement with those reported in the literature.[6]

phenyl-λ3-iodanediyl bis(2-fluorobenzoate) (2b)

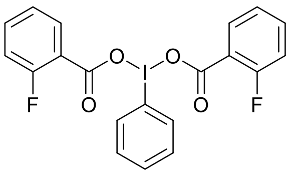

Compound **2b** was prepared according to the general procedure (GP2) and isolated as white solid (4.57 g, 9.56 mmol, yield 95%).

The data are in good agreement with those reported in the literature. [6]

phenyl-λ3-iodanediyl (2*S*,2'*S*)-bis(2-acetamidopropanoate) (2c)

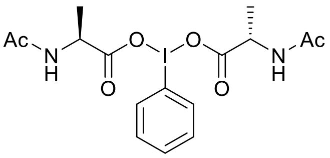

[6] K. Muñiz B. García C. Martínez, A. Piccinelli, *Chem Eur J,* **2017**, 23(7), 1539-1545

Compound **2c** was prepared according to the general procedure (GP2) and isolated as white solid (3.851 g, 8.32 mmol, yield 83%).

The data are in good agreement with those reported in the literature.[7]

N-(2-hydroxyphenyl)-4-methylbenzenesulfonamide

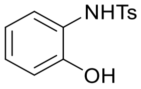

N-(2-hydroxyphenyl)-4-methylbenzenesulfonamide was prepared according to the general procedure (GP3) and isolated as yellow solid (3.31 g, 12.56 mmol yield 65%) after crystallization (Hex/EtOAc).

The data are in good agreement with those reported in the literature.[8]

N-(4-chloro-2-hydroxyphenyl)-4-methylbenzenesulfonamide

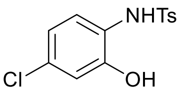

N-(4-chloro-2-hydroxyphenyl)-4-methylbenzenesulfonamide was prepared according to the general procedure (GP3) and isolated as brown solid (4.21 g, 14.23 mmol, yield 71%) after flash column chromatography (Hex/EtOAc 3:1 → 2:1).

The data are in good agreement with those reported in the literature.[8]

N-(2-hydroxy-4-methylphenyl)-4-methylbenzenesulfonamide

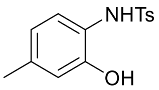

N-(2-hydroxy-4-methylphenyl)-4-methylbenzenesulfonamide was prepared according to the general procedure (GP3) and isolated as white solid (4.59 g, 16.59 mmol, yield 83%) after flash column chromatography (Hex/EtOAc 2:1).

The data are in good agreement with those reported in the literature.[8]

N-(2-hydroxy-5-methylphenyl)-4-methylbenzenesulfonamide

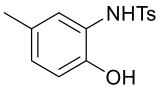

N-(2-hydroxy-5-methylphenyl)-4-methylbenzenesulfonamide was prepared according to the general procedure (GP3) and isolated as white solid (4.70 g, 17.01 mmol, yield 85%) after flash column chromatography (Hex/EtOAc 3:1).

---

[7] *Zhurnal Organicheskoi Khimii,* **1975,** 11 (6), 1259-1263

[8] K. Wen, Z. Wu, B. Huang, Z. Ling, I. D. Gridnev, W. Zhang*, Org. Lett.,* **2018,** 206, 1608-1612

The data are in good agreement with those reported in the literature.[8]

N-(2-hydroxy-5-methoxyphenyl)-4-methylbenzenesulfonamide

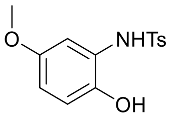

N-(2-hydroxy-5-methoxyphenyl)-4-methylbenzenesulfonamide was prepared according to the general procedure (GP3) and isolated as white solid (4.04 g, 13.76 mmol, yield 69%) after flash column chromatography (Hex/EtOAc 3:1 → 2:1).

The data are in good agreement with those reported in the literature.[9]

N-(3-hydroxynaphthalen-2-yl)-4-methylbenzenesulfonamide

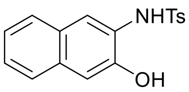

N-(3-hydroxynaphthalen-2-yl)-4-methylbenzenesulfonamide was prepared according to the general procedure (GP3) and isolated as brown solid (3.19 g, 10.22 mmol, yield 51%) after flash column chromatography (Hex/EtOAc 3:1 → 1:1).

The data are in good agreement with those reported in the literature.[8]

N-allyl-*N*-(2-hydroxyphenyl)-4-methylbenzenesulfonamide (1a)

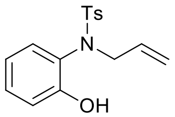

Compound **1a** was prepared according to the general procedure (GP4) and isolated as white solid (1.94 g. 6.38 mmol, yield 64%) after flash chromatography (Hex:AcOEt 6:1).

M.p. 110.0–112.5° C

IR: 3451, 2919, 1643, 1594, 1493 cm^{-1}

^{1}H-NMR (300 MHz, $CDCl_3$) δ: δ 7.59 – 7.44 (m, 2H), 7.27 (dd, *J* = 5.1, 4.7 Hz, 2H), 7.23 – 7.11 (m, 1H), 7.03 (dd, *J* = 8.2, 1.5 Hz, 1H), 6.68 (ddt, *J* = 17.3, 11.3, 5.7 Hz, 1H), 6.59 (s, 1H), 6.36 (dd, *J* = 8.0, 1.6 Hz, 1H), 5.72 (ddt, *J* = 17.4, 9.8, 6.5 Hz, 1H), 5.06 (ddd, *J* = 11.2, 2.4, 1.3 Hz, 2H), 4.16 (br s, 2H), 2.44 (s, 3H).

^{13}C-NMR (75 MHz, $CDCl_3$) δ: 154.8 (s), 144.3 (s), 133.7 (s), 131.8 (d), 129.9 (d), 129.6 (d); 128.1 (d), 127.5 (d), 125.7 (s), 120.3 (d), 119.9 (t), 117.4 (d), 54.6 (t), 21.6 (q).

MS (ESI): m/z 326.37 $[M+Na]^+$

---

[9] H. Shen, Y.F. Wu, Y. Zhang, L.F. Fan, Z.Y. Han, L.Z. Gong, Angew. *Chem, Int Ed*, **2018**, 57, 2372-2376

Anal. Calcd for $C_{16}H_{17}NO_3S$ C, 63.34; H, 5.65; N, 4.62. Found: C, 63.31; H, 5.61; N, 4.66.

Synthesis of *tert*-butyl allyl(2-hydroxyphenyl)carbamate (1b)

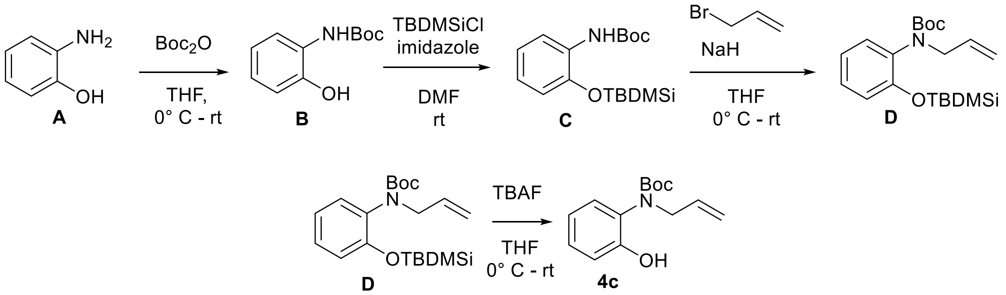

To a two-neck round-bottom flask 2-aminoethanol **A** (1500 mg, 13.76 mmol, 1 eq) was added and dissolved in THF (13 mL, 1 M). After cooling to 0° C, di-*tert*-butyl dicarbonate (3150 mg, 14.4 mmol, 1.05 eq) was added. The solution was allowed to warm to rt and stirred for 13 h. Afterwards, the solvent was evaporated under reduced pressure and the remaining solid was washed with CCl_4 to yield the *N*-Boc-protected aminophenol **B** (2007 mg, 12.93 mmol, 94%) as a white solid.

A solution of *N*-protected aminophenol **B** (14.4 mmol, 1 eq), tert-butyldimethylsilyl chloride (14.4 mmol, 1 eq) and imidazole (14.4 mmol, 1 eq) in DMF (0.2 M) was stirred at room temperature overnight. The reaction was taken up with brine (50 mL), extracted with Et_2O (3 x 50 mL), dried over Na_2SO_4 and concentrated at reduced pressure. The crude product was purified by silica gel column chromatography gel (n-Hex/EtOAc 5:1) to afford **C** (2402 mg, 7.17 mmol, 49%).

To a mixture of sodium hydride (10.8 mmol, 1.5 eq) in DMF (18 mL) at 0 °C under argon atmosphere, a solution of **C** (2402 mg, 7.17 mmol, 1 eq) in THF (3 mL) was dropped. The reaction mixture was stirred for 30 min at room temperature, then cooled at 0 °C. A solution of allyl bromide (7.52 mmol, 1.05 eq) in THF (7 mL) was dropped and the mixture was stirred at room temperature overnight. The mixture was quenched with water (10 mL) and concentrated at reduced pressure. Then, the crude mixture was rinsed with EtOAc (3 x 40 mL), washed with brine (3 x 20 mL), dried over Na_2SO_4, filtered and concentrated at reduced pressure. The crude product was purified by silica gel column chromatography (Hex:AcOEt 4:1) to afford **D** (1398 mg, 3.72 mmol, 52%).

A mixture of the corresponding **D** (1398 mg, 3.72 mmol, 1 eq) and tetrabutylammonium fluoride (4.46 mmol, 1.2 eq) in THF (0.2 M) was stirred at room temperature for 3 h. The solvent was evaporated under reduced pressure; water was added (10 mL). The reaction mixture was extracted with DCM (3 x 20 mL), then dried over Na_2SO_4, and concentrated at reduced pressure. The crude product was purified by silica gel column chromatography (Hex:AcOEt 4:1) to afford **1b** (794 mg, 3.19 mmol, 86%) as a white solid.

tert-butyl allyl(2-hydroxyphenyl)carbamate (1b)

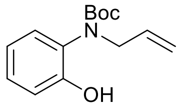

M.p. 134–136° C

IR: 3249, 1649, 1396, 761 cm^{-1}

^{1}H NMR (300 MHz, $CDCl_3$) δ 7.15 (t, *J* = 7.0 Hz, 2H), 7.00 (d, *J* = 7.6 Hz, 1H), 6.95 – 6.83 (m, 1H), 5.92 (ddd, *J* = 15.9, 10.7, 5.6 Hz, 1H), 5.33 – 5.06 (m, 2H), 4.20 (d, *J* = 5.4 Hz, 2H), 1.48 (s, *J* = 13.8 Hz, 9H).

MS (ESI): m/z 273.35 $[M+Na]^+$

Anal. Calcd for $C_{14}H_{19}NO_3S$ C, 67.45; H, 7.68; N, 5.62. Found: C, 67.49; H, 7.64 ; N, 5.66 .

N-allyl-*N*-(2-hydroxy-5-methylphenyl)-4-methylbenzenesulfonamide (1c)

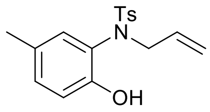

Compound **1c** was prepared according to the general procedure (GP4) and isolated as white solid (2.59 g, 8.24 mmol, yield 82%) after flash chromatography (Hex/AcOEt 4:1).

R_f = 0.35 (n-Hex/EtOAc 2:1); stained with $KMnO_4$

M.p. 123–125° C

IR = 3439, 1594, 1505, 832, 817 cm^{-1}

^{1}H NMR (300 MHz, $CDCl_3$) δ 7.54 (d, *J* = 8.3 Hz, 2H), 7.28 (d, *J* = 8.0 Hz, 2H), 7.04 – 6.82 (m, 2H), 6.38 (s, 1H), 6.15 (d, *J* = 1.8 Hz, 1H), 5.72 (ddt, *J* = 16.6, 10.1, 6.5 Hz, 1H), 5.12 – 4.98 (m, 2H), 4.12 (br s, 2H), 2.44 (s, 3H), 2.06 (s, 3H).

^{13}C NMR (75 MHz, $CDCl_3$) δ 152.4 (s), 144.2 (s), 134.1 (s), 132.0 (d), 130.5 (d), 129.7 (s), 129.4 (d), 128.1 (d), 127.9 (d), 125.4 (s), 119.5 (t), 117.0 (d), 54.5 (t), 21.5 (q), 20.3 (q).

MS (ESI): m/z 318.45 $[M+H]^+$

Anal. Calcd for $C_{17}H_{19}NO_3S$ C, 64.33; H, 6.03; N, 4.41. Found: C, 64.30; H, 5.99; N, 4.44.

N-allyl-*N*-(2-hydroxy-4-methylphenyl)-4-methylbenzenesulfonamide (1d)

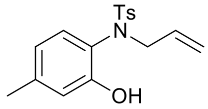

Compound **1d** was prepared according to the general procedure (GP4) and isolated as pale-yellow solid (2.35 g, 7.36 mmol, yield 74%) after flash chromatography (Hex/AcOEt 4:1).

R_f = 0.36 (n-Hex/EtOAc 2:1); stained with $KMnO_4$

M.p. 118–120° C

IR = 3422, 2978, 1534 cm^{-1}

^{1}H NMR (300 MHz, $CDCl_3$) δ 7.54 (d, *J* = 8.3 Hz, 2H), 7.34 – 7.20 (m, 2H), 6.87 – 6.80 (m, 1H), 6.52 – 6.42 (m, 2H), 6.23 (d, *J* = 8.1 Hz, 1H), 5.81 – 5.59 (m, 1H), 5.12 – 4.99 (m, 2H), 4.13 (br s, 2H), 2.44 (s, 3H), 2.26 (s, 3H).

^{13}C NMR (75 MHz, $CDCl_3$) δ 154.4 (s), 144.1 (s), 140.3 (s), 134.0 (s), 132.0 (d), 129.5 (d), 128.1 (d), 127.1 (d), 123.0 (s), 121.2 (d), 119.6 (t), 117.8 (d), 54.6 (t), 21.6 (q), 21.3 (q).

MS (ESI): m/z 317.78 $[M+H]^+$

Anal. Calcd for $C_{17}H_{19}NO_3S$ C, 64.33; H, 6.03; N, 4.41. Found: C, 64.36; H, 6.00; N, 4.38.

N-allyl-*N*-(2-hydroxy-5-methoxyphenyl)-4-methylbenzenesulfonamide (1e)

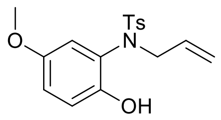

Compound **1e** was prepared according to the general procedure (GP4) and isolated as white solid (2.19 g. 6.64 mmol, yield 66%) after flash chromatography (Hex/AcOEt 4:1).

R_f = 0.26 (n-Hex/EtOAc 2:1); stained with $KMnO_4$

M.p. 117–119° C

^1H NMR (300 MHz, $CDCl_3$) δ 7.55 (d, J = 8.3 Hz, 2H), 7.29 (d, J = 8.0 Hz, 2H), 6.96 (d, J = 9.0 Hz, 1H), 6.76 (dd, J = 9.0, 3.0 Hz, 1H), 6.24 (s, 1H), 5.90 (d, J = 3.0 Hz, 1H), 5.73 (ddt, J = 16.6, 10.1, 6.5 Hz, 1H), 5.19 – 4.97 (m, 2H), 4.15 (br s, 2H), 3.52 (s, 3H), 2.44 (s, 3H).

^{13}C NMR (75 MHz, $CDCl_3$) δ 153.0 (s), 148.8 (s), 144.3 (s), 134.0 (s), 131.9 (d), 129.6 (d), 128.1 (d), 125.9 (s), 119.7 (t), 117.9 (d), 115.78 (d), 112.9 (d), 55.65 (q), 54.5 (t), 21.5 (q).

MS (ESI): m/z 357.23 $[M+Na]^+$

Anal. Calcd for $C_{17}H_{19}NO_4S$ C, 61.26; H, 5.74; N, 4.20 . Found: C, 61.31; H, 5.72; N, 4.18.

N-allyl-*N*-(3-hydroxynaphthalen-2-yl)-4-methylbenzenesulfonamide (1f)

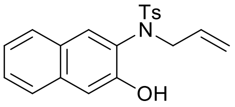

Compound **1f** was prepared according to the general procedure (GP4) and isolated as brown gum (2.33 g, 6.56 mmol, yield 66%) after flash chromatography (Hex/AcOEt 5:1 → 3:1).

R_f = 0.28 (n-Hex/EtOAc 2:1); stained with $KMnO_4$

^1H NMR (300 MHz, $CDCl_3$) δ 7.76 – 7.63 (m, 1H), 7.60 – 7.48 (m, 2H), 7.48 – 7.37 (m, 3H), 7.33 – 7.25 (m, 3H), 6.87 (s, 1H), 6.58 (s, 1H), 5.75 (ddt, J = 16.7, 10.1, 6.5 Hz, 1H), 5.13 – 4.99 (m, 2H), 4.26 (br s, 2H), 2.46 (s, 3H).

^{13}C NMR (75 MHz, $CDCl_3$) δ 152.0 (s), 144.4 (s), 134.4 (s), 133.6 (s), 131.6 (d), 129.6 (d), 128.3 (d), 128.1 (s), 127.9 (s), 127.5 (d), 127.4 (d), 127.2 (d), 126.5 (d), 123.8 (d), 120.1 (t), 112.1 (d), 55.2 (t), 21. (q).

MS (ESI): m/z 376.13 $[M+Na]^+$

Anal. Calcd for $C_{20}H_{19}NO_3S$ C, 67.97; H, 5.42; N, 3.96. Found: C, 67.94; H, 5.45; N, 3.95.

N-allyl-*N*-(4-chloro-2-hydroxyphenyl)-4-methylbenzenesulfonamide (1g)

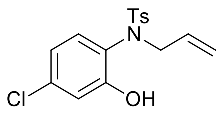

Compound **1g** was prepared according to the general procedure (GP4) and isolated as pale-yellow solid (2.38 g, 7.14 mmol, yield 71%) after flash chromatography (Hex/AcOEt 4:1).

R_f = 0.33 (n-Hex/EtOAc 2:1); stained with $KMnO_4$

M.p. 91–92° C

IR: 3311, 1623, 1541, 1343 cm^{-1}

^{1}H NMR (300 MHz, $CDCl_3$) δ 7.54 (d, *J* = 8.3 Hz, 2H), 7.32 (d, *J* = 8.0 Hz, 2H), 7.15 (dd, *J* = 8.8, 2.5 Hz, 1H), 6.98 (d, *J* = 8.8 Hz, 1H), 6.61 (d, *J* = 5.5 Hz, 1H), 6.31 (d, *J* = 2.5 Hz, 1H), 5.70 (ddt, *J* = 17.2, 9.8, 6.5 Hz, 1H), 5.12 – 4.99 (m, 2H), 4.11 (br s, 2H), 2.46 (s, 3H).

^{13}C NMR (75 MHz, CDC_3) δ 153.7 (s), 144.7 (s), 133.3 (s), 131.4 (d), 129.9 (d), 129.7 (d), 128.1 (d), 127.5 (d), 126.7 (s), 124.5.5 (s), 120.2 (t), 118.5 (d), 54.6 (t), 21.6 (q).

MS (ESI): m/z 337.95 [M+H]$^+$

Anal. Calcd for $C_{16}H_{16}ClNO_3S$ C, 56.89; H, 4.77; N, 4.15. Found: C, 56.93; H, 4.75; N, 4.12.

N-allyl-*N*-(2-(hydroxymethyl)phenyl)-4-methylbenzenesulfonamide (1h)[10]

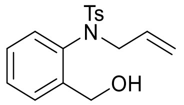

N-allyl-*N*-(2-(hydroxymethyl)phenyl)-4-methylbenzenesulfonamide was prepared according to the general procedure (GP4) and isolated as white solid (2.13 g, 6.73 mmol, yield 67%) after flash chromatography (Hex:AcOEt 5:1).

The data are in good agreement with those reported in the literature.[10]

M.p. 74–75° C

IR: 3509, 2920, 1335, 1158, 1010, 666 cm^{-1}

^{1}H NMR (300 MHz, $CDCl_3$) δ 7.63 – 7.49 (m, 3H), 7.40 – 7.27 (m, 3H), 7.20 – 7.05 (m, 1H), 6.44 (dd, *J* = 8.0, 1.2 Hz, 1H), 5.88 – 5.53 (m, 1H), 5.12 – 4.83 (m, 3H), 4.65 – 4.41 (m, 2H), 3.73 (dd, *J* = 13.4, 8.0 Hz, 1H), 3.12 – 2.79 (m, 2H), 2.46 (s, 3H).

^{13}C NMR (75 MHz, $CDCl_3$) δ δ 143.9 (s), 142.0 (s), 137.1 (s), 134.7 (s), 131.9 (d), 131.0 (d), 129.4 (d), 129.0 (d), 128.2 (d), 128.1 (d), 127.6 (d), 119.9 (t), 61.2 (t), 55.1 (t), 21.5 (q).

MS (ESI): m/z 340,58 [M+Na]$^+$

[10] M. Hossen, C.J. Lovely, H.V.R. Dias *Tetrahedron* **2001**, *57*, 4095-4105.

(4-tosyl -3,4-dihydro-2H-benzo[b][1,4]oxazin-2-yl)methyl 3-chlorobenzoate (3aa)

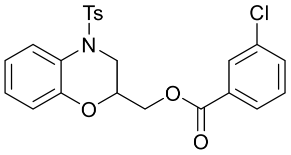

Compound **3aa** was prepared according to the general procedure (GP5, CH_3CN, 24h) and isolated as white solid (74 mg, 0.16 mmol, yield 82%) after flash chromatography (Hex:AcOEt 4:1).

R_f = 0.38 (n-Hex/EtOAc 2:1); stained with $KMnO_4$

M.p. 102–104° C

IR = 1714, 1487, 731 cm^{-1}

^{1}H NMR (300 MHz, $CDCl_3$) δ 8.00 (t, *J* = 1.7 Hz, 1H), 7.96 – 7.83 (m, 2H), 7.60 (ddd, *J* = 8.0, 2.1, 1.1 Hz, 1H), 7.54 (d, *J* = 8.3 Hz, 2H), 7.44 (t, *J* = 7.8 Hz, 1H), 7.23 (d, *J* = 8.0 Hz, 2H), 7.17 – 7.07 (m, 1H), 7.05 – 6.93 (m, 1H), 6.88 (dd, *J* = 8.1, 1.4 Hz, 1H), 4.56 – 4.28 (m, 3H), 3.63 (dtd, *J* = 9.6, 4.7, 2.4 Hz, 1H), 3.38 (dd, *J* = 14.4, 10.1 Hz, 1H), 2.40 (s, 3H).

^{13}C NMR (75 MHz, $CDCl_3$) δ 165.1 (s), 155.7 (s), 146.9 (s), 144.9 (s), 135.7 (s), 135.1 (d), 133.9 (s), 131.5 (d), 130.5 (d), 130.3 (d), 130.1 (d), 128.2 (d), 127.6 (d), 126.8 (d), 125.1 (s), 123.8 (d), 121.7 (d), 118.0 (d), 69.7 (d), 64.5 (t), 46.3 (t), 22.0 (q).

MS (ESI): m/z 480.00, 481.98 $[M+Na]^+$

Anal. Calcd for $C_{23}H_{20}ClNO_5S$: C, 60.33; H, 4.40; N, 3.06. Found: C, 60.38; H, 4.38; N, 3.09.

From GP6. *Reaction conditions*: **1a** (0.3 mmol, 1 eq), $Pd(OAc)_2$ 10 mol%, **L4** 12 mol% in CH_3CN (0.4 M) T = -20° C. **3aa** *er* 65:35, 31 mg, 0.07 mmol, 23% yield. HPLC (Chiracel OD-H, l = 225 nm, n-Hex/iPrOH = 70:30, 0.5 mL/min): tR = 10.0 min (minor), 13.6 min (major).

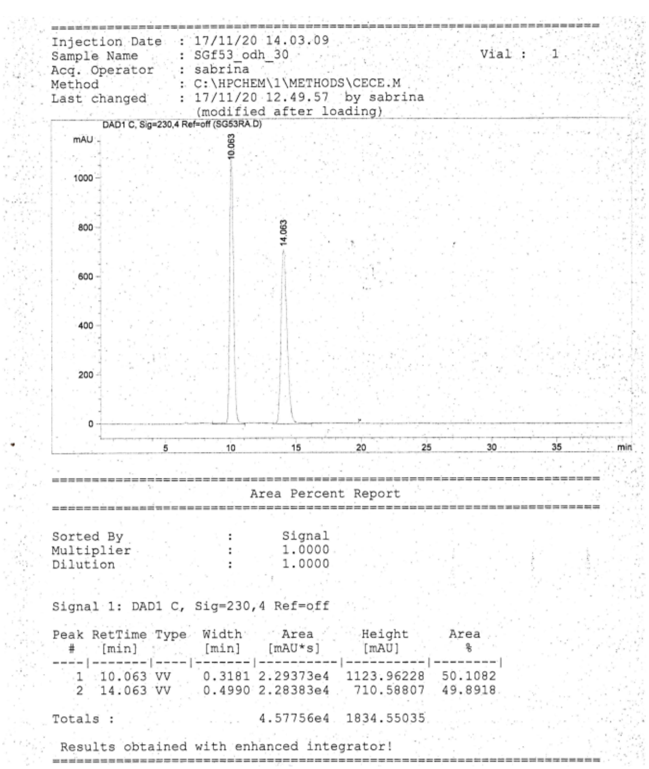

Peak #	RetTime [min]	Type	Width [min]	Area [mAU*s]	Height [mAU]	Area %
1	10.063	VV	0.3181	2.29373e4	1123.96228	50.1082
2	14.063	VV	0.4990	2.28383e4	710.58807	49.8918

Totals : 4.57756e4 1834.55035

Results obtained with enhanced integrator!

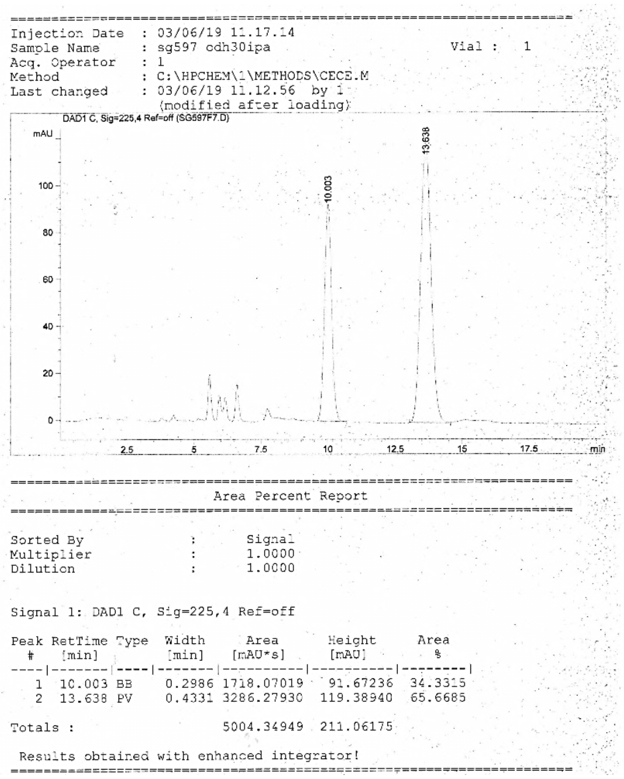

Peak #	RetTime [min]	Type	Width [min]	Area [mAU*s]	Height [mAU]	Area %
1	10.003	BB	0.2986	1718.07019	91.67236	34.3315
2	13.638	PV	0.4331	3286.27930	119.38940	65.6685

Totals : 5004.34949 211.06175

Results obtained with enhanced integrator!

(4-tosyl-3,4-dihydro-2H-benzo[b][1,4]oxazin-2-yl)methyl acetate (3ad)

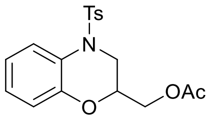

Compound **3ad** was prepared according to the general procedure (GP5, CH_3CN, 24h) and isolated as white solid (23 mg, 0.064 mmol, yield 32%) after flash chromatography (Hex:AcOEt 3:1).

M.p. 95–97° C

IR: 2913 1746, 1597, 1584, 1488 cm^{-1}

^{1}H-NMR (300 MHz, $CDCl_3$) δ: 7.83 (1H, dd, *J* = 8.3, 1.5 Hz), 7.51 (2H, d, *J* = 8.4 Hz,), 7.23 (2H, d, *J* = 8.4 Hz), 7.07 (1H, ddd, *J* = 8.1, 7.3, 1.6 Hz), 6.95 (1H, ddd, *J* = 8.2, 7.3, 1.6 Hz), 6.83 (1H, dd, *J* = 8.1, 1.6 Hz), 4.32 (1H, dd, *J* = 14.4, 2.5 Hz), 4.21 – 4.05 (2H, m), 3.55 (1H, tdd, *J* = 7.2, 4.7, 2.5 Hz), 3.26 (1H, dd, *J* = 14.4, 10.1 Hz), 2.39 (3H, s), 2.08 (3H, s).

^{13}C-NMR (75 MHz, $CDCl_3$) δ: 170.4 (s), 146.5 (s), 144.4 (s), 135.4 (s), 130.0 (d), 127.2 (d), 126.3 (d), 124.5 (d), 123.4 (s), 121.2 (d), 117.5 (d), 69.4 (d), 63.4 (t), 45.7 (t), 21.6 (q), 20.7 (q).

MS (ESI): m/z 362,33 [M+H]$^+$ 384,53 [M+Na]$^+$

Anal. Calcd for $C_{18}H_{19}NO_5S$ C, 59.82; H, 5.30; N, 3.88. Found: C, 59.86; H, 5.27; N, 3.85.

(4-tosyl-3,4-dihydro-2H-benzo[b][1,4]oxazin-2-yl)methyl 2-fluorobenzoate (3ab)

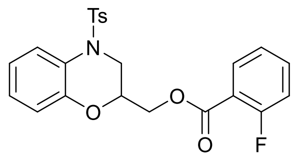

Compound **3ab** was prepared according to the general procedure (GP5, CH_3CN, 3h) and isolated as solid (75 mg, 0.17 mmol, yield 86%) after flash chromatography (Hex/AcOEt 3:1).

R_f = 0.33 (n-Hex/EtOAc 2:1); stained with $KMnO_4$

M.p. 103–105° C

IR = 1721, 1478 cm^{-1}

^1H NMR (300 MHz, $CDCl_3$) δ 7.98 – 7.82 (m, 2H), 7.63 – 7.48 (m, 3H), 7.28 – 7.12 (m, 4H), 7.12 – 7.03 (m, 1H), 7.00 – 6.91 (m, 1H), 6.85 (dd, *J* = 8.2, 1.6 Hz, 1H), 4.53 – 4.24 (m, 3H), 3.74 – 3.47 (m, 1H), 3.36 (dd, *J* = 14.5, 10.1 Hz, 1H), 2.37 (s, *J* = 6.5 Hz, 3H).

^{13}C NMR (75 MHz, $CDCl_3$) δ 163.7 (s), 160.3 (s), 146.5 (s), 144.4 (s), 135.4 (s), [135.1, 135.0] (d), 132.2 (d), 130.0 (d), 127.2 (d), 126.3 (d), 124.7 (d), [124.1, 124.0] (d), 123.5 (s), 121.2 (d), 117.8 (s), 117.5 (d), [117.3, 117.0] (d), 69.2 (s), 64.0 (d), 46.0 (d), 21.5 (q).

MS (ESI): m/z 441.95 $[M+H]^+$

Anal. Calcd for $C_{18}H_{19}NO_5S$ C, 62.58; H, 4.57; N, 3.17. Found: C, 62.61; H, 4.59; N, 3.14.

(4-tosyl-3,4-dihydro-2H-benzo[*b*][1,4]oxazin-2-yl)methyl acetyl-*L*-alaninate (3ac)

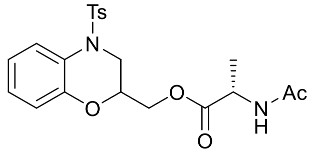

Compound **3ac** was prepared according to the general procedure (GP5, DCM, 40° C) and isolated as a white solid (53 mg, 0.12 mmol, yield 62%) after flash chromatography (Hex/AcOEt 3:1 → 1:1).

R_f = 0.15 (n-Hex/EtOAc 1:2); stained with ninhydrin

M.p. 215–222° C (decomp.)

IR = 1732, 1349, 1160 cm^{-1}

^1H NMR (300 MHz, $CDCl_3$) δ 7.81 (ddd, *J* = 8.2, 5.1, 1.5 Hz, 1H), 7.55 (dd, *J* = 8.3, 1.8 Hz, 2H), 7.27 (d, *J* = 8.5 Hz, 2H), 7.14 – 7.03 (m, 1H), 7.03 – 6.89 (m, 1H), 6.84 (dd, *J* = 8.1, 1.4 Hz, 1H), 6.01 (d, *J* = 5.9 Hz, 1H), 4.82 – 4.48 (m, 1H), 4.35 – 3.99 (m, 3H), 3.83 – 3.51 (m, 1H), 3.32 (ddd, *J* = 14.3, 9.8, 7.8 Hz, 1H), 2.41 (s, *J* = 15.0 Hz, 3H), 2.04 (s, *J* = 7.2 Hz, 3H), 1.42 (dd, *J* = 7.2, 4.0 Hz, 3H).

^{13}C NMR (75 MHz, $CDCl_3$) δ [172.7, 172.6] (s), 169.6 (s), [146.4, 146.3] (s), [144.5, 144.5] (s), [135.6, 135.5] (s), 130.1 (d), 127.2 (d), 126.3 (d), 124.3 (d), 123.5 (s), [121.3, 121.3] (d), [117.5, 117.5] (d), [69.5, 69.4] (d), [64.1, 63.9] (t), 48.0 (d), [45.6, 45.6] (t), 23.1 (q), 21.6 (q), 18.41 (q).

MS (ESI): m/z 433.26 $[M+Na]^+$

Anal. Calcd for $C_{21}H_{24}N_2O_6S$ C, 58.32; H, 5.59; N, 6.48. Found: C, 58.37; H, 5.53; N, 6.50.

***tert*-butyl 2-(((3-chlorobenzoyl)oxy)methyl)-2,3-dihydro-4H-benzo[b][1,4]oxazine-4-carboxylate (3ba)**

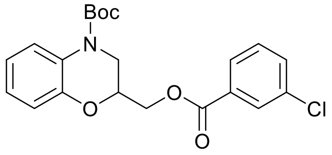

Compound **3ba** was prepared according to the general procedure (GP5, CH_3CN, 24h) and isolated as a colourless oil (41 mg, 0.10 mmol, yield 52%) after flash chromatography (Hex/AcOEt 3:1).

R_f = 0.48 (n-Hex/EtOAc 2:1)

^1H NMR (300 MHz, $CDCl_3$) δ 8.00 (t, J = 1.7 Hz, 1H), 7.92 (d, J = 7.8 Hz, 1H), 7.82 – 7.66 (m, 1H), 7.59 – 7.49 (m, 1H), 7.39 (t, J = 7.9 Hz, 1H), 7.05 – 6.98 (m, 1H), 6.96 – 6.83 (m, 2H), 4.59 – 4.43 (m, 3H), 4.29- 4.13 (m, 1H), 3.69 – 3.51 (m, 1H), 1.52 (s, J = 8.6 Hz, 9H).

MS (ESI): m/z 427.09 $[M+Na]^+$

Anal. Calcd for $C_{21}H_{22}ClNO_5$ C, 62.45; H, 5.49; N, 3.47. Found: C, 62.49; H, 5.47; N, 3.49.

(6-methyl-4-tosyl-3,4-dihydro-2H-benzo[b][1,4]oxazin-2-yl)methyl 2-fluorobenzoate (3cb)

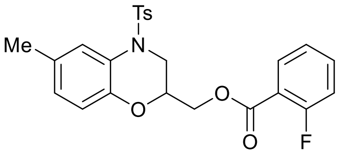

Compound **3cb** was prepared according to the general procedure (GP5, CH_3CN, 4h) and isolated as white solid (75 mg, 0.16 mmol, yield 84%) after flash chromatography (Hex/AcOEt 5:1).

R_f = 0.2 (n-Hex/EtOAc 3:1); stained with $KMnO_4$

M.p. 111–113° C

^1H NMR (300 MHz, $CDCl_3$) δ 7.92 (td, J = 7.6, 1.9 Hz, 1H), 7.68 (d, J = 1.7 Hz, 1H), 7.63 – 7.46 (m, 3H), 7.28 – 7.12 (m, 4H), 6.96 – 6.82 (m, 1H), 6.73 (d, J = 8.3 Hz, 1H), 4.50 – 4.21 (m, 3H), 3.58 – 3.46 (m, 1H), 3.32 (dd, J = 14.4, 10.2 Hz, 1H), 2.37 (s, J = 6.8 Hz, 3H), 2.32 (s, J = 14.8 Hz, 3H).

^{13}C NMR (75 MHz, $CDCl_3$) δ 163.7 (s), 160.3 (s), [144.4, 144.3] (s), 135.5 (s), [135.0, 134.9] (d), 132.2 (d), 130.6 (s), 129.9 (d), 127.2 (d), 127.1 (d), 124.9 (d), [124.1, 124.1] (d), 123.1 (s), 117.1 (d), [117.1, 116.9] (d), 69.1 (d), 64.0 (t), 46.1 (t), 21.5 (q), 20.8 (q).

MS (ESI): m/z 456.14 [M+H]$^{+,}$ 478.33 [M+Na]$^{+}$

Anal. Calcd for $C_{24}H_{22}FNO_5S$ C, 63.29; H, 4.87; N, 3.08. Found: C, 63.25; H, 4.89; N, 3.11.

(7-methyl-4-tosyl-3,4-dihydro-2H-benzo[b][1,4]oxazin-2-yl)methyl 2-fluorobenzoate (3db)

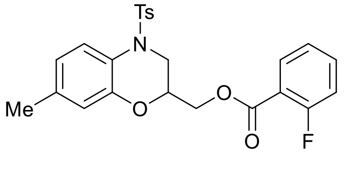

Compound **3db** was prepared according to the general procedure (GP5, CH_3CN, 3h) and isolated as pale-yellow oil (72 mg, 0.16 mmol, yield 81%) after flash chromatography (Hex/AcOEt 4:1 → 2:1).

R_f = 0.11 (n-Hex/EtOAc 3:1); stained with $KMnO_4$

^{1}H NMR (300 MHz, $CDCl_3$) δ 8.00 – 7.82 (m, 1H), 7.74 (d, J = 8.4 Hz, 1H), 7.63 – 7.39 (m, 3H), 7.20 (dddd, J = 13.8, 10.9, 8.0, 1.0 Hz, 4H), 6.78 (ddd, J = 8.4, 2.0, 0.6 Hz, 1H), 6.66 (dd, J = 1.8, 0.6 Hz, 1H), 4.52 – 4.19 (m, 3H), 3.53 (dddd, J = 10.0, 7.9, 4.3, 2.3 Hz, 1H), 3.32 (dd, J = 14.5, 10.2 Hz, 1H), 2.37 (s, 3H), 2.27 (s, J = 7.6 Hz, 3H).

^{13}C NMR (75 MHz, $CDCl_3$) δ 163.7 (s), 146.3 (s), 144.2 (s), 136.5 (s), 135.4 (s), [135.0, 134.9] (d), 132.2 (s), 129.9 (s), 127.3 (s), 124.6, [124.1, 124.0] (d), 122.1 (s), 120.9 (s), 117.7 (s), [117.2, 117.0] (d), 69.1 (d), 64.0 (t), 46.1 (t), 21.5 (q), 20.8 (q).

MS (ESI): m/z 456.02 [M+H]$^{+,}$ 473.18 [M+Na]$^{+}$

Anal. Calcd for $C_{24}H_{22}FNO_5S$ C, 63.29; H, 4.87; N, 3.08. Found: C, 63.24; H, 4.88; N, 3.12.

(6-methoxy-4-tosyl-3,4-dihydro-2H-benzo[*b*][1,4]oxazin-2-yl)methyl 2-fluorobenzoate (3eb)

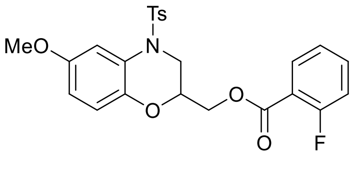

Compound **3eb** was prepared according to the general procedure (GP5, CH_3CN, 5h) and isolated as pale-yellow oil (68 mg, 0.14 mmol, yield 73%) after flash chromatography (Hex/AcOEt 4:1 → 2:1).

R_f = 0.17 (n-Hex/EtOAc 3:1); stained with $KMnO_4$

^{1}H NMR (300 MHz, CDCl3) δ 7.93 (td, J = 7.6, 1.8 Hz, 1H), 7.59 – 7.44 (m, 3H), 7.30 – 7.05 (m, 4H), 6.82 – 6.61 (m, 2H), 4.51 – 4.20 (m, 3H), 3.81 (s, J = 6.9 Hz, 3H), 3.52 (ddd, J = 9.9, 7.1, 2.2 Hz, 1H), 3.34 (dd, J = 14.3, 10.1 Hz, 1H), 2.37 (s, J = 20.0 Hz, 3H).

^{13}C NMR (75 MHz, $CDCl_3$) δ 163.7, 160.3, 153.8, 148.1, 144.4, 140.5, 135.3, 135.0, 134.9, 132.2, 130.0, 129.4, 127.6, 127.2, 124.1, 124.1, 123.7, 117.9, 117.3, 117.0, 113.3, 108.8, 69.0, 64.0, 55.8, 46.3, 21.5.

MS (ESI): m/z 471.52 $[M+H]^+$

Anal. Calcd for $C_{24}H_{22}FNO_6S$ C, 61.14; H, 4.70; N, 2.97. Found: C, 61.09; H, 4.74; N, 2.99.

(4-tosyl-3,4-dihydro-2H-naphtho[2,3-b][1,4]oxazin-2-yl)methyl 2-fluorobenzoate (3fb)

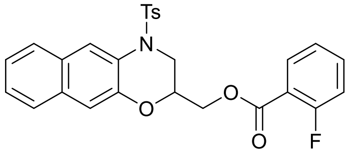

Compound **3fb** was prepared according to the general procedure (GP5, CH_3CN, 3h) and isolated as white solid (77 mg, 0.15 mmol, yield 79%) after flash chromatography (Hex/AcOEt 4:1 → 2:1).

R_f = 0.14 (n-Hex/EtOAc 4:1); stained with $KMnO_4$

M.p. 99–101° C

^1H NMR (300 MHz, $CDCl_3$) δ 8.39 (s, 1H), 7.97 (td, *J* = 7.6, 1.9 Hz, 1H), 7.87 – 7.75 (m, 1H), 7.62 (dd, *J* = 24.3, 7.7 Hz, 3H), 7.50 – 7.32 (m, 2H), 7.28 – 7.08 (m, 4H), 4.72 – 4.31 (m, 3H), 3.84 (tdd, *J* = 10.4, 6.6, 4.0 Hz, 1H), 3.55 (dd, *J* = 14.3, 10.2 Hz, 1H), 2.37 (s, 3H).

^{13}C NMR (75 MHz, $CDCl_3$) δ [156.9, 156.8] (s), 155.4 (s), 144.9 (s), 136.0 (s), [135.5, 135.4] (d), 132.7 (d), 132.2 (s), 130.4 (d), 128.3 (d), 127.6 (d), [126.5, 126.5] (d), 124.8 (d), 124.8 (d), 124.5 (d), 122.5 (d), [117.7, 117.4] (d), 113.1 (d), 70.1 (d), 64.4 (t), 46.7 (t), 21.9 (q).

MS (ESI): m/z 492.12 $[M+H]^{+,}$ 514.30 $[M+Na]^+$

Anal. Calcd for $C_{27}H_{22}FNO_5S$ C, 65.98; H, 4.51; N, 2.85. Found: C C, 65.95; H, 4.53; N, 2.89.

(1-tosyl-1,2,3,5-tetrahydrobenzo[e][1,4]oxazepin-3-yl)methyl 3-chlorobenzoate (4aa)

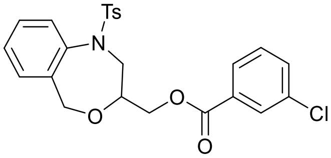

Compound **4aa** was prepared according to the general procedure (GP5, CH_3CN, 40° C) and isolated as brownish gum (53 mg, 0.12 mmol, yield 59%) after flash chromatography (Hex:AcOEt 5:1 → 3:1).

R_f = 0.31 (n-Hex/EtOAc 2:1); stained with $KMnO_4$

^1H NMR (300 MHz, $CDCl_3$) δ 8.06 – 7.99 (m, 1H), 7.98 – 7.90 (m, 1H), 7.65 (d, *J* = 8.3 Hz, 2H), 7.57 (ddd, *J* = 8.0, 2.1, 1.1 Hz, 1H), 7.47 – 7.17 (m, 7H), 4.54 (dd, *J* = 16.7, 14.3 Hz, 2H), 4.45 – 4.22 (m, 3H), 4.14 – 3.96 (m, 1H), 3.17 (dd, *J* = 15.0, 10.3 Hz, 1H), 2.45 (s, *3*H).

^{13}C NMR (75 MHz, $CDCl_3$) δ 165.0 (s), 143.9 (s), 139.7 (s), 138.2 (s), 137.9 (s), 134.6 (s), 133.3 (d), 131.4 (s), 129.9 (d), 129.8 (d), 129.8 (d), 129.0 (d), 128.9 (d), 128.1 (d), 127.9 (d), 127.2 (d), 78.1 (d), 72.8 (t), 65.3 (t), 53.2 (t), 21.6 (q).

MS (ESI): m/z 472.06 $[M+H]^+$

Anal. Calcd for $C_{24}H_{22}ClNO_5S$ C, 61.08; H, 4.70; N, 2.97. Found: C, 61.12; H, 4.68; N, 2.99.

Synthesis of *N*-allyl-2,2,2-trifluoro-*N*-(2-hydroxyphenyl)acetamide (1i)

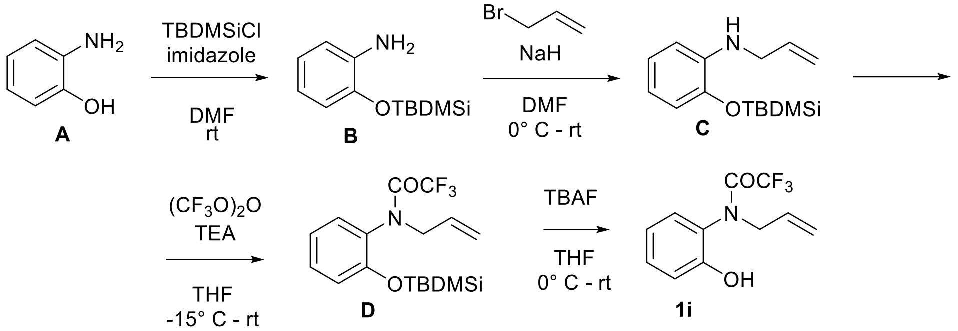

A solution of *2*-aminophenol **A** (2.0 gr, 18.5 mmol, 1 eq), *tert*-butyldimethylsilyl chloride (18.5 mmol, 1 eq) and imidazole (18.5 mmol, 1 eq) in DMF (0.3 M) was stirred at room temperature overnight. The reaction was taken up with brine (50 mL), extracted with Et_2O (3 x 50 mL), dried over Na_2SO_4 and concentrated at reduced pressure. The crude product was purified by silica gel column chromatography gel (n-Hex/EtOAc 10:1) to afford **B** (3.68 gr, 16.5 mmol, 89%).

In a two-neck round-bottom flask **B** (1 gr, 4.48 mmol,1 eq.) was dissolved in a THF/DMF mixture (5:1, 0.2 M) and then K_2CO_3 (4.48 mmol, 1 eq) was added. The reaction mixture was cooled to 0° C and afterwards, a solution of allyl bromide (4.48 mmol, 1. eq) in THF (1 M) was added dropwise over 30 min. Then, the reaction was allowed to warm to room temperature and stirred for 24 h. Once the reaction has occurred as completely as possible, the mixture was filtered *in vacuo*, rinsed with AcOEt and washed with brine. The organic phase was dried over Na_2SO_4 and the solvent evaporated under reduced pressure. The crude product was purified by flash chromatography on silica gel (n-Hex/EtOAc 15:1) to afford **C** (814 mg, 3.08 mmol, 69%).

To a two-neck round-bottom flask **C** (814 mg, 3.08 mmol, 1 eq) was added and dissolved in THF (13 mL, 1 M). After cooling to -15° C, TEA (0.43 mL, 3.08 mmol, 1.0 eq) and trifluoroacetic anyhydride (0.44 mL, 3.08 mmol, 1.0 eq) were added dropwise. The solution was allowed to warm to rt and stirred for 2 h. Afterwards, the solvent was evaporated under reduced pressure and the crude mixture was rinsed with Et_2O (20 mL) and washed with water (20 mL). The solvent was dried over Na_2SO_4, filtered and evaporated *in vacuo*. The crude product was purified by flash chromatography on silica gel (n-Hex/EtOAc 10:1) to afford **D** (814 mg, 1.63 mmol, 53%).

A mixture of the corresponding **D** (590 mg, 1.63 mmol, 1 eq) and tetrabutylammonium fluoride (1.63 mmol, 1 eq) in THF (0.2 M) was stirred at room temperature for 2 h. The solvent was evaporated under reduced pressure, water was added (5 mL) and the reaction mixture was extracted with DCM (3 x 20 mL), then dried over Na_2SO_4, and the mixture concentrated at reduced pressure. The crude product was purified by silica gel column chromatography (Hex/AcOEt 5:1) to afford the **1d** (355 mg, 1.45 mmol, 89%) as a as brown oil.

N-allyl-2,2,2-trifluoro-*N*-(2-hydroxyphenyl)acetamide (1i)

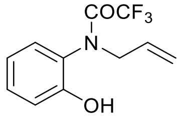

^{1}H NMR (300 MHz, $CDCl_3$) δ 7.39 – 7.23 (m, 1H), 7.09 (d, *J* = 7.8 Hz, 1H), 7.00 – 6.86 (m, 2H), 5.98 – 5.74 (m, 1H), 5.25 – 5.13 (m, 2H), 4.60 (dd, *J* = 14.3, 6.1 Hz, 1H), 3.97 (dd, *J* = 14.3, 7.3 Hz, 1H).

^{13}C NMR (75 MHz, $CDCl_3$) δ 152.2 (s), 136.7 (s), 130.8 (d), 130.7 (d), 130.3 (d), 125.6 (s), 120.6 (d), 120.4 (t), 116.7 (d), 116.1 (s), 53.3 (t).

MS (ESI): m/z 245.98 $[M+H]^+$

Anal. Calcd for $C_{11}H_{10}F_3NO_2$ C, 53.88; H, 4.11; N, 5.71. Found: C, 53.85; H, 4.09; N, 5.70.

Synthesis of *N*-allyl-2,2,2-trifluoro-*N*-(2-hydroxyphenyl)acetamide (1j)

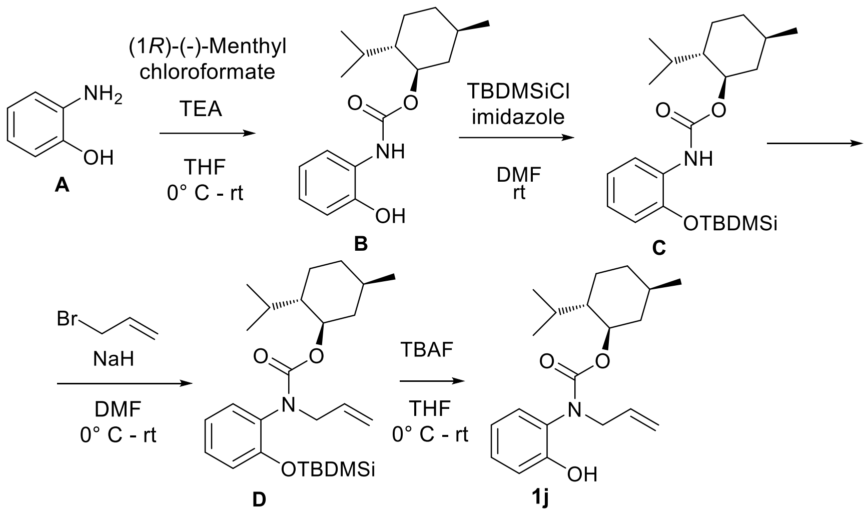

To a two-neck round-bottom flask 2-aminophenol **A** (1 gr, 9.16 mmol, 1 eq) was added and dissolved in THF (30 mL, 0.3 M). After cooling to 0° C, TEA (2.55 mL, 18.33 mmol, 2.0 eq) was added, followed by (1*R*)-(−)-menthyl chloroformate (3.8 mL, 18.33 mmol, 2.0 eq) The solution was allowed to warm to rt and stirred for 16 h. Afterwards, the solvent was evaporated under reduced pressure, the crude mixture rinsed with AcOEt (30 mL) and washed with saturated solution of NH_4Cl (2 x 20 mL) and brine (20 mL). The organic phases were dried over Na_2SO_4, filtered and the solvent evaporated under reduced pressure. The crude product was purified by silica gel column chromatography (Hex/AcOEt 10:1) to afford **A** (19.19 gr, 6.6 mmol, 72%).

A solution of the *N*-protected aminophenol **B** (19.19 gr, 6.6 mmol, 1 eq), *tert*-butyldimethylsilyl chloride (6.6 mmol, 1 eq) and imidazole (6.6 mmol, 1 eq) in DMF (0.2 M) was stirred at room temperature overnight. The reaction was taken with brine (50 mL), extracted with Et_2O (3 x 50 mL), dried over Na_2SO_4 and concentrated at reduced pressure. The crude product was purified by silica gel column chromatography gel (Hex/EtOAc 20:1) to afford **C** (2.93 gr, 5.02 mmol, 76%).

To a mixture of sodium hydride (7.53 mmol, 1.5 eq) in THF (15 mL) at 0 °C under argon atmosphere, a solution of **C** (2.93 gr, 5.02 mmol, 1 eq) in THF (3 mL) was dropped. The reaction mixture was stirred for 30 minutes at room temperature, then cooled at 0 °C. A solution of allyl bromide (5.2 mmol, 1.05 eq) in THF (3 mL) was dropped and the mixture was stirred at room temperature overnight. The mixture was quenched with water (10 mL) and concentrated at reduced pressure. Then, the crude mixture was rinsed with EtOAc (3 x 40 mL), washed with brine (3 x 20 mL), dried on Na_2SO_4, filtrated and concentrated at reduced pressure. The crude product was purified by silica gel column chromatography (Hex/AcOEt 20:1) to afford **D** (1.47 gr, 3.21 mmol, 64%).

A mixture of the corresponding **D** (1.47 gr, 3.21 mmol, 1 eq) and tetrabutylammonium fluoride (3.21 mmol, 1 eq) in THF (0.2 M) was stirred at room temperature for 3 h. The solvent was evaporated under reduced pressure, water was added (10 mL) and the reaction mixture was extracted with DCM (3 x 20 mL), then dried over Na_2SO_4, and the mixture concentrated at reduced pressure. The crude product was purified by silica gel column chromatography (Hex/AcOEt 4:1) to afford the **1j** (997 mg, 2.95 mmol, 92%) as a colourless oil.

***(1R,2S,5R)*-2-isopropyl-5-methylcyclohexyl allyl(2-hydroxyphenyl)carbamate (1j)**

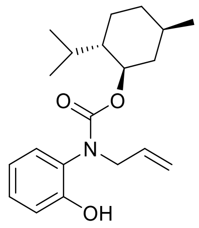

R_f = 0.49 (n-Hex/EtOAc 2:1); stained with $KMnO_4$

^{1}H NMR (300 MHz, $CDCl_3$) δ 7.17 (t, J = 7.5 Hz, 2H), 7.01 (d, J = 7.9 Hz, 1H), 6.90 (t, J = 7.6 Hz, 1H), 5.91 (ddd, J = 16.0, 10.8, 5.7 Hz, 1H), 5.32 – 5.06 (m, 2H), 4.68 (br s, 1H), 4.24 (d, J = 4.9 Hz, 2H), 2.09 (d, J = 11.7 Hz, 1H), 2.02 – 1.19 (m, 8H), 0.99 – 0.67 (m, 11H).

^{13}C NMR (75 MHz, CDC_3) δ 155.9 (s), 151.4 (s), 133.5 (d), 128.2 (d), 121.0 (d), 117.4 (s), 76.9 (d), 53.5 (t), 47.2 (d), 41.2 (t), 34.2 (t), 31.4 (d), 26.2 (d), 23.4 (t), 22.0 (d), 20.7 (q), 17.7 (d), 16.3 (q), 12.3 (q).

MS (ESI): m/z 332.10 $[M+H]^+$

Anal. Calcd for $C_{20}H_{29}NO_3$ C, 72.47; H, 8.82; N, 4.23. Found: C, 72.49; H, 8.80; N, 4.20.

7-oxo-1-tosyl-1,2,3,3a,6,7-hexahydro-7aH-3,6-methanoindol-7a-yl 3-chlorobenzoate (5aa)

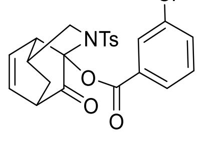

Compound **5aa** was prepared according to the general procedure (GP7) and isolated as white solid (97 mg, 0.21 mmol, yield 71%) after flash chromatography (Hex/AcOEt 4:1).

R_f = 0.32 (n-Hex/EtOAc 1:1); stained with $KMnO_4$

M.p.: 100–102° C

IR = 2935, 1744, 1341, 1151, 665 cm^{-1}

^{1}H NMR (500 MHz, $CDCl_3$) δ 7.95 – 7.78 (m, 2H), 7.69 (d, *J* = 8.3 Hz, 2H), 7.59 – 7.47 (m, 1H), 7.34 (t, *J* = 8.1 Hz, 1H), 7.14 (d, *J* = 8.2 Hz, 2H), 6.41 (td, *J* = 7.6, 1.8 Hz, 1H), 6.21 – 6.02 (m, 1H), 4.28 (ddd, *J* = 6.2, 4.1, 1.9 Hz, 1H), 3.92 (dd, *J* = 9.5, 3.2 Hz, 1H), 3.64 (d, *J* = 9.5 Hz, 1H), 3.20 – 3.15 (m, 1H), 2.52 – 2.38 (m, 1H), 2.29 (s, 3H), 1.85 – 1.70 (m, 1H), 1.61 – 1.44 (m, 1H).

^{13}C NMR (126 MHz, $CDCl_3$) δ 195.7 (s), 163.3 (s), 143.9 (s), 136.3 (s), 134.5 (s), 133.5 (d), 132.7 (d), 131.6 (s), 130.1 (d), 129.6 (d), 129.4 (d), 129.4 (d), 128.4 (d), 128.2 (d), 89.2 (d), 54.7 (t), 46.3 (d), 45.7 (d), 33.2 (d), 28.5 (t), 21.6 (q).

HR-MS (pos. APCI) m/z: $[M+H]^+$ Calcd for $C_{23}H_{20}NO_5ClS$ 458.0751; Found: 458.0823

7-oxo-1-tosyl-1,2,3,3a,6,7-hexahydro-7aH-3,6-methanoindol-7a-yl acetate (5ad)

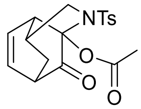

Compound **5ad** was prepared according to the general procedure (GP7) and isolated as colourless oil (25 mg, 0.07 mmol, yield 23%) after flash chromatography (Hex/AcOEt 1:1).

^{1}H NMR (300 MHz, $CDCl_3$) δ 7.75 (d, *J* = 8.3 Hz, 2H), 7.31 (d, *J* = 8.1 Hz, 2H), 6.48 – 6.28 (m, 1H), 6.12 (ddd, *J* = 7.7, 6.4, 1.1 Hz, 1H), 4.15 (ddd, *J* = 6.2, 4.2, 1.9 Hz, 1H), 3.85 (dd, *J* = 9.3, 3.2 Hz, 1H), 3.46 (d, *J* = 9.3 Hz, 1H), 3.11 – 3.06 (m, 1H), 2.50 – 2.24 (m, 4H), 2.26 – 1.88 (m, 4H), 1.83 – 1.59 (m, 1H).

^{13}C NMR (75 MHz, CDC_3) δ 195.4 (s), 169.3 (s), 143.8 (s), 132.6 (s), 132.2 (d), 129.4 (d), 127.9 (d), 54.6 (t), 45.7 (s), 45.4 (s), 32.6 (s), 28.6 (t), 22.0 (q), 21.6 (q).

MS: (ESI) *m/z* 361.84 $[M+H]^+$

Anal. Calcd for $C_{18}H_{19}NO_5S$ C, 59.82; H, 5.30; N, 3.88. Found: C, 59.87; H, 5.28; N, 3.86.

7-oxo-1-tosyl-1,2,3,3a,6,7-hexahydro-7aH-3,6-methanoindol-7a-yl 2-fluorobenzoate (5ab)

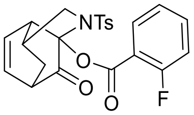

Compound **5ab** was prepared according to the general procedure (GP7) and isolated as white solid (88 mg, 0.22 mmol, yield 67%) after flash chromatography (Hex/AcOEt 3:1).

R_f = 0.36 (n-Hex/EtOAc 1:1); stained with $KMnO_4$

M.p. 102–104° C

IR = 2922, 1735, 1356, 1245, 1168, 1083, 662 cm^{-1}

^{1}H NMR (300 MHz, $CDCl_3$) δ 8.01 (td, *J* = 7.6, 1.8 Hz, 1H), 7.73 (d, *J* = 8.3 Hz, 2H), 7.61 – 7.47 (m, 1H), 7.15 (dddd, *J* = 10.9, 9.2, 6.6, 0.9 Hz, 4H), 6.53 – 6.36 (m, 1H), 6.23 – 6.09 (m, 1H), 4.33 (ddd, *J* = 6.1, 4.1, 1.9 Hz, 1H), 3.92 (dd, *J* = 9.4, 3.1 Hz, 1H), 3.60 (d, *J* = 9.4 Hz, 1H), 3.21 – 3.13 (m, 1H), 2.52 – 2.25 (m, 4H), 1.92 – 1.67 (m, 1H), 1.57 – 1.42 (m, 1H).

^{13}C NMR (75 MHz, $CDCl_3$) δ 195.5 (s), 164.2 (s), 161.2 (s), 160.7 (s), 143.8 (s), 136.3 (s), [135.0, 134.9] (d), 132.9 (d), 132.5 (d), 129.4 (d), 129.3 (d), 128.0 (d), [124.0, 123.9] (d), [116.8, 116.6] (d), 54.6 (t), 46.1 (d), 45.6 (d), 33.0 (d), 28.43 (t), 21.5 (q).

MS: (ESI) *m/z* 441.95 $[M+H]^+$, 465.14 $[M+Na]^+$

Anal. Calcd for $C_{23}H_{20}FNO_5S$ C, 62.58; H, 4.57; N, 3.17. Found: C, 62.54; H, 4.58; N, 3.19.

7-oxo-1-tosyl-1,2,3,3a,6,7-hexahydro-7aH-3,6-methanoindol-7a-yl acetyl-*L*-alaninate (5ac)

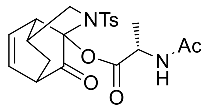

Compound **5ac** was prepared according to the general procedure (GP7) and isolated as a white solid (diastereosiomeric mixture) (75 mg, 0.16 mmol, yield 58%) after flash chromatography (Hex/AcOEt 2:1 → 1:1).

R_f = 0.29 (n-Hex/EtOAc 1:1)

M.p. 84–86° C

IR = 3331, 2919, 1721, 1161, 669, 659 cm^{-1}

^{1}H NMR (300 MHz, $CDCl_3$) δ 7.72 (dd, *J* = 8.4, 2.6 Hz, 2H), 7.36 – 7.29 (m, 2H), 6.49 – 6.26 (m, 2H), 6.25 – 6.01 (m, 1H), 4.60 (pd, *J* = 7.2, 3.6 Hz, 1H), 3.94 (dddd, *J* = 10.6, 6.1, 4.2, 1.8 Hz, 1H), 3.79 (td, *J* = 9.2, 3.2 Hz, 1H), 3.38 (dd, *J* = 19.6, 9.4 Hz, 1H), 3.11 – 2.93 (m, 1H), 2.55 – 2.28 (m, 4H), 2.03 (s, *J* = 4.3 Hz, 3H), 1.78 – 1.59 (m, 1H), 1.50 (dd, *J* = 7.2, 3.7 Hz, 3H), 1.23 – 1.05 (m, 1H).

^{13}C NMR (75 MHz, $CDCl_3$) δ [195.18, 194.91] (s), [170.47, 170.41] (s), [170.16, 170.12] (s), [144.13, 144.05] (s), [136.18, 136.13] (s), [131.47, 131.39] (d), [129.65, 129.61] (d), [129.53, 129.50] (d), [127.80, 127.77] (d), [89.36, 89.33] (s), 54.49 (t), [49.03, 48.94] (d), [46.52, 46.21] (d), 45.38 (d), [32.84, 32.72] (d), [28.58, 28.46] (t), [23.03, 23.00] (q), 21.59 (q), [17.90, 17.76] (q).

MS (ESI): m/z 455.30 $[M+Na]^+$

Anal. Calcd for $C_{21}H_{24}N_2O_6S$ C, 58.32; H, 5.59; N, 6.48. Found: C, 58.37; H, 5.56; N, 6.49.

7a-(1H-benzo[*d*]imidazol-1-yl)-1-tosyl-1,2,3,3a,6,7a-hexahydro-7H-3,6-methanoindol-7-one (5ae)

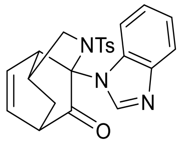

Compound **6ae** was prepared according to the general procedure (GP7, but by using benzimidazole 1.2 eq and $PhI(OAc)_2$ 1.5 eq) and isolated as white solid (78 mg, 0.18 mmol, yield 63%) after flash chromatography (Hex/AcOEt 2:1 → 1:2).

R_f = 0.1 (n-Hex/EtOAc 1:2)

^{1}H NMR (300 MHz, $CDCl_3$) δ 7.93 (s, *J* = 28.7 Hz, 1H), 7.75 (d, *J* = 7.8 Hz, 1H), 7.58 – 7.11 (m, 5H), 7.04 (d, *J* = 8.1 Hz, 2H), 6.47 (t, *J* = 6.8 Hz, 1H), 5.92 (t, *J* = 6.9 Hz, 1H), 4.07 – 3.84 (m, 2H), 3.79 (d, *J* = 10.0 Hz, 1H), 3.51 – 3.23 (m, 1H), 2.62 – 2.45 (m, 1H), 2.32 (s, 3H), 1.94 – 1.73 (m, 1H), 1.62 (d, *J* = 13.6 Hz, 1H).

^{13}C NMR (75 MHz, $CDCl_3$) δ 194.9 (s), 144.2 (s), 143.9 (s), 142.5 (d), 134.6 (s), 132.1 (d), 132.0 (s),129.5 (d), 128.9 (d), 127.9 (d), 123.1 (d), 122.6 (d), 120.6 (d), 112.6 (d), 55.1 (t), 48.8 (d), 46.3 (d), 32.0 (d), 29.0 (t), 21.5 (q).

MS (ESI): m/z 423.24 [M+H]$^+$

Anal. Calcd for $C_{23}H_{21}N_3O_3S$ C, 65.85; H, 5.05; N, 10.02. Found: C, 65.89; H, 5.02; N, 10.04.

(*1R,2S,5R*)-2-isopropyl-5-methylcyclohexyl 7a-((3-chlorobenzoyl)oxy)-7-oxo-2,3,3a,6,7,7a-hexahydro-1H-3,6-methanoindole-1-carboxylate (5ja)

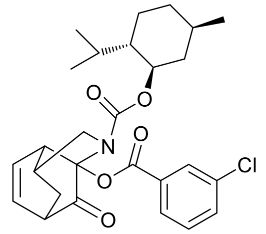

Compound **5ja** was prepared according to the general procedure (GP7) and isolated as light orange solid (66 mg, 0.13 mmol, yield 46%) after flash chromatography (Hex/AcOEt 4:1).

R_f = 0.45 (n-Hex/EtOAc 2:1), stained with $KMnO_4$

M.p. 72–74° C

IR = 2953, 1753, 1731, 1694 cm^{-1}

^{1}H NMR (500 MHz, $CDCl_3$) δ 8.15 – 7.93 (m, 2H), 7.66 – 7.48 (m, 1H), 7.45 – 7.35 (m, 1H), 6.58 – 6.35 (m, 1H), 6.30 – 6.08 (m, 1H), 4.70 – 4.37 (m, 1H), 4.30 – 3.99 (m, 1H), 3.87 (dt, *J* = 10.0, 3.3 Hz, 1H), 3.76 – 3.50 (m, 1H), 3.32 (s, 1H), 2.55 – 2.42 (m, 1H), 2.28 – 0.37 (m, 20H).

^{13}C NMR (126 MHz, $CDCl_3$) δ 194.8, 163.3, 149.6, 133.4, 132.4, 130.1, 129.7, 129.6, 128.9, 75.8, 53.3, 52.8, 46.1, 45.7, 41.0, [34.2, 34.1], 32.0, 31.5, 29.9, 29.7, 22.0, 21.9, 21.0, 15.8, 15.3.

MS: (ESI) *m/z* 485.86 [M+H]$^+$

Anal. Calcd for $C_{27}H_{32}ClNO_5$ C, 66.73; H, 6.64; N, 2.88. Found: C, 66.78; H, 6.66; N, 2.86.

5-methyl-7-oxo-1-tosyl-1,2,3,3a,6,7-hexahydro-7aH-3,6-methanoindol-7a-yl 3-chlorobenzoate (5da)

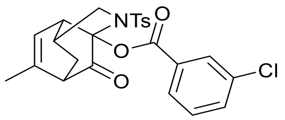

Compound **5da** was prepared according to the general procedure (GP7) and isolated as white solid (74 mg, 0.16 mmol, yield 52%) after flash chromatography (Hex/AcOEt 3:1).

M.p. 94-96° C

IR = 2918, 1731, 1596 cm^{-1}

^{1}H NMR (300 MHz, $CDCl_3$) δ 7.94 – 7.80 (m, 2H), 7.70 (d, *J* = 8.3 Hz, 2H), 7.58 – 7.45 (m, 1H), 7.35 (t, *J* = 8.1 Hz, 1H), 7.15 (d, *J* = 8.1 Hz, 2H), 5.85 – 5.62 (m, 1H), 4.24 (dd, *J* = 6.4, 4.2 Hz, 1H), 3.94 (dd, *J* = 9.5, 3.1 Hz, 1H), 3.65 (d, *J* = 9.5 Hz, 1H), 2.53 – 2.37 (m, 1H), 2.30 (s, *J* = 16.0 Hz, 3H), 1.87 (d, *J* = 1.5 Hz, 3H), 1.86 – 1.69 (m, 1H), 1.56 – 1.46 (m, 1H).

^{13}C NMR (75 MHz, $CDCl_3$) δ 195.2 (s), 163.2 (s), 143.8 (s), 142.35 (s), 136.21 (s), 134.32 (s), 133.32 (d), 131.65 (s), 130.0 (d), 129.5 (d), 129.3 (d), 128.3 (d), 128.1 (d), 121.3 (d), 54.67 (t), 51.0 (d), 45.7 (d), 33.9 (d), 28.1 (t), 21.45 (q), 19.92 (q).

MS (ESI): m/z 494.54 $[M+Na]^+$

Anal. Calcd for $C_{24}H_{22}ClNO_5S$ C, 61.08; H, 4.70; N, 2.97. Found: C, 61.11; H, 4.72; N, 2.94.

5-chloro-7-oxo-1-tosyl-1,2,3,3a,6,7-hexahydro-7aH-3,6-methanoindol-7a-yl 3-chlorobenzoate (5ga)

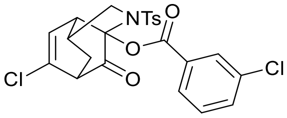

Compound **5ga** was prepared according to the general procedure (GP7) and isolated as light-orange solid (83 mg, 0.17 mmol, yield 57%) after flash chromatography (Hex/AcOEt 4:1 → 2:1).

R_f = 0.44 (n-Hex/EtOAc 1:1); stained with $KMnO_4$

M.p. 177–182° C (decomp.)

^{1}H NMR (300 MHz, $CDCl_3$) δ 7.94 – 7.81 (m, 2H), 7.71 (d, *J* = 8.3 Hz, 2H), 7.55 (d, *J* = 8.0 Hz, 1H), 7.37 (t, *J* = 8.1 Hz, 8H), 7.18 (d, *J* = 8.1 Hz, 2H), 6.32 (dd, *J* = 7.6, 2.6 Hz, 1H), 4.25 (dd, *J* = 3.9, 2.8 Hz, 1H), 3.92 (dd, *J* = 9.6, 3.1 Hz, 6H), 3.67 (d, *J* = 9.5 Hz, 1H), 3.26 (dt, *J* = 7.6, 2.5 Hz, 1H), 2.82 – 2.67 (m, 1H), 2.52 – 2.21 (m, 4H), 1.99 – 1.79 (m, 1H), 1.58 (d, *J* = 13.7 Hz, 1H).

^{13}C NMR (75 MHz, $CDCl_3$) δ 193.4 (s), 163.0 (s), 144.1 (s), 136.0 (s), 134.4 (s), 133.56 (d), 132.1 (s), 131.0 (s), 130.1 (d), 129.6 (d), 129.4 (d), 128.4 (d), 128.1 (d), 126.0 (d), 54.4 (d), 54.0 (t), 46.2 (d), 33.5 (d), 28.7 (t), 21.5 (q).

MS (ESI): m/z 514.27 $[M+Na]^+$

Anal. Calcd for $C_{23}H_{19}Cl_2NO_5S$ C, 56.11; H, 3.89; N, 2.84. Found: C, 56.15; H, 3.86; N, 2.81.

X-Ray Crystallography (Prof. Leonardo Lo Presti)

Single crystal X-ray diffraction analysis of the compound 5aa

Sample specs:

Crystallization method: slow evaporation (42 h) from n–hexane.

Sample description: prism, transparent, with dimensions 0.475 x 0.325 x 0.100 mm.

Mounting: on a glass fibre, with perfluorinated oil.

Comments: The sample shows pleochroism (from colourless to grey) under polarized light. It was polished by mechanical ablation in a drop of perfluorinated oil.

Instrumental specs

Device: Bruker AXS Smart APEX 3-circle diffractometer

Source: normal focus sealed tube

Detector: APEX-II CCD

Experiment temperature: 293(2) K

Cryostat: not used

Wavelength: graphite-monochromated Mo Kα (0.71073 Å).

Data collection extent: full sphere within $\sin\vartheta/\lambda = 0.76$ Å^{-1} (Cu-sphere)

Data collections specs: Detector-to-sample distance: 50 mm,

- ω-scan, $2\vartheta = \omega i = 0$ deg , ω-sweep 180 deg, $\Delta\omega = 0.25$ deg, t /frame = 30 s, 1 run ($\varphi = 0$ deg)
- ω-scan, $2\vartheta = \omega i = -30$ deg , ω-sweep 180 deg, $\Delta\omega = 0.25$ deg, t /frame = 60 s, 4 runs ($\varphi = 0, 90, 180, 270$ deg).

Measured reflections: 17962, 6301 independent (5073 with $I > 2\sigma(I)$)

Rint: 0.0139

Maximum resolution (2ϑ): 61.10 deg

Completeness: 98.9 %

Data reduction programs:

Integration: SAINT+

Reduction: SADABS, XPREP

Structure solution and refinement: Shelxs 2013, Shelxl 2016

Unit cell, lattice and crystal system:

Bravais lattice: Triclinic, primitive

Space group: $P2_1$, number 2

Point group: 2 (C_2)

Laue group: 2/m, number 1

Unit cell (Å, deg, Å^3): a = 8.2924(2), b = 10.0126(2), c = 13.1683(3), α = 87.2660(10), β = 72.5050(10), γ = 85.042(2)., V = 1038.60(4) as estimated from 5406 intense reflections among 5.2 e 55.6 deg of 2ϑ (final integration result).

Formula units in cell (Z): 2

Formula units in the asymmetric unit (Z'): 1

Number of electrons in cell (F_{000}): 476

Computed density: 1.464 g/cm^3

Linear absorption coefficient (μ): 0.322 mm^{-1}

Main statistical results:

Final stats for the spherical atom model (shelxl):

Scale factor: 0.1885(3)

Secondary extinction coefficient: none

$<\Delta/\sigma> = 0.000$

R1(F) = 0.0398 for 5073 Fo > 4σ(Fo), 0.0529 for all the data

wR(F2)= 0.1125 for all the measured data

Goodness-of-fit: 1.014

Flack's parameter: meaningless

$\Delta\rho_{MAX/MIN} = +0.43 / -0.46$ e/Å^3

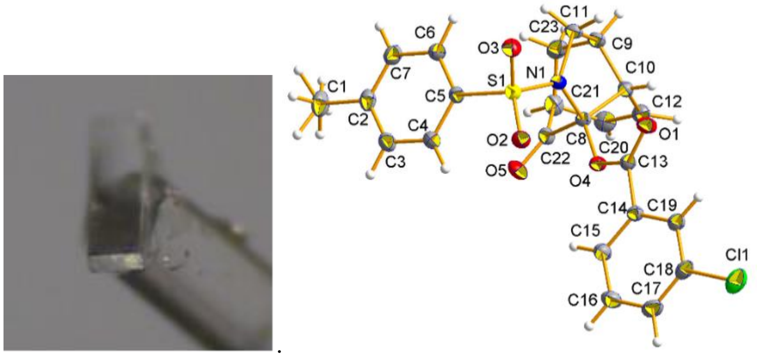

(a) (b)

***Figure S1**. (a) Sample used for the present structural determination. (b) Molecular unit of **5aa** at rt, with the atom-numbering scheme. Thermal ellipsoids of non-H atoms are drawn at the 30 % probability level. The usual colour code was employed for atoms (grey: C; white: H; yellow: S; blue: N; red: O; green: Cl). The terminal methyl group C1 is rotationally disordered across 2 positions with site occupation factors as large as 0.52(7) and 0.48(7).*

The compound is chiral and crystallizes in the centric space group $P\bar{1}$ as a perfect 1:1 racemate, with 1 molecule per asymmetric unit. Figure S1 and S2 show the absolute configuration of the chiral centres at C8 (S), C9 (R), C10 (S) and C21 (S); every unit cell contains also the R, S, R, R enantiomer.

The terminal C1 methyl group is rotationally disordered (Figure *S9*) into two positions with roughly 50% probability of being occupied (site occupation factors: 0.52(7) and 0.48(7)).

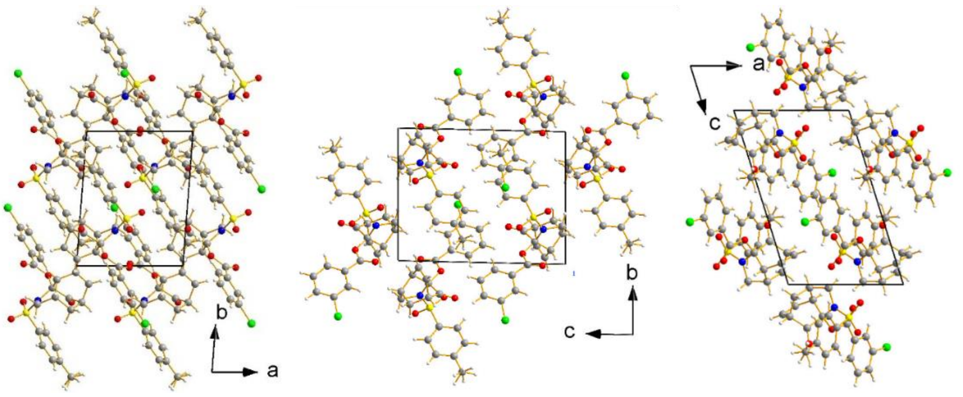

a) b) c)

Figure S2. Ball-and-stick representation of the crystal packing of **5aa** at RT, as seen (a) down the a cell axis; (b) the b cell axis; (c) the c cell axis. Colour code as in Figure S9. The crystallographic reference system is also highlighted.

Figure S9 shows the main packing motifs of **5aa**. The molecule consists of a rather globular aliphatic core, connected with a couple of aromatic rings that are arranged in a ladder-like motif (Figures S1 and S2a) forming stacks that run along the [110] direction (Figure S2c). According with the crystallographic inversion symmetry, C-Cl bonds are oriented antiparallel along the c direction (Figure S2b).

No significant hydrogen bond donors are present in this molecule, which exploits only weak CH···O contacts with both kinds of its symmetry-related images (Table S1). Despite the presence of chlorine, no halogen bonded contacts are found.

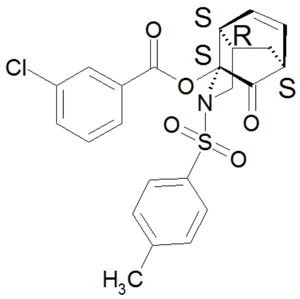

Figure S3. Molecular structure of one enantiomer of **5aa**, with the CIP descriptors highlighted.

Table S1. CH···O intermolecular hydrogen bonded contacts with $d_{H\cdots O} < 3.0$ Å and $120 < \alpha_{CHO} < 180$ deg in **5aa** at room temperature. The disordered C1 group is also likely involved in short contacts with O4 and O5 oxygen atoms (not included). Distances are expressed in Å and angles in degrees. Least–squares estimated standard deviations are given in parentheses

C–H···O	$d_{C–H}$	$d_{H···O}$	$d_{C···O}$	α_{CHO}	Symmetry (CH)
C(11)–H(11A)···O(3)	0.97(2)	2.58(2)	3.518(2)	162(2)	1–x, 1–y, –z
C(6)–H(6)···O(3)	0.99(2)	2.57(2)	3.560(2)	177(2)	1–x, 1–y, –z
C(21)–H(21)···O(2)	0.96(2)	2.96(2)	3.569(2)	123(1)	1+x, y, z
C(20)–H(20)···O(2)	0.95(2)	2.68(2)	3.369(2)	130(2)	1+x, y, z
C(20)–H(20)···O(1)	0.95(2)	2.90(3)	3.719(2)	145(2)	1+x, y, z
C(17)–H(17)···O(2)	0.97(2)	2.53(3)	3.321(3)	139(2)	1–x, –y, 1–z
C(10)–H(10)···O(1)	0.93(1)	2.73(1)	3.458(2)	135(1)	1–x, –y, –z
C(11)–H(11B)···O(1)	1.01(2)	2.62(2)	3.418(2)	137(1)	1–x, –y, –z
C(3)–H(3)···O(5)	0.98(2)	2.60(2)	3.518(2)	156(2)	–x, 1–y, –z

For further information, see crystallographic data, which is available free of charge from the Cambridge Crystallographic Data Centre (www.ccdc.cam.ac.uk/data_request/cif) under the deposition number 2078893.

^{1}H NMR and ^{13}C NMR spectra

^{1}H NMR (300 MHz, $CDCl_3$) of **1a**

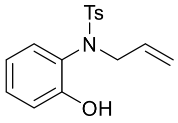

1a

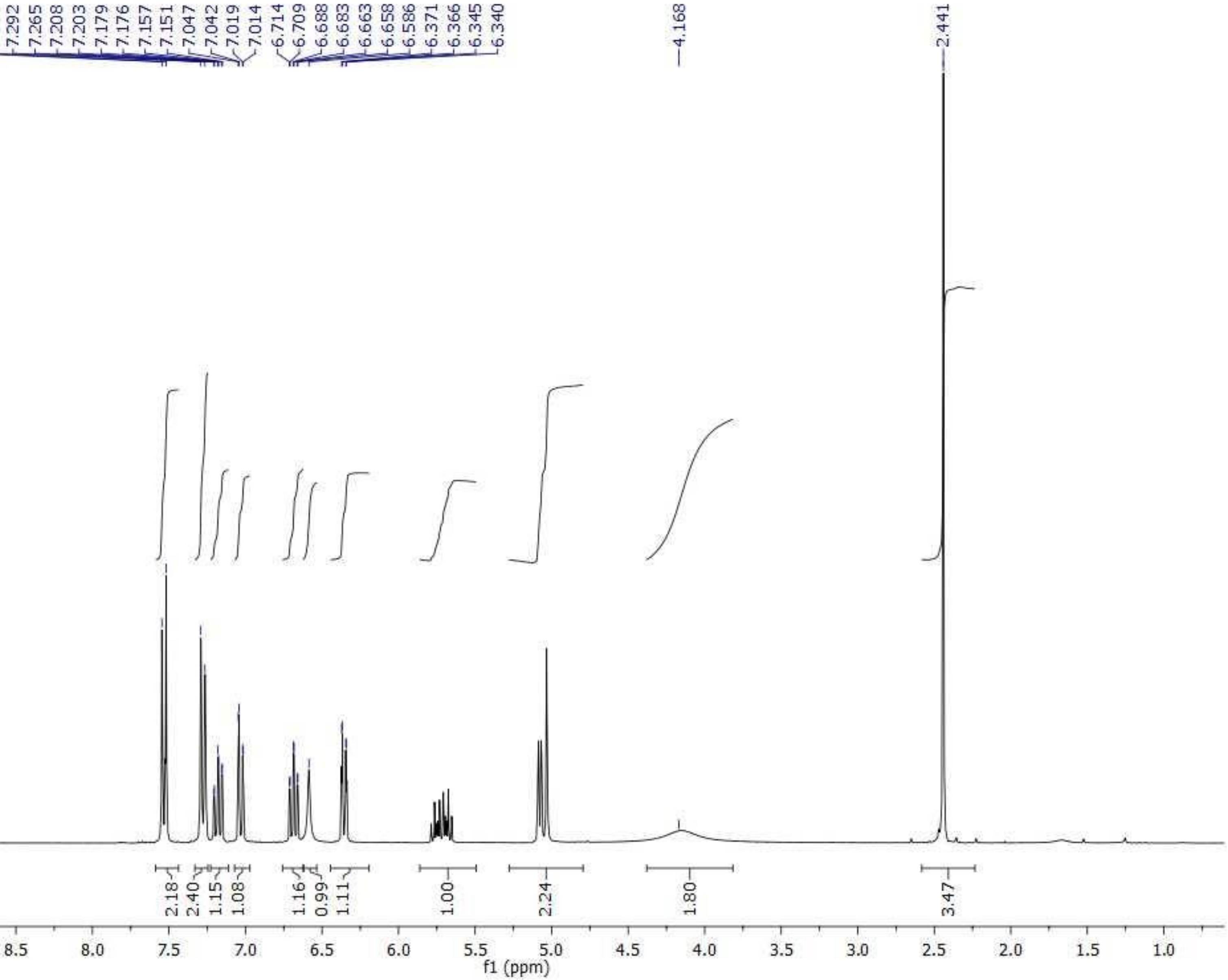

^{13}C NMR (75 MHz, $CDCl_3$) of **1a**

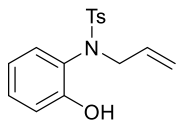

1a

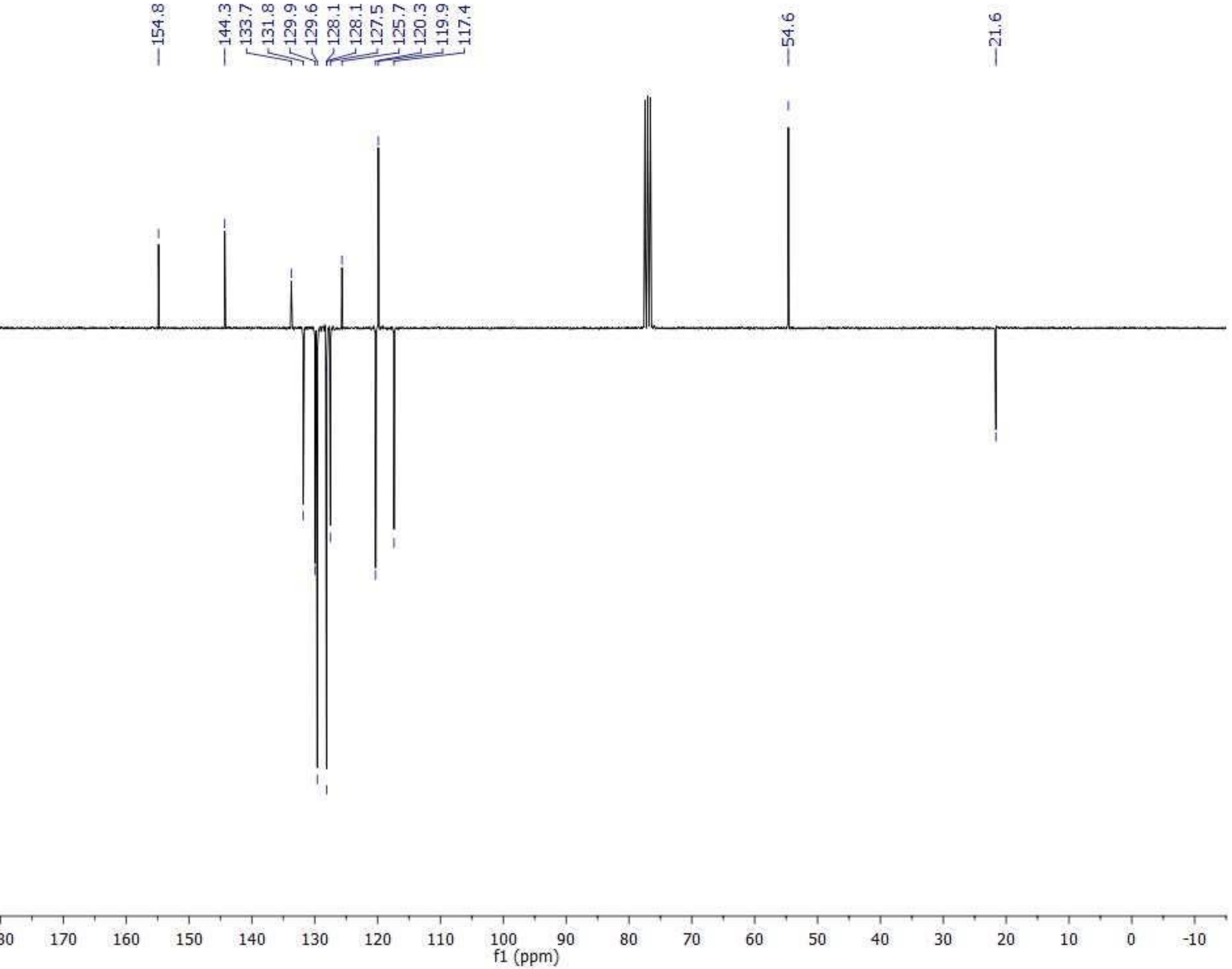

^{1}H NMR (300 MHz, $CDCl_3$) of **1b**

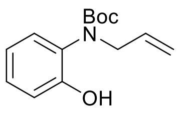

1b

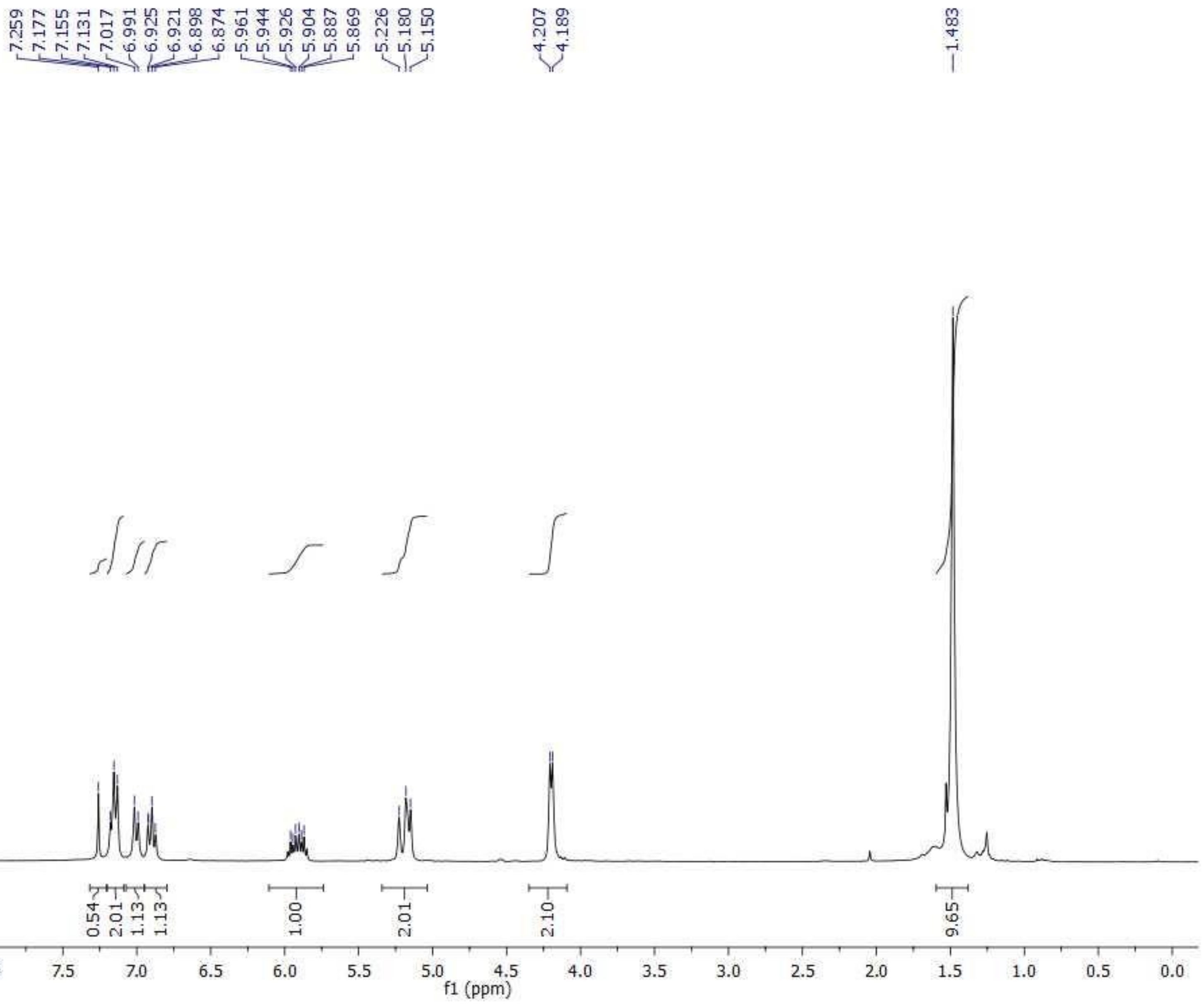

^1H NMR (300 MHz, $CDCl_3$) of **1c**

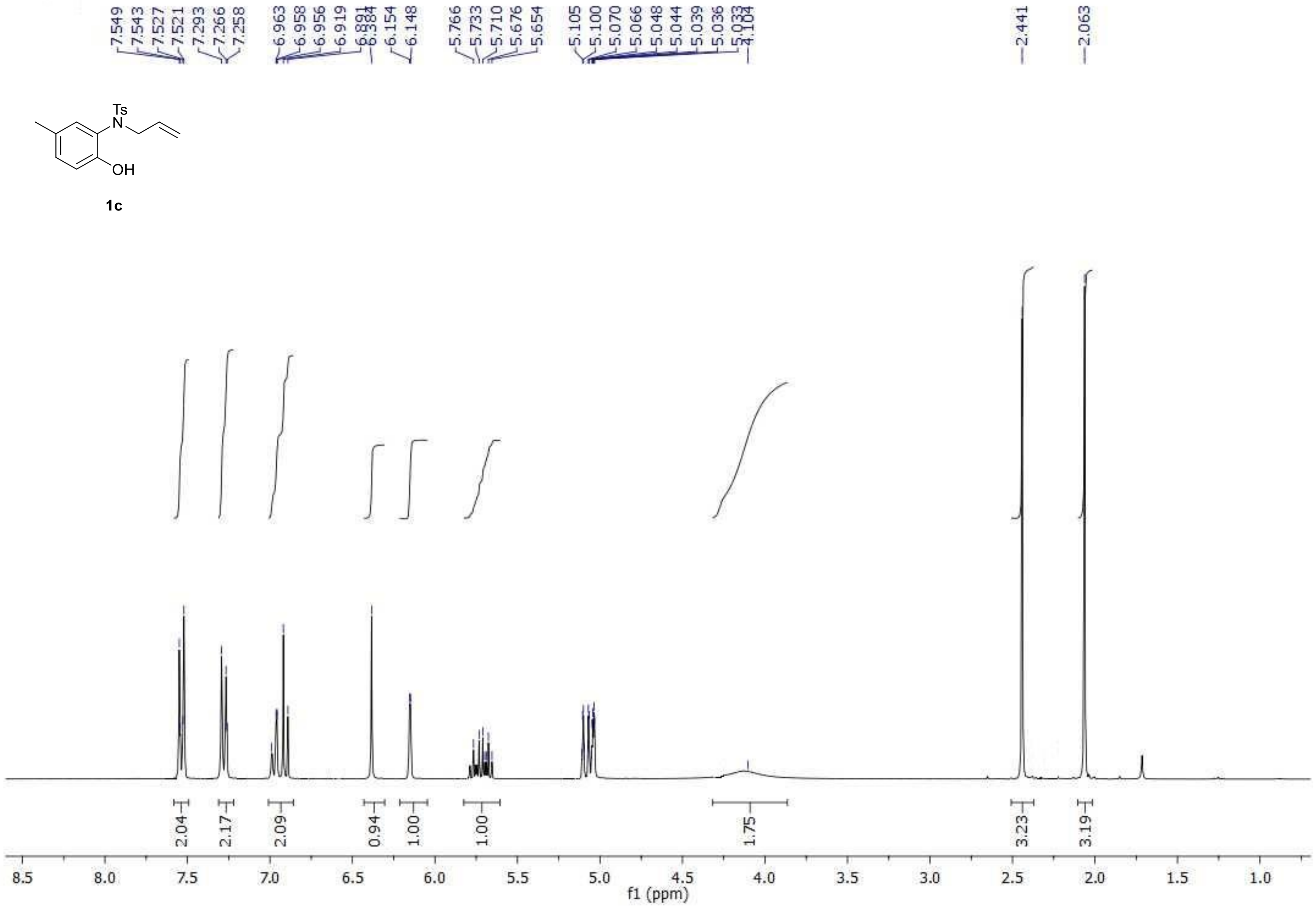

^{13}C NMR (75 MHz, $CDCl_3$) of **1c**

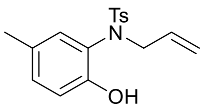

1c

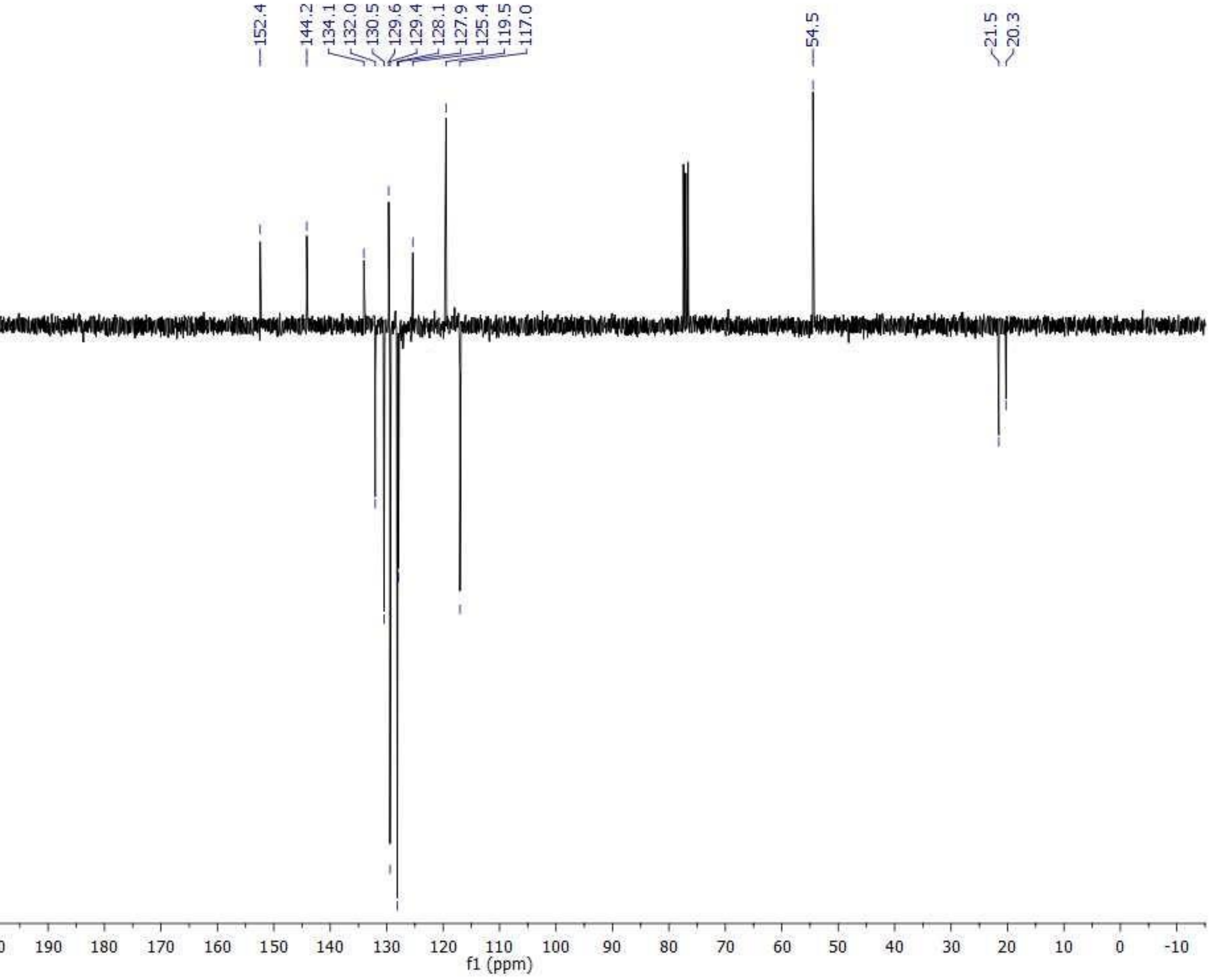

^{1}H NMR (300 MHz, $CDCl_3$) of **1d**

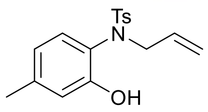

1d

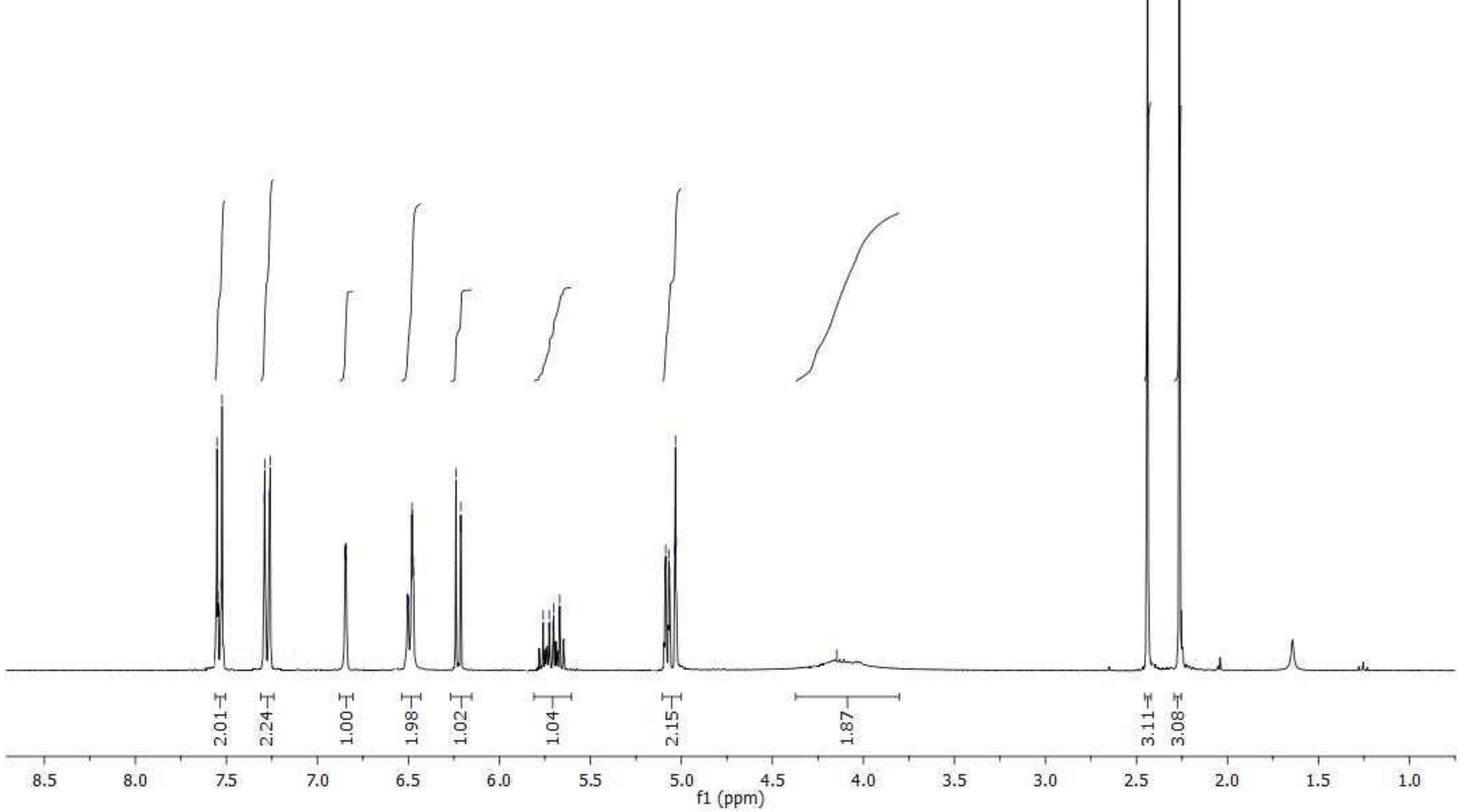

^{13}C NMR (75 MHz, $CDCl_3$) of **1d**

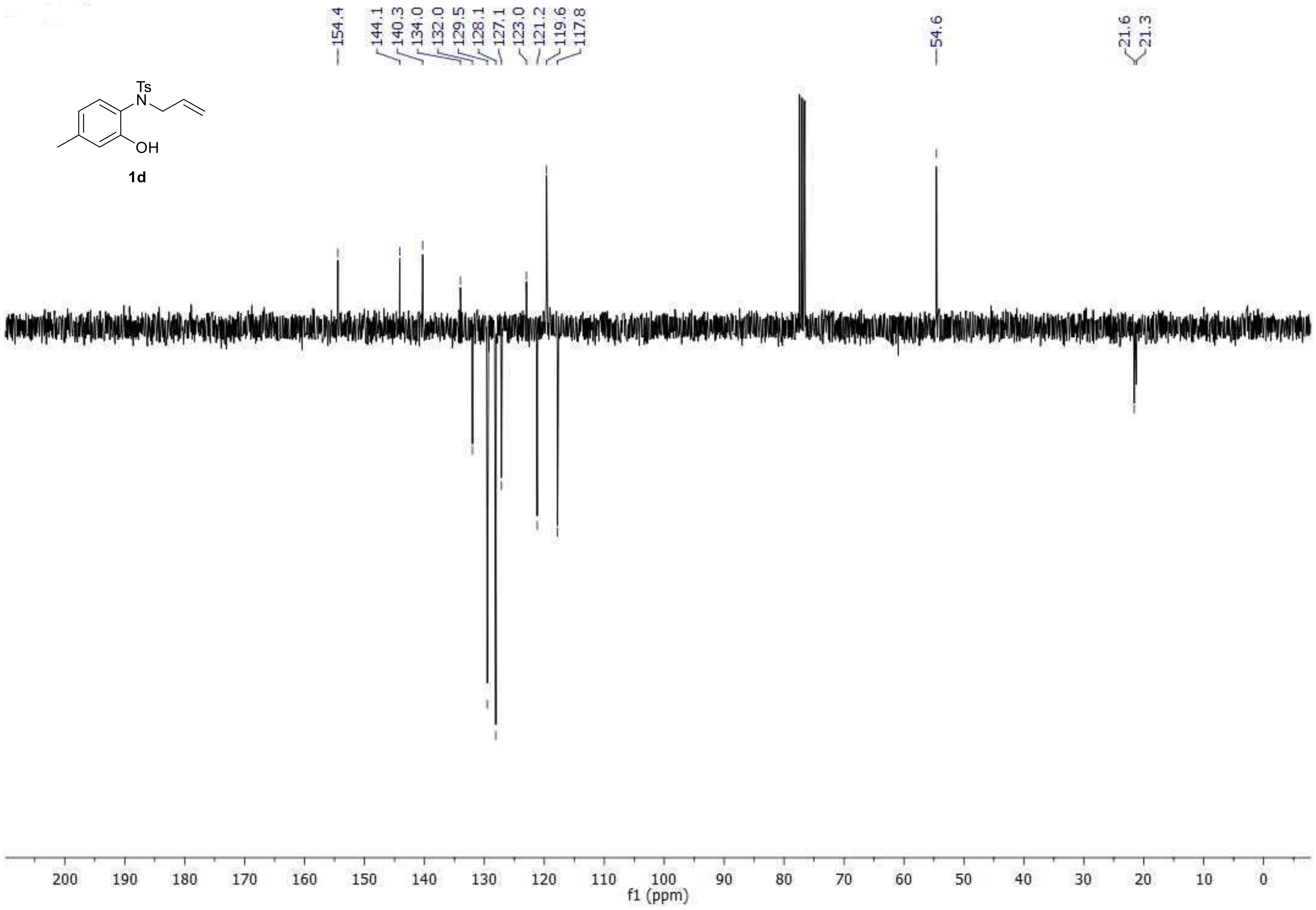

^{1}H NMR (300 MHz, $CDCl_3$) of **1e**

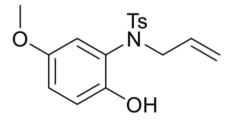

1e

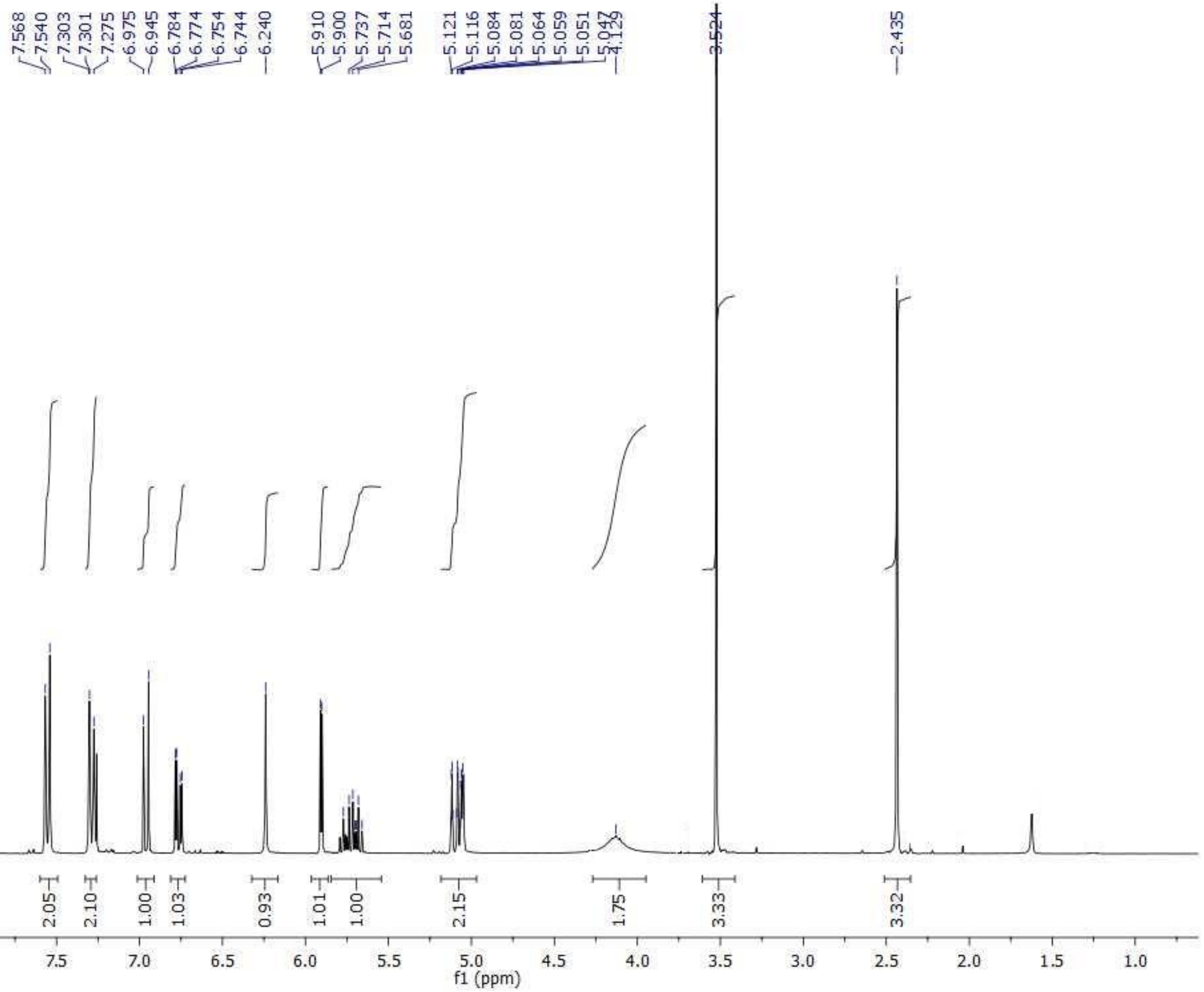

^{13}C NMR (75 MHz, $CDCl_3$) of **1e**

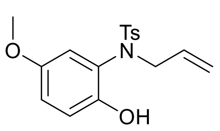

1e

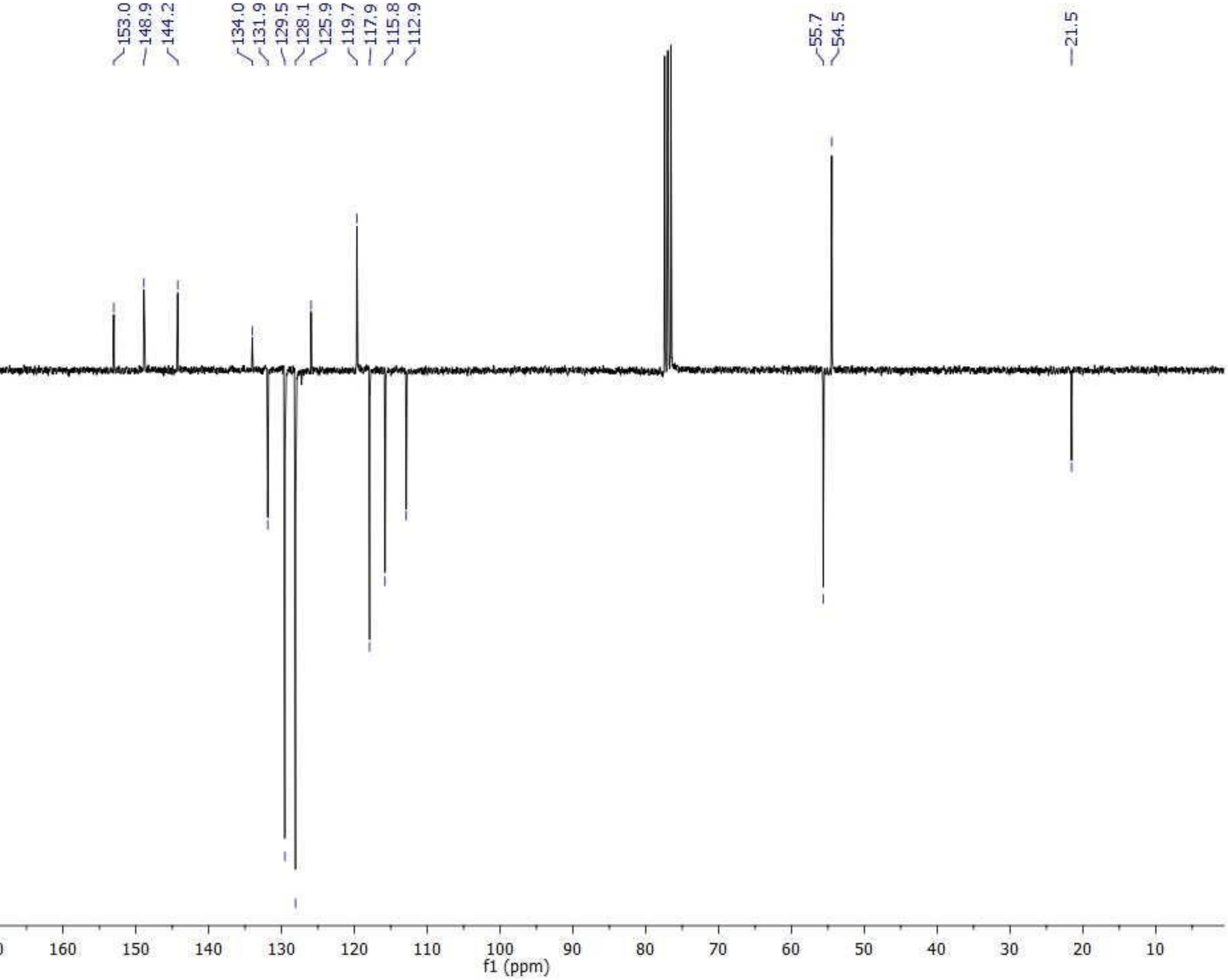

^{1}H NMR (300 MHz, $CDCl_3$) of **1f**

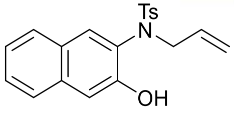

1f

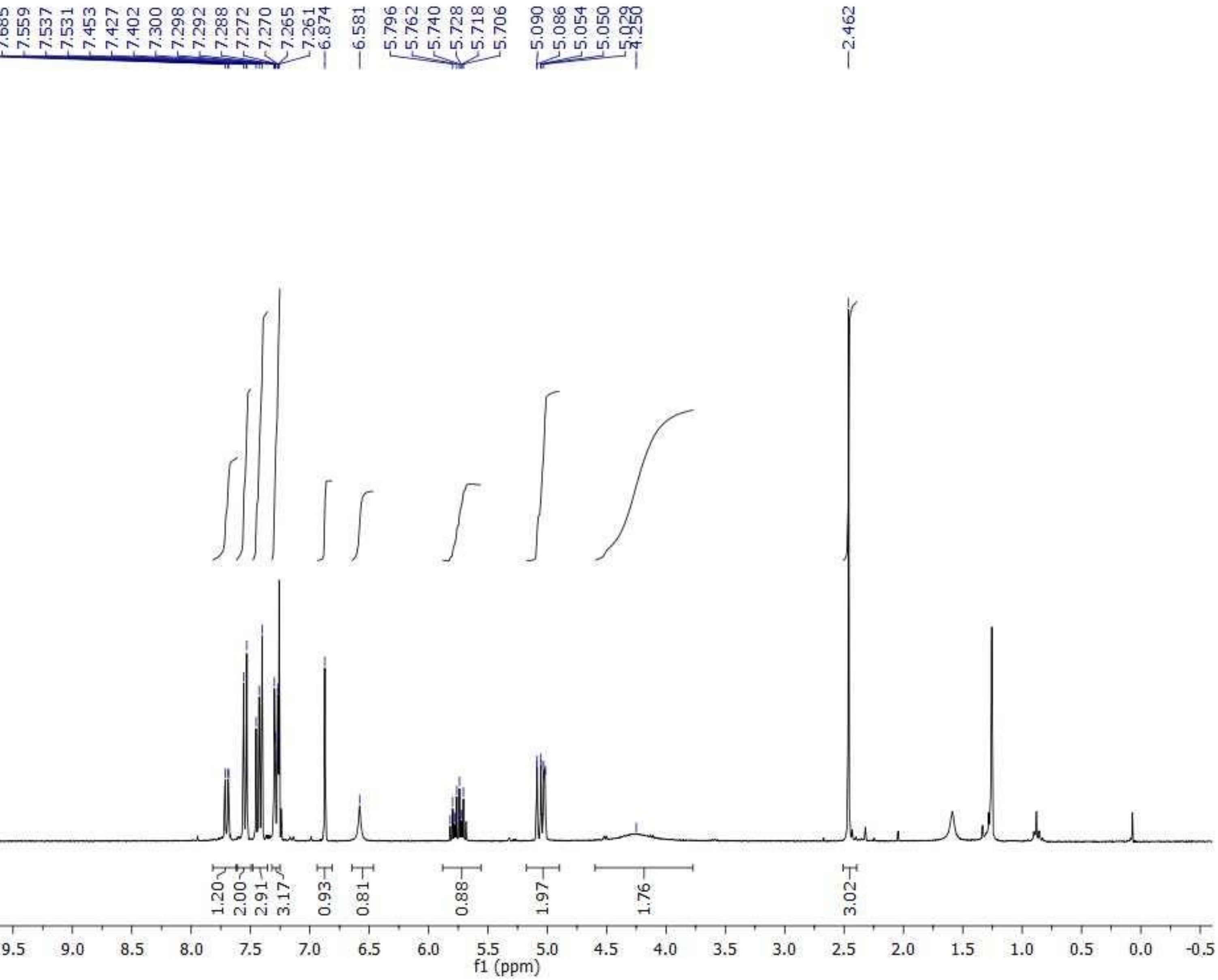

^{13}C NMR (75 MHz, $CDCl_3$) of **1f**

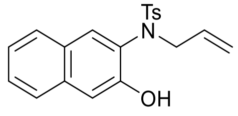

1f

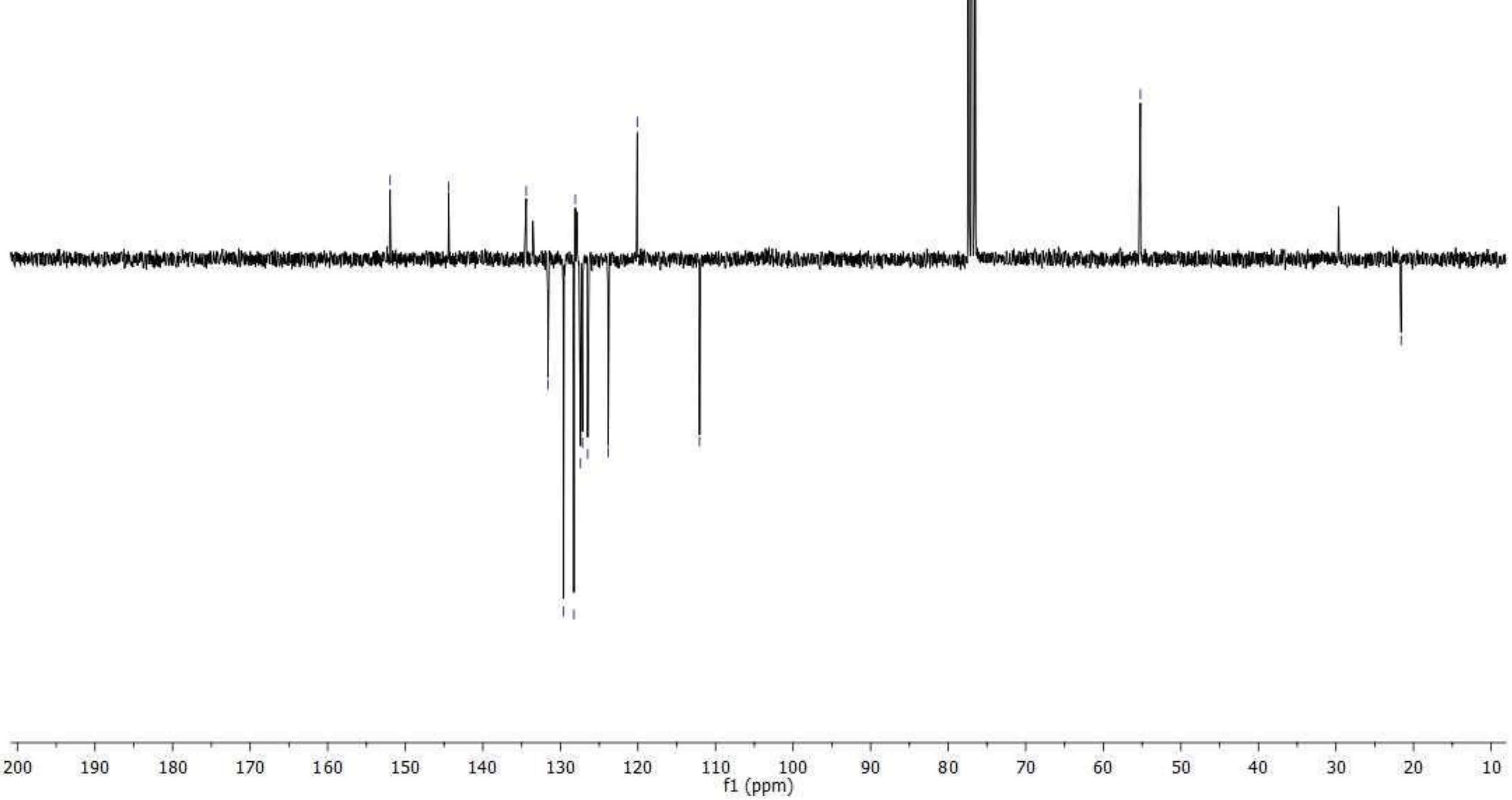

^{1}H NMR (300 MHz, $CDCl_3$) of **1g**

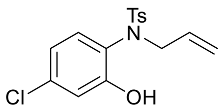

1g

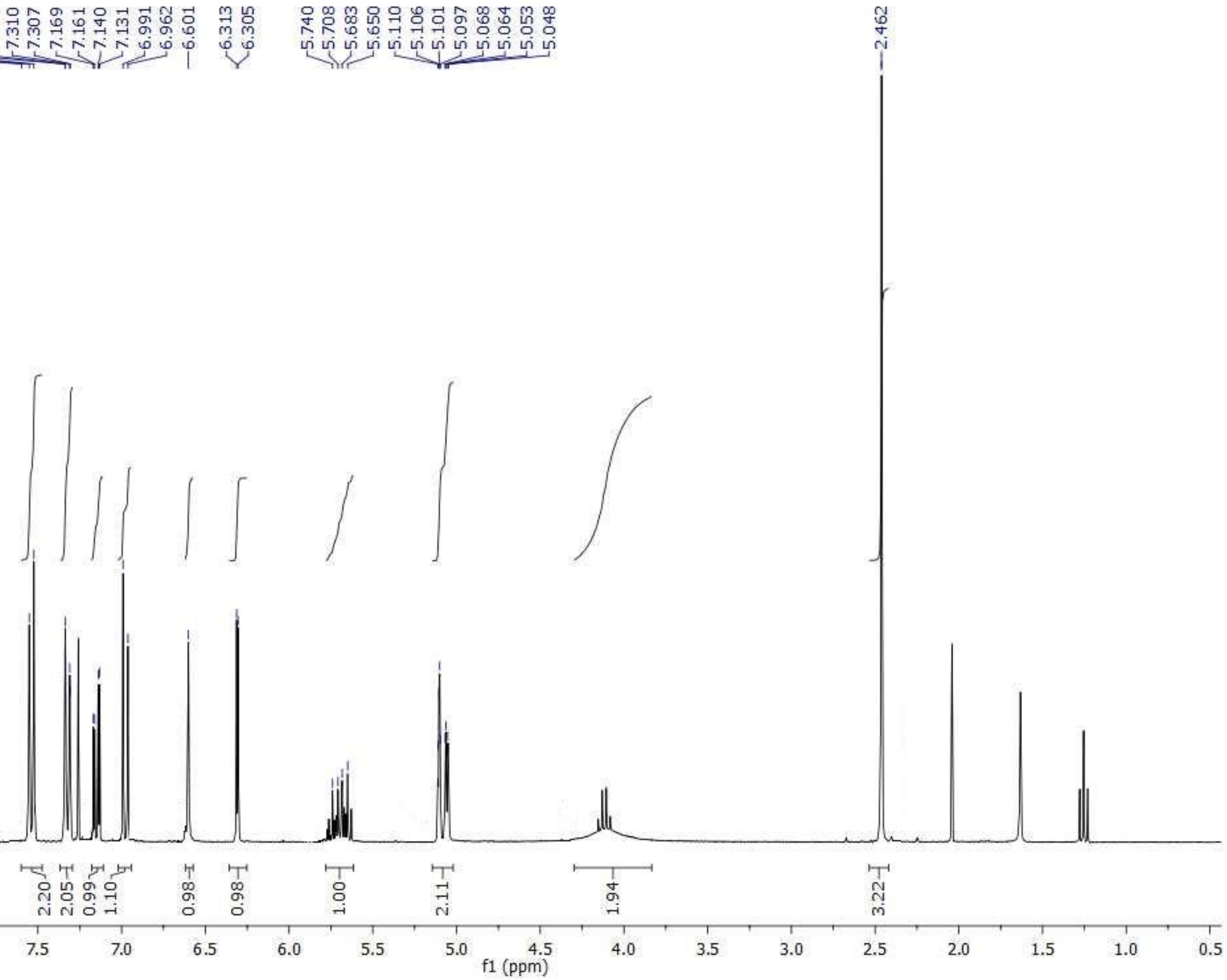

^{13}C NMR (75 MHz, $CDCl_3$) of **1g**

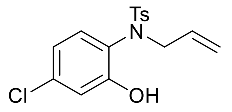

1g

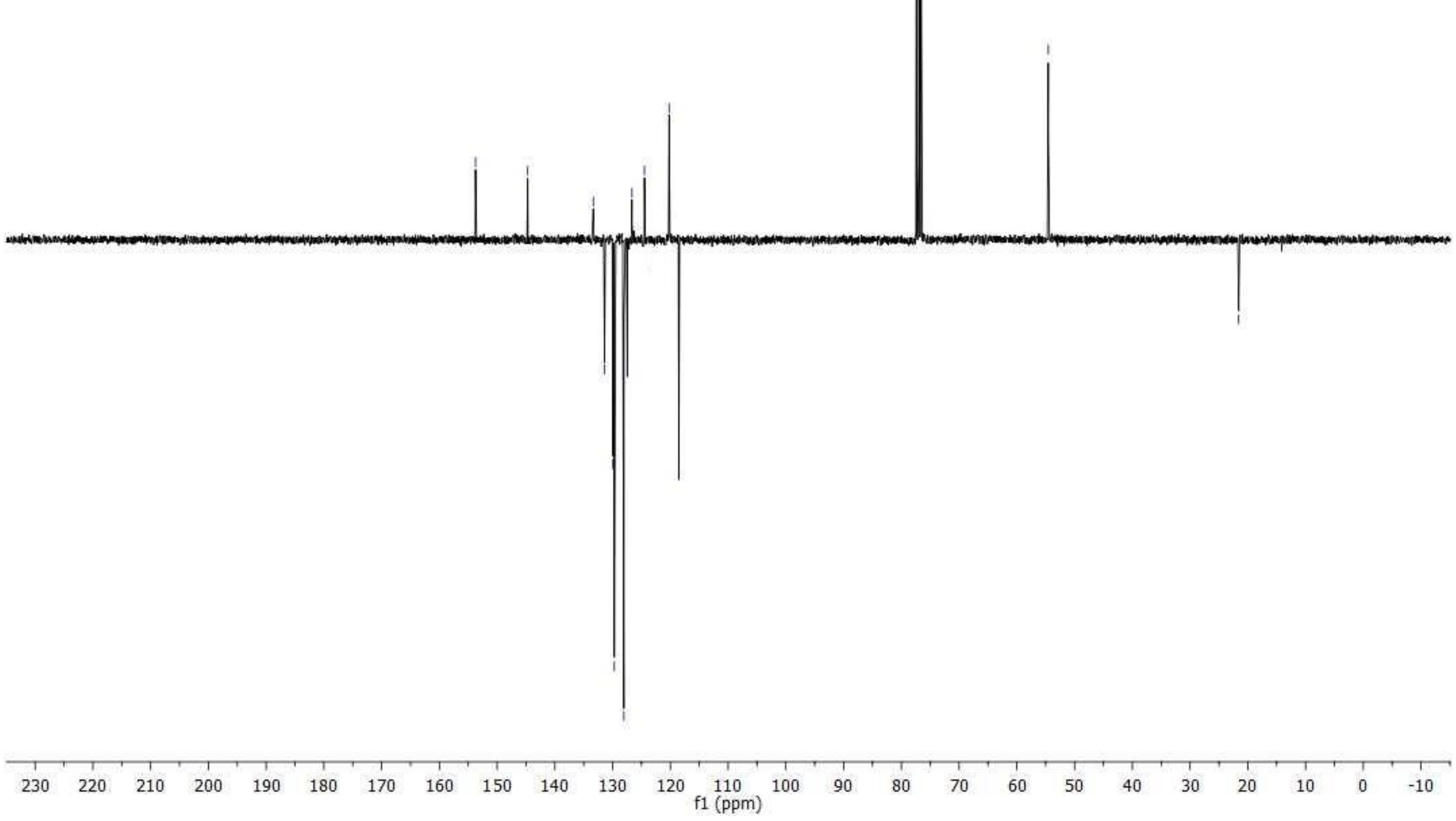

^{1}H NMR (300 MHz, $CDCl_3$) of **1h**

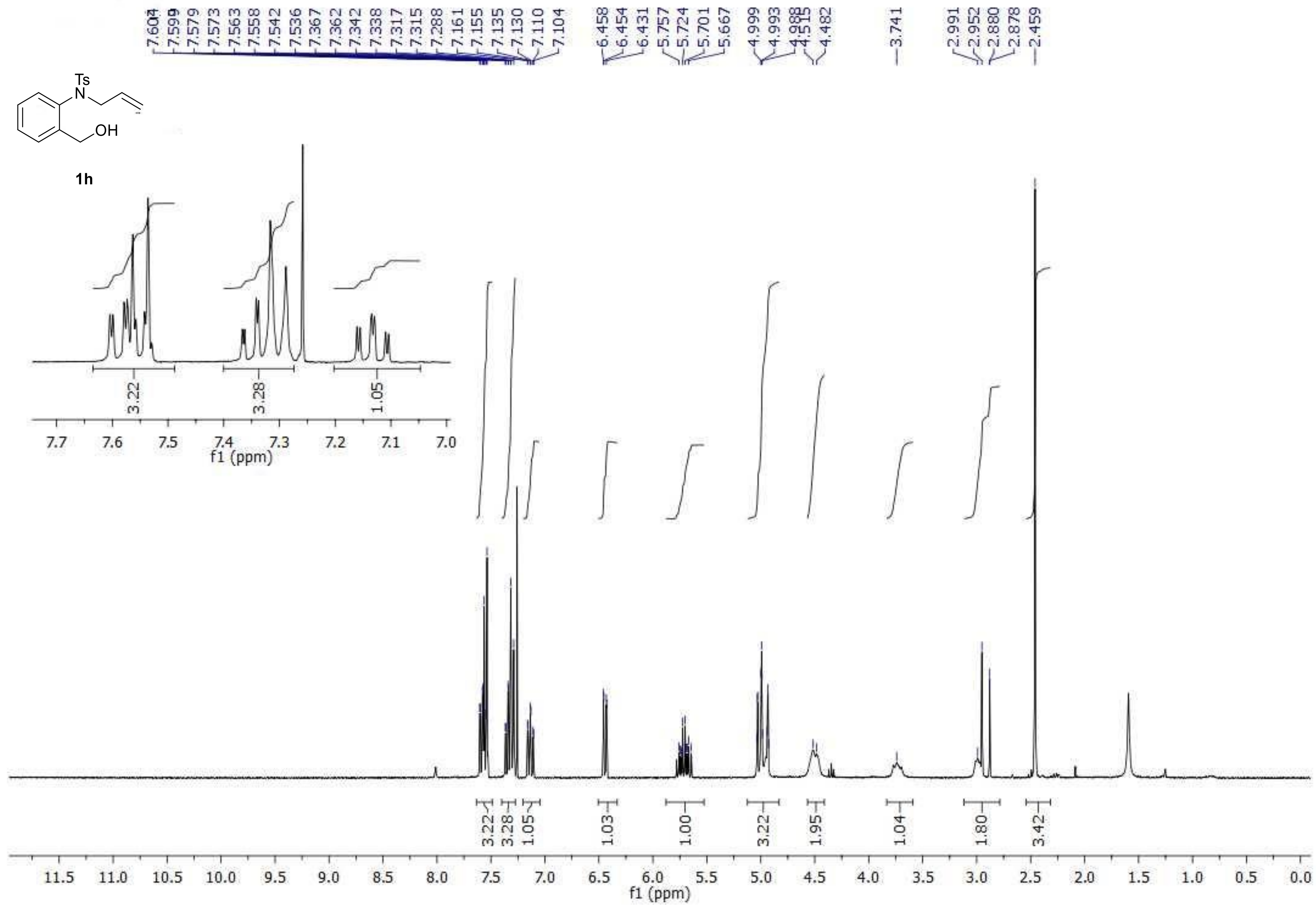

^{13}C NMR (75 MHz, $CDCl_3$) of **1h**

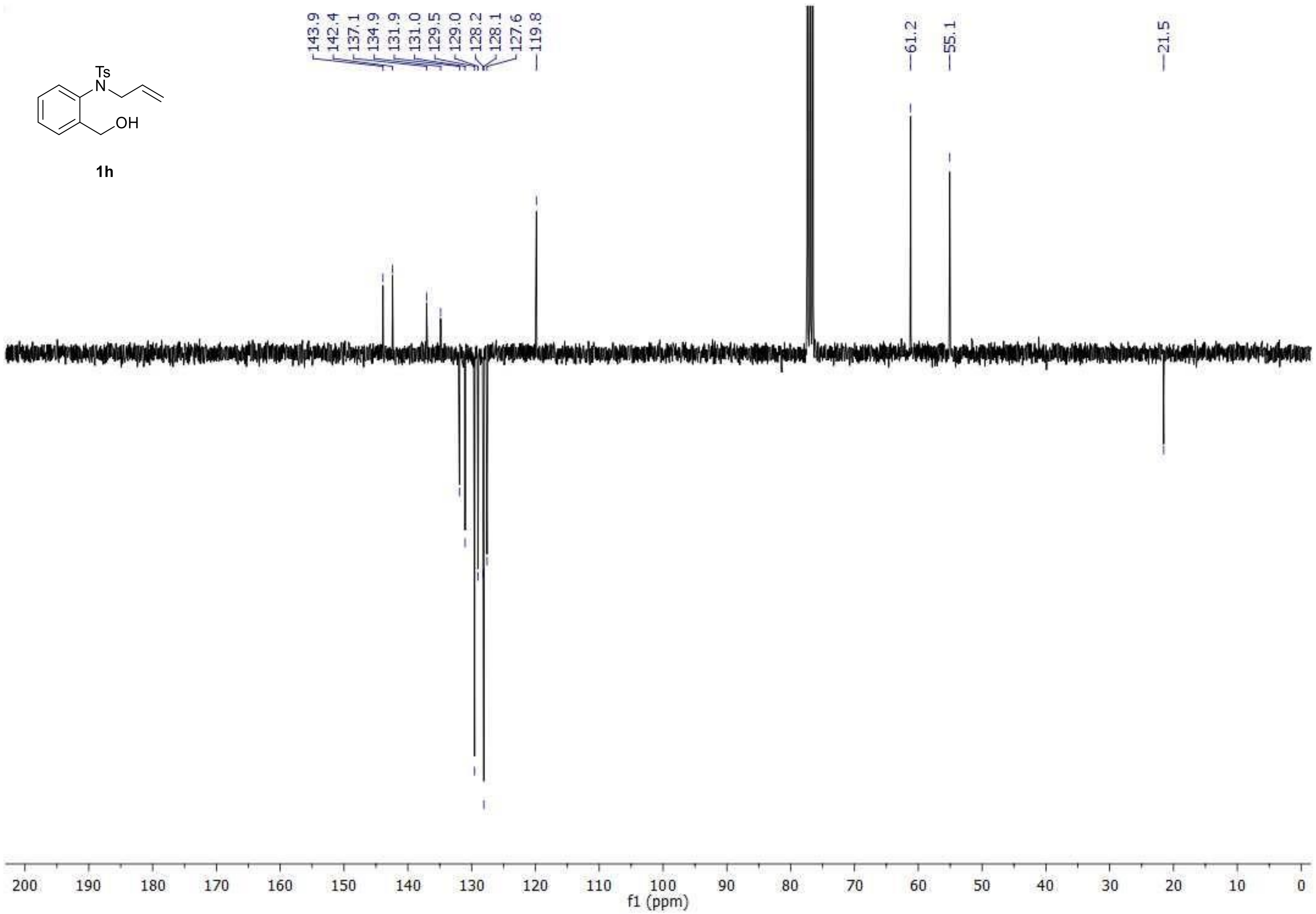

^{1}H NMR (300 MHz, $CDCl_3$) of **3aa**

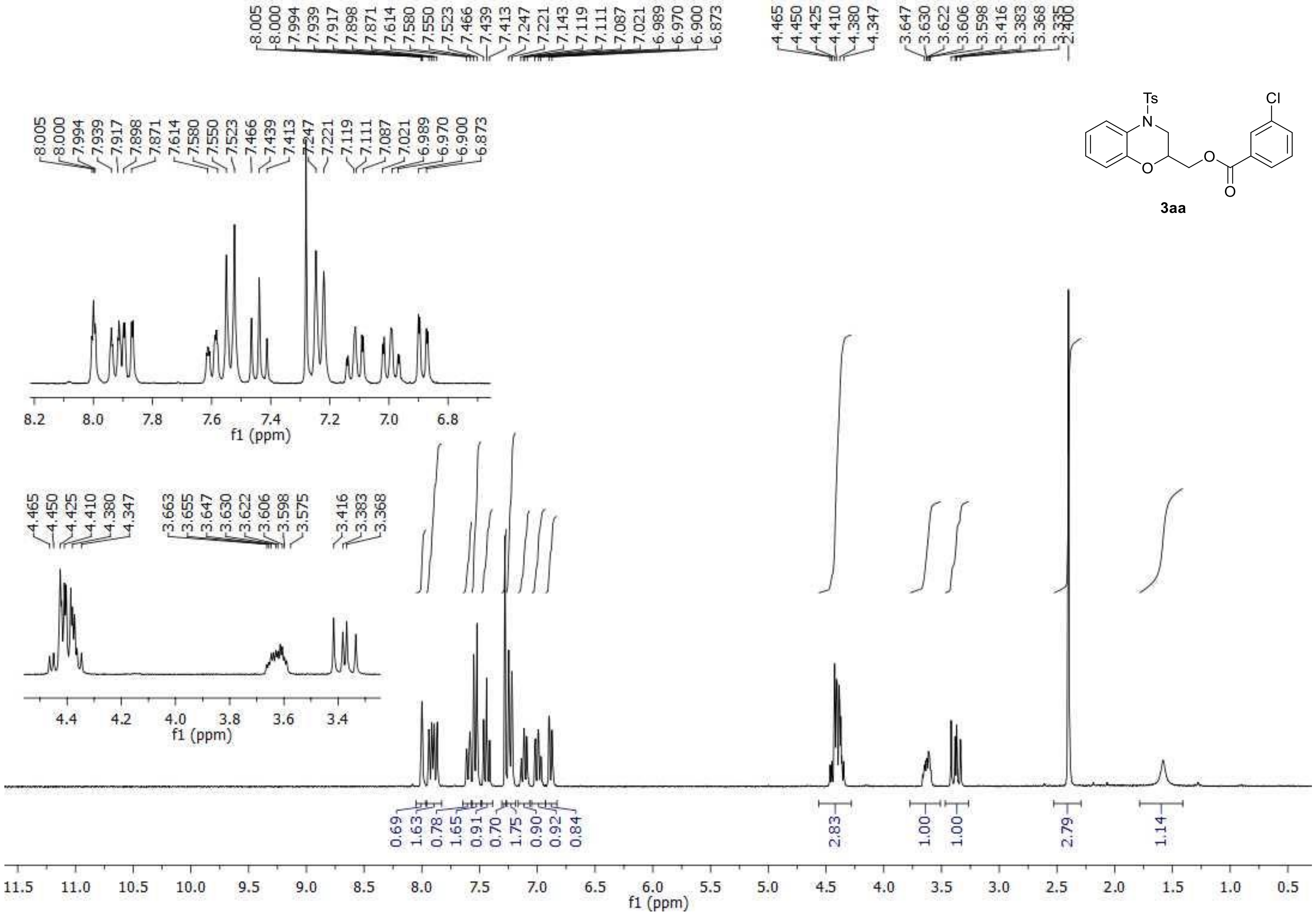

^{13}C NMR (75 MHz, $CDCl_3$) of **3aa**

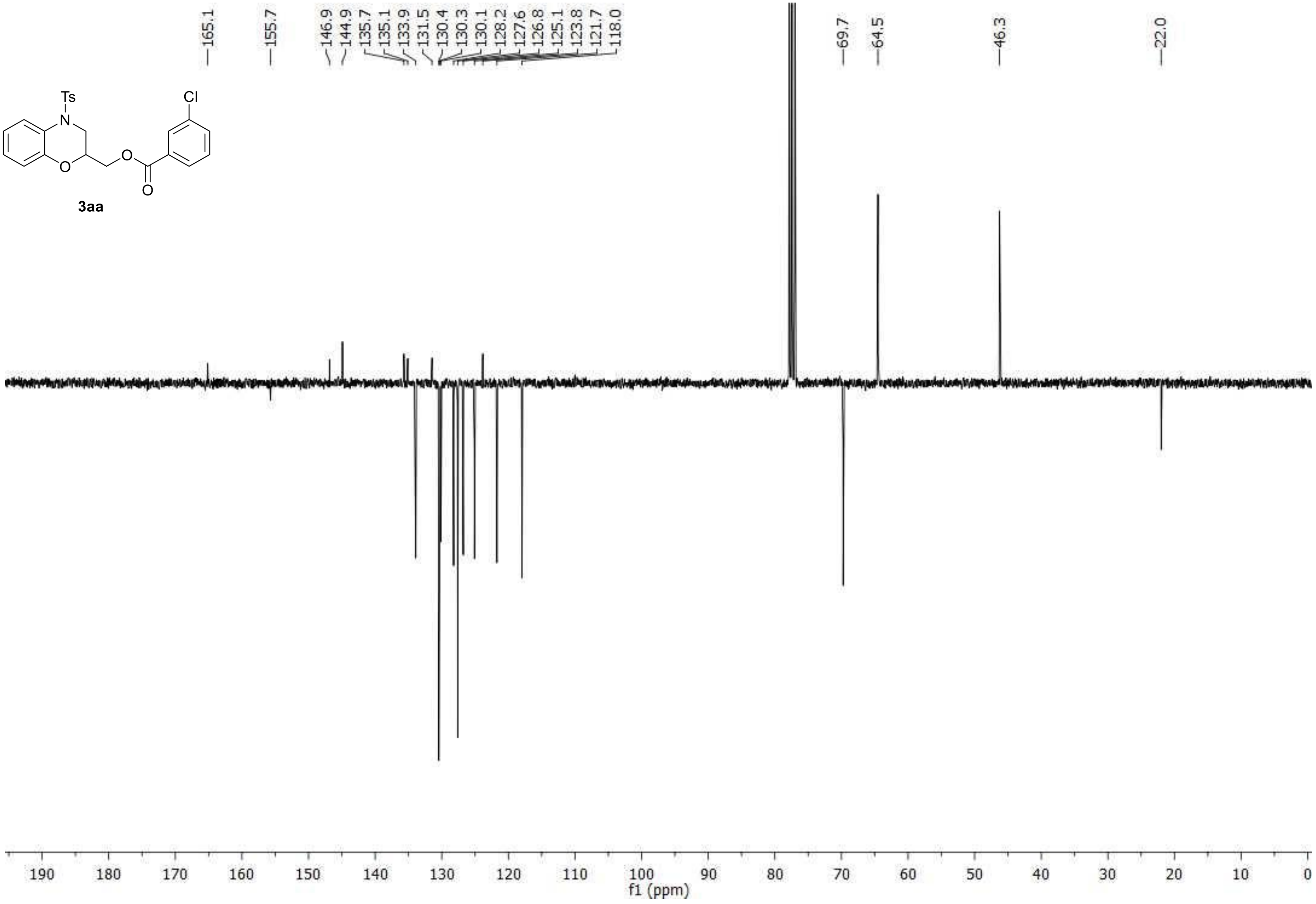

HSQC NMR (300 MHz, 75 MHz, $CDCl_3$) of **3aa**

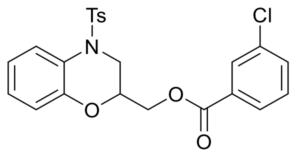

3aa

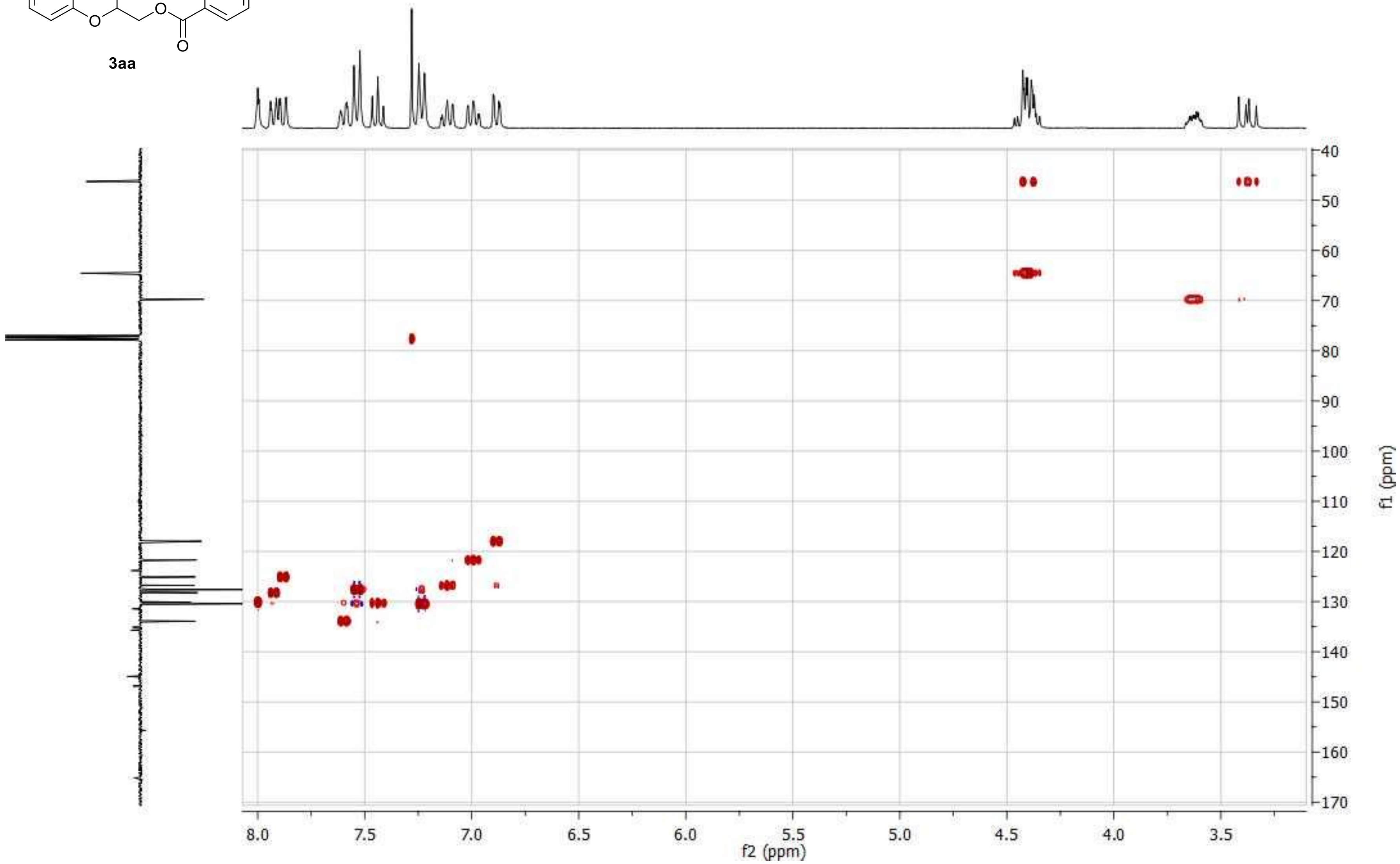

^{1}H NMR (300 MHz, $CDCl_3$) of **3ad**

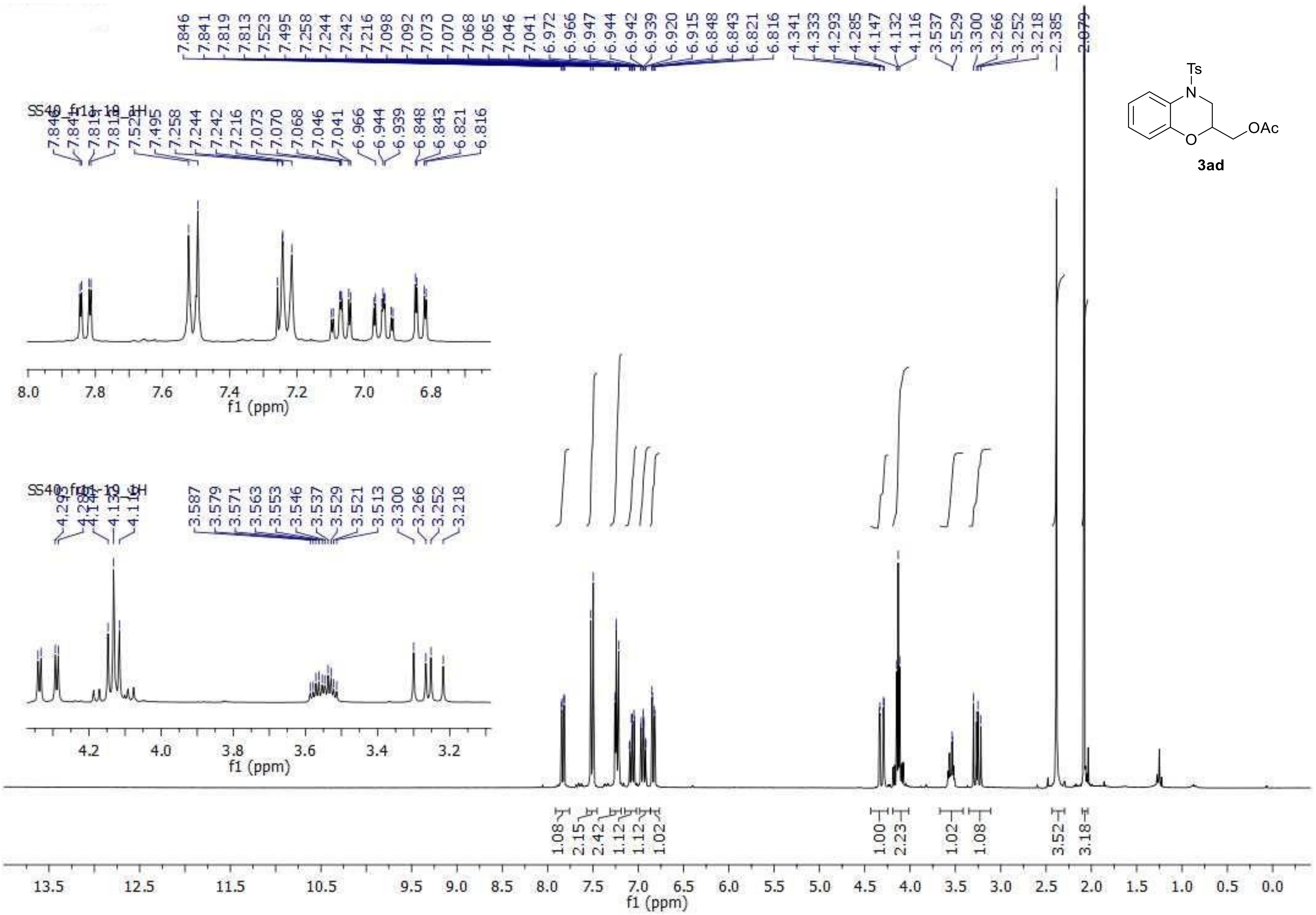

^{13}C NMR (75 MHz, $CDCl_3$) of **3ad**

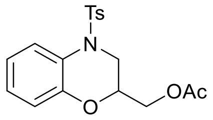

3ad

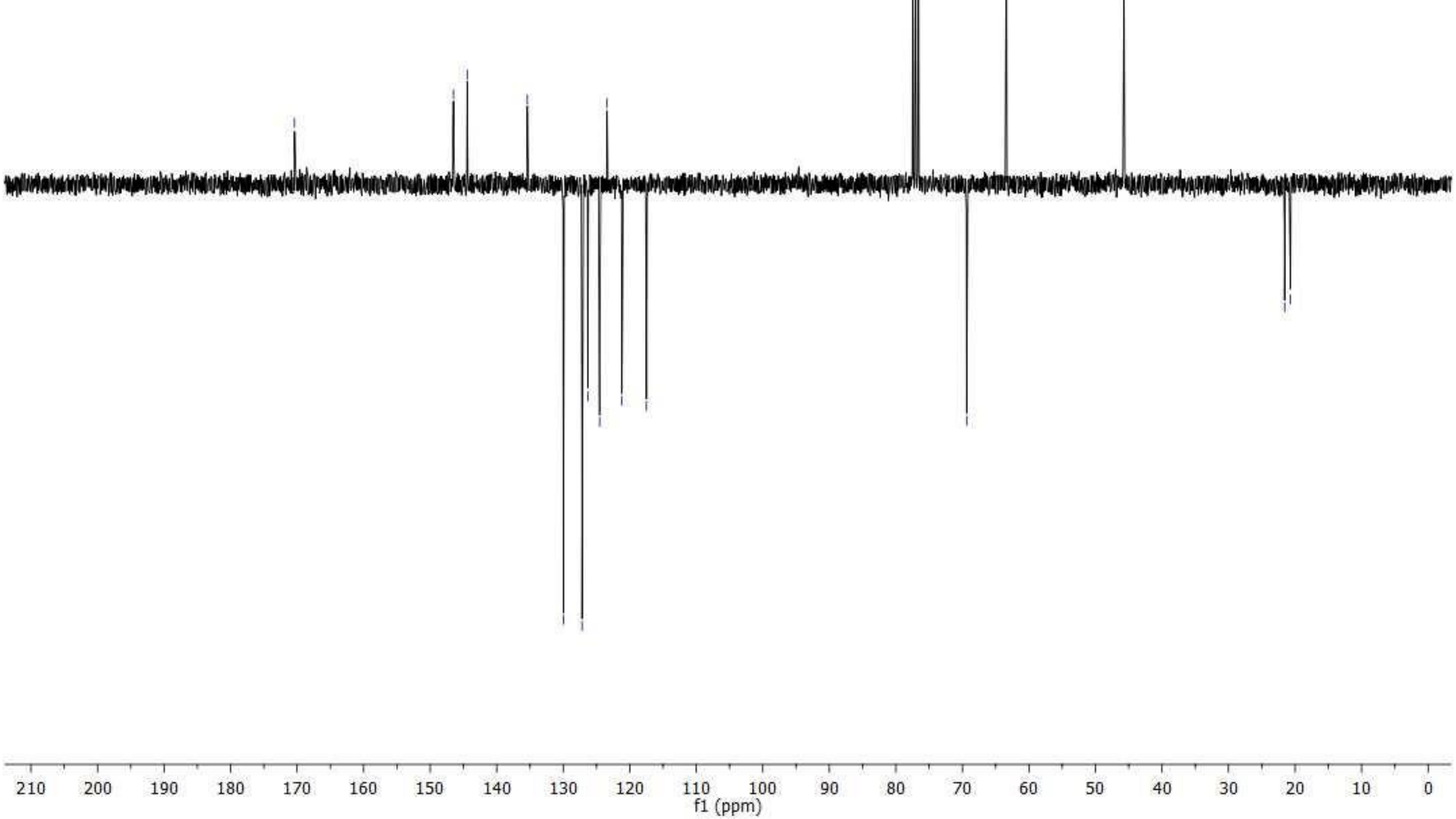

^1H NMR (300 MHz, $CDCl_3$) of **3ab**

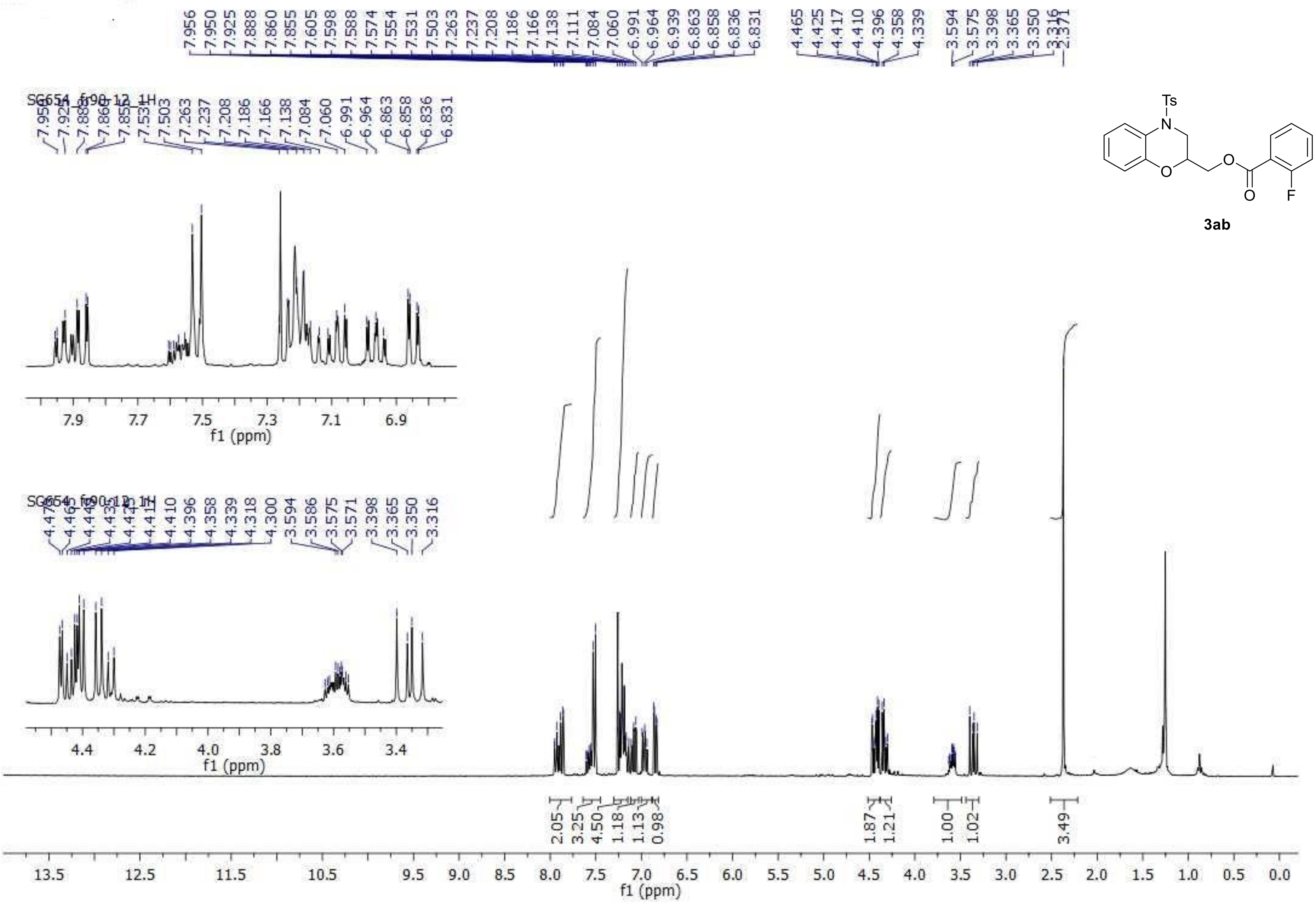

3ab

^{13}C NMR (75 MHz, $CDCl_3$) of **3ab**

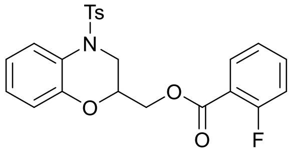

3ab

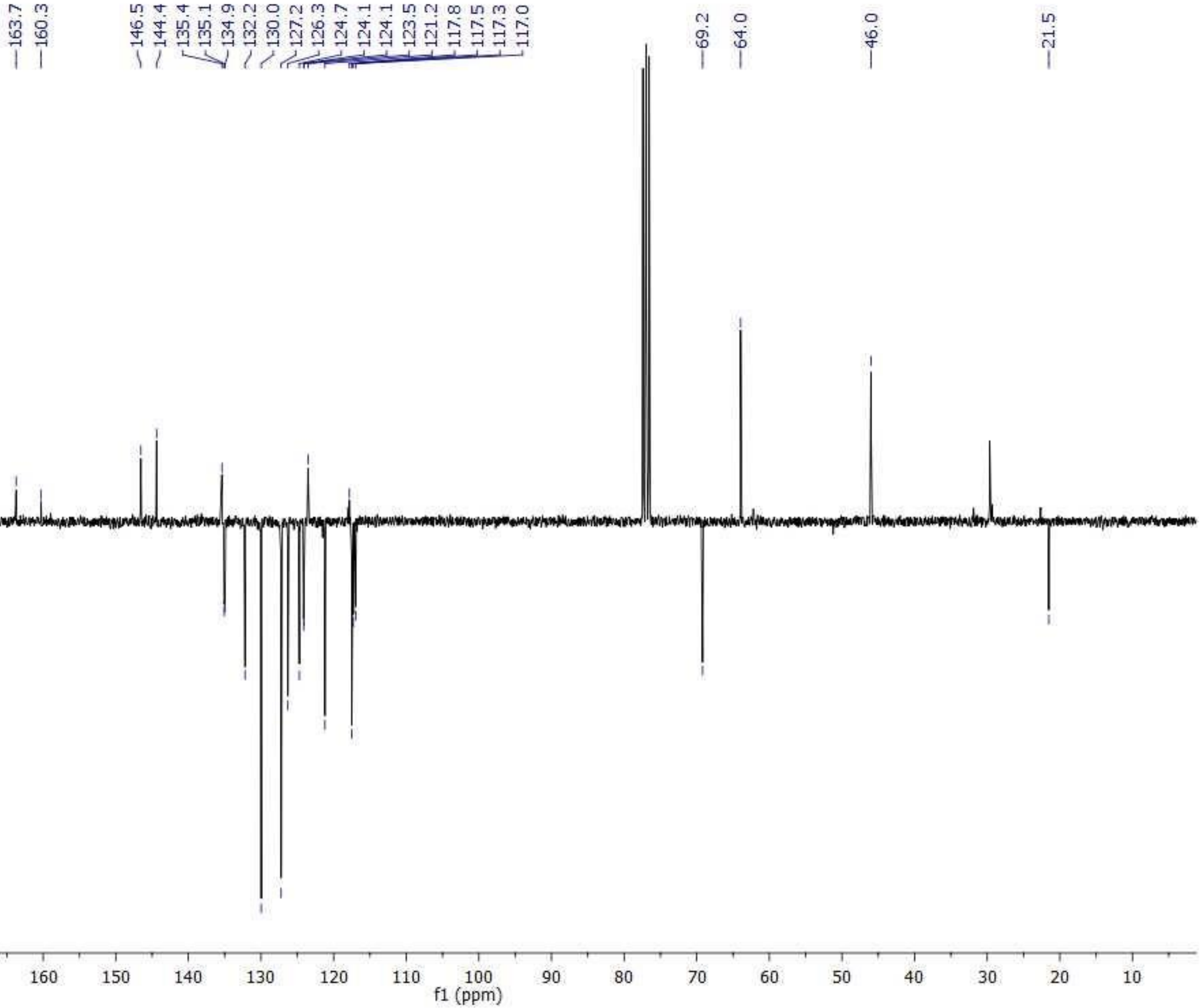

^{1}H NMR (300 MHz, $CDCl_3$) of **3ac**

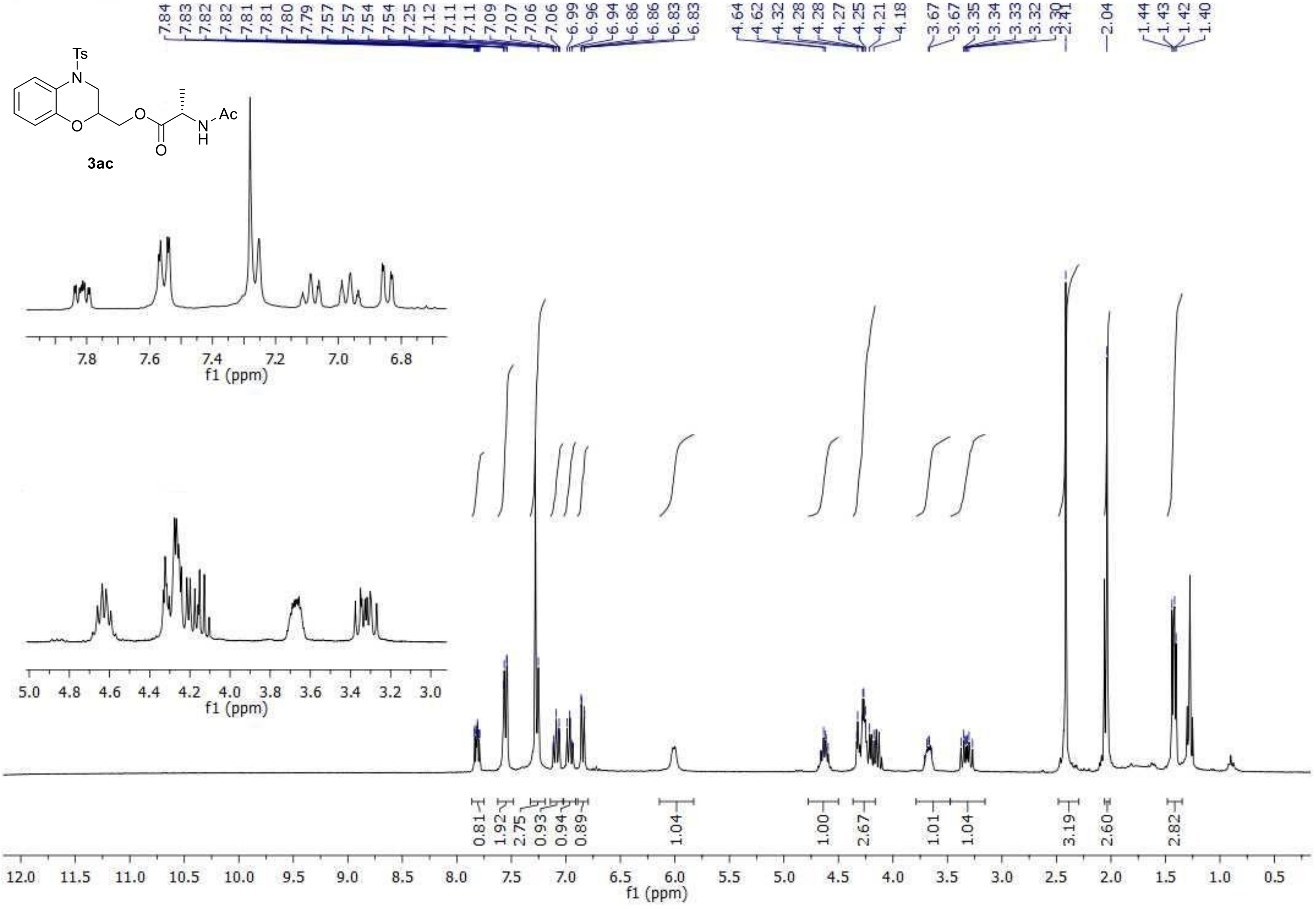

^{13}C NMR (75 MHz, $CDCl_3$) of **3ac**

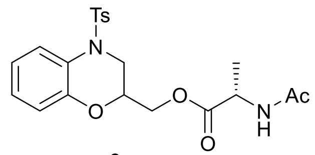

3ac

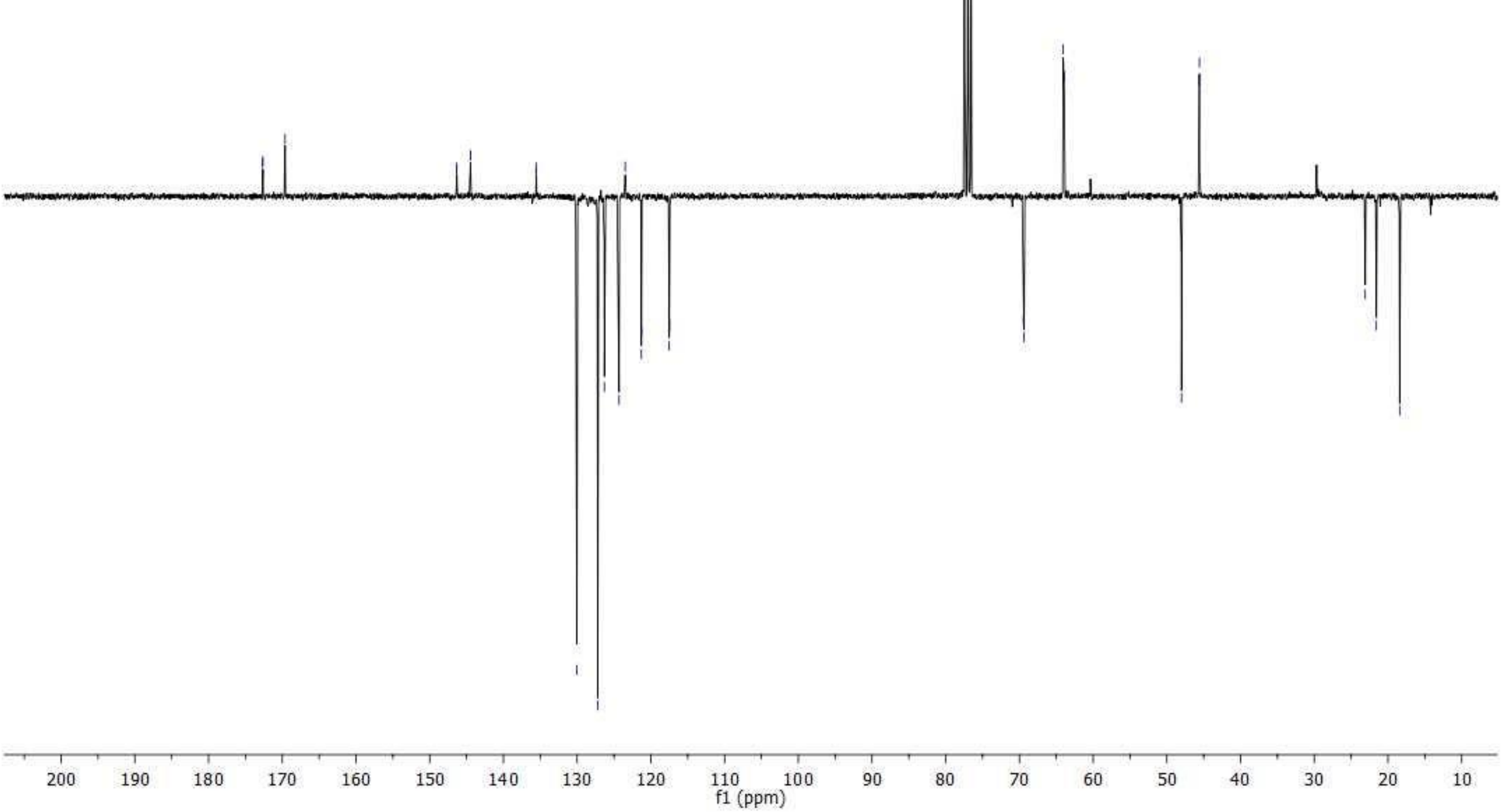

HSQC NMR (300 MHz, 75 MHz, $CDCl_3$) of **3ac**

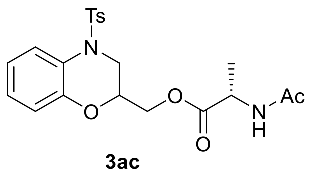

3ac

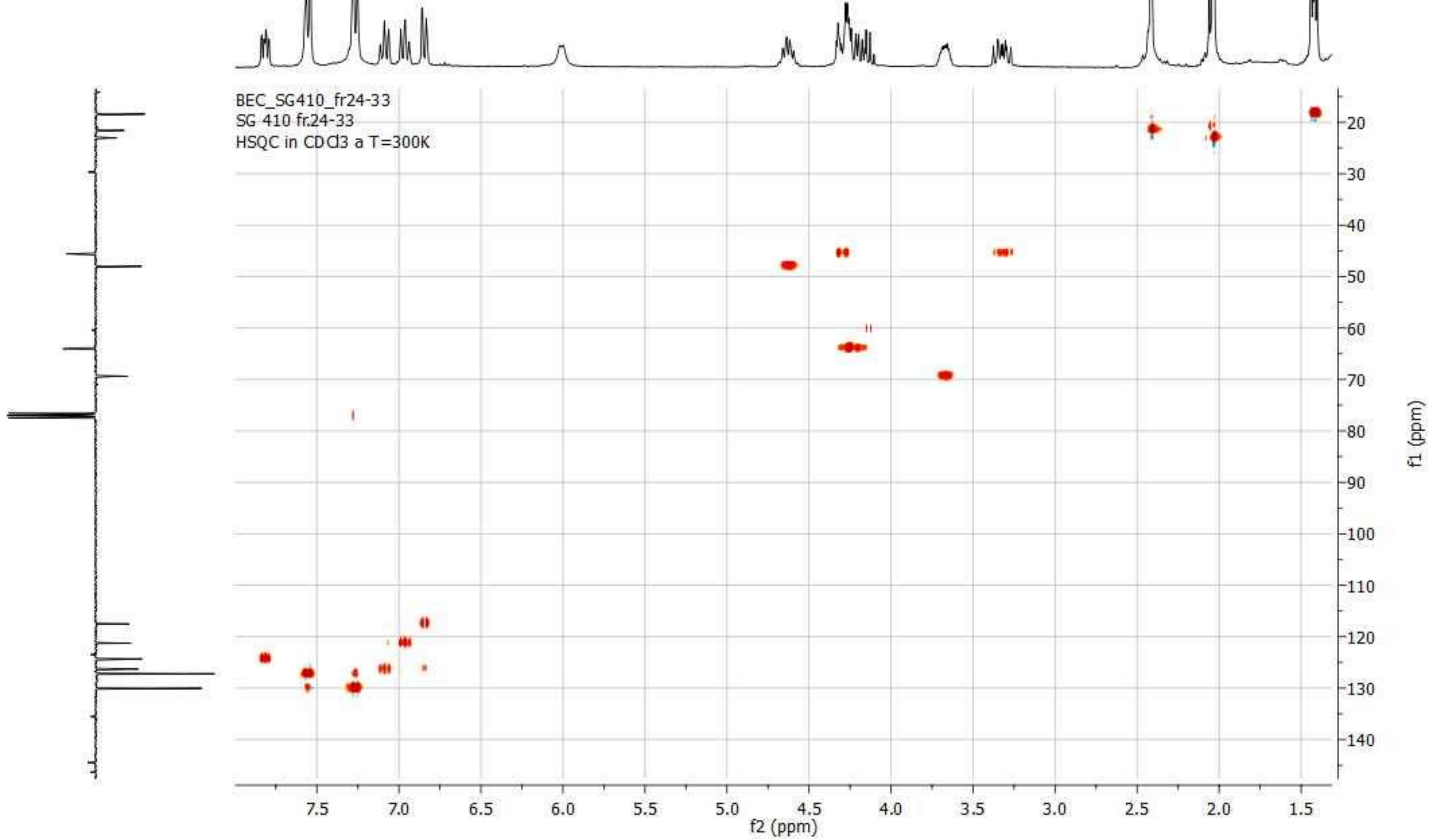

^{1}H NMR (300 MHz, $CDCl_3$) of **3ba**

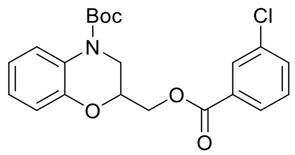

3ba

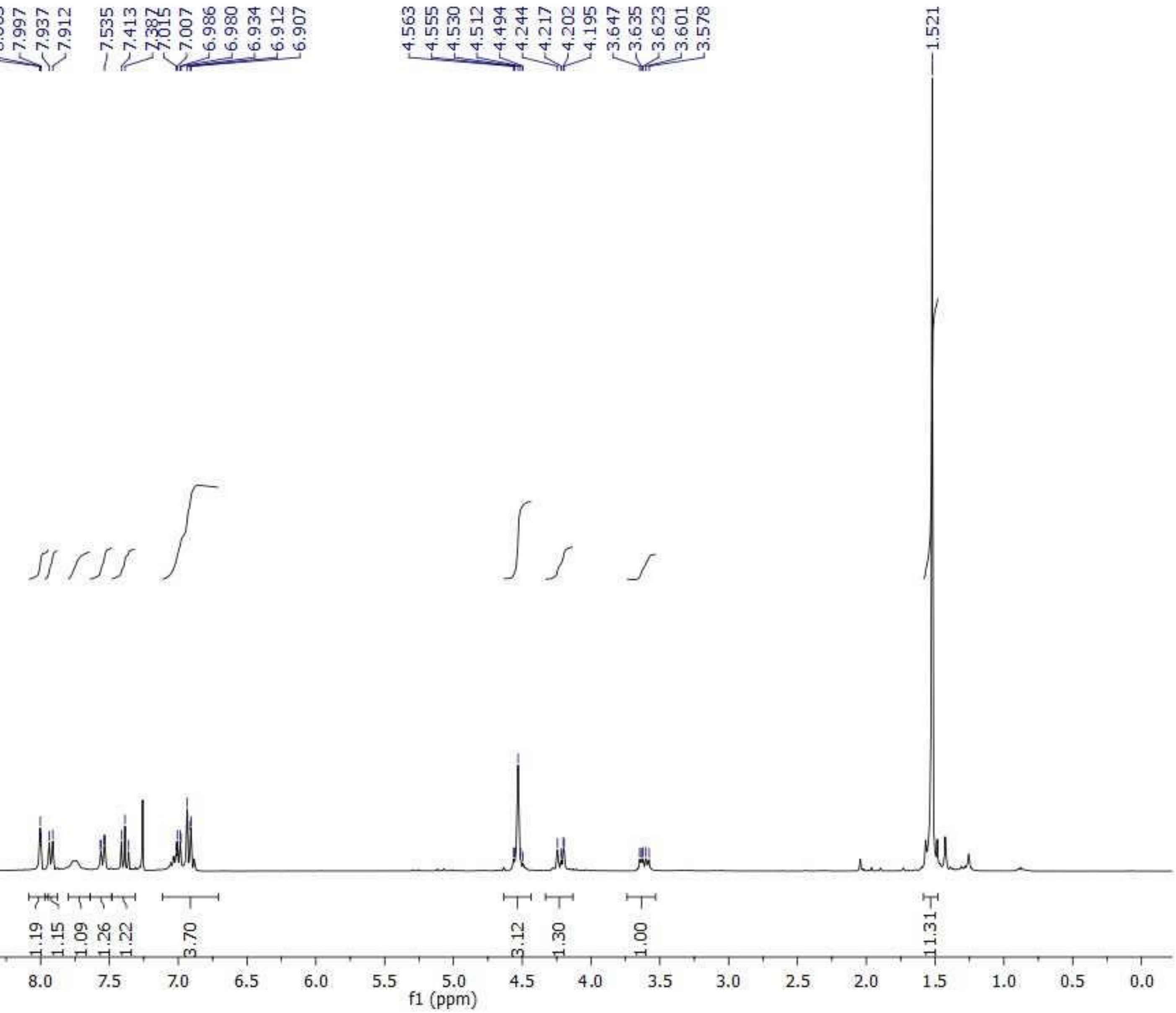

^{1}H NMR (300 MHz, $CDCl_3$) of **3cb**

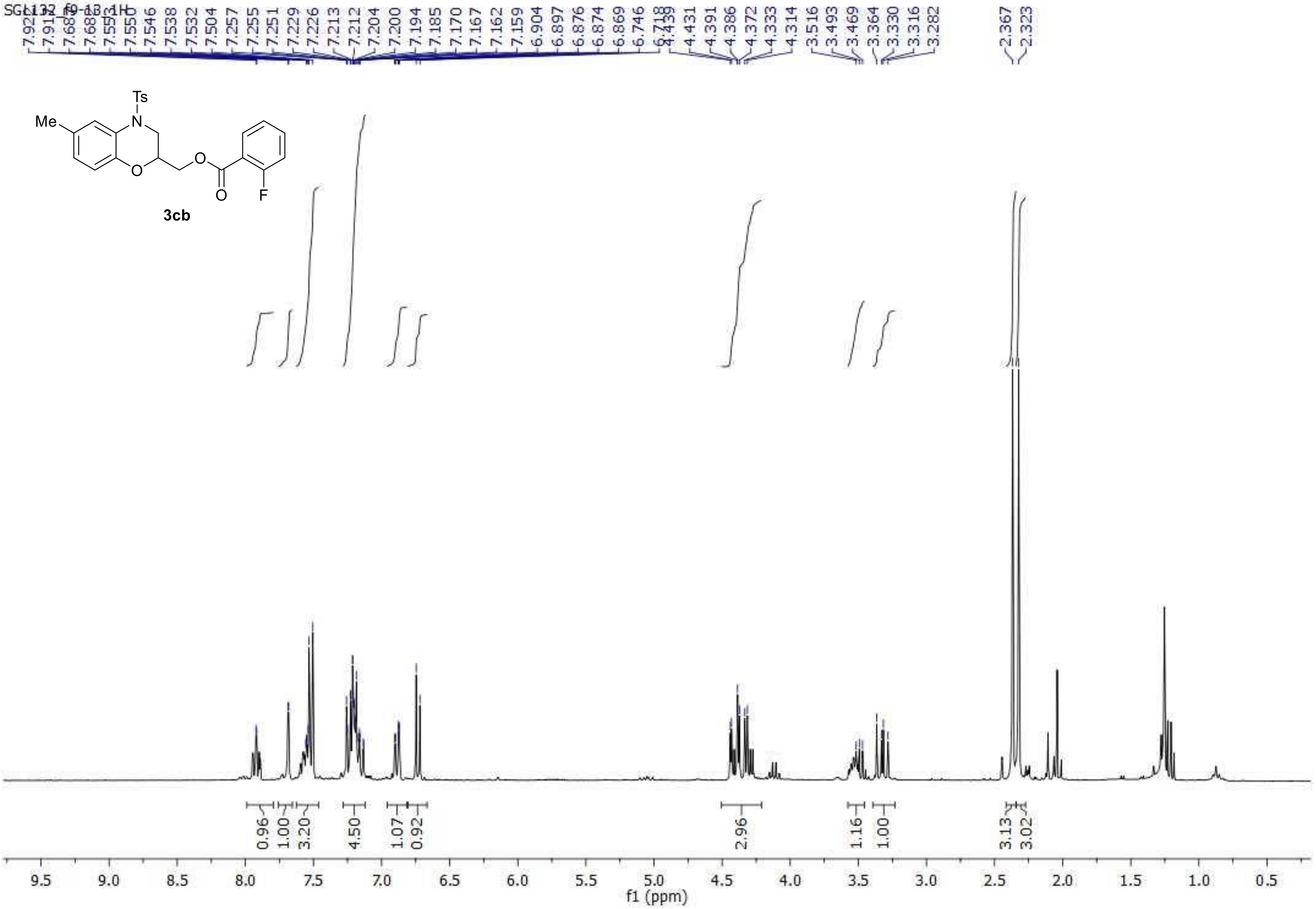

3cb

^{13}C NMR (75 MHz, $CDCl_3$) of **3cb**

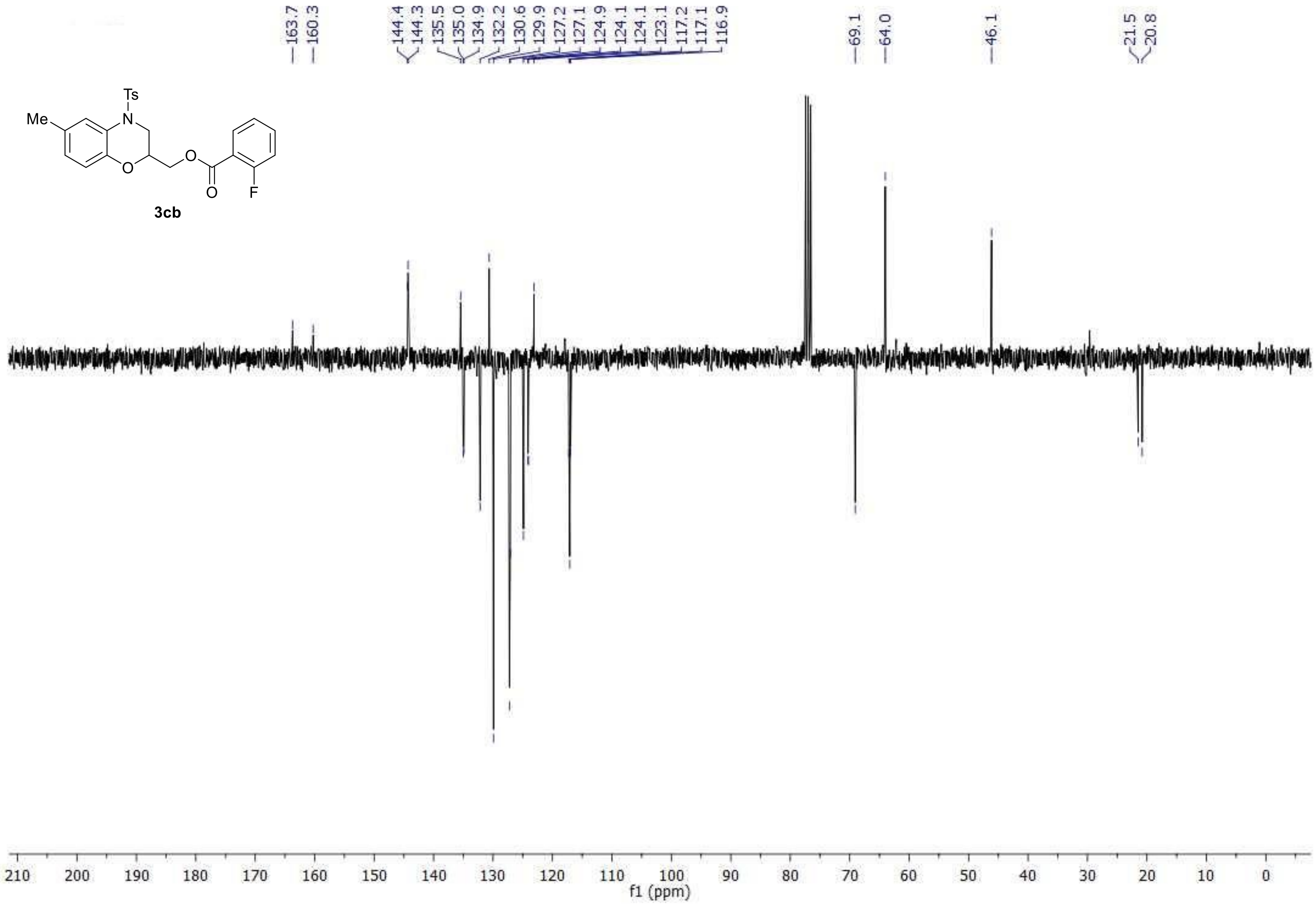

^{1}H NMR (300 MHz, $CDCl_3$) of **3db**

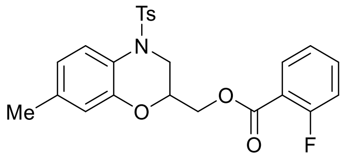

3db

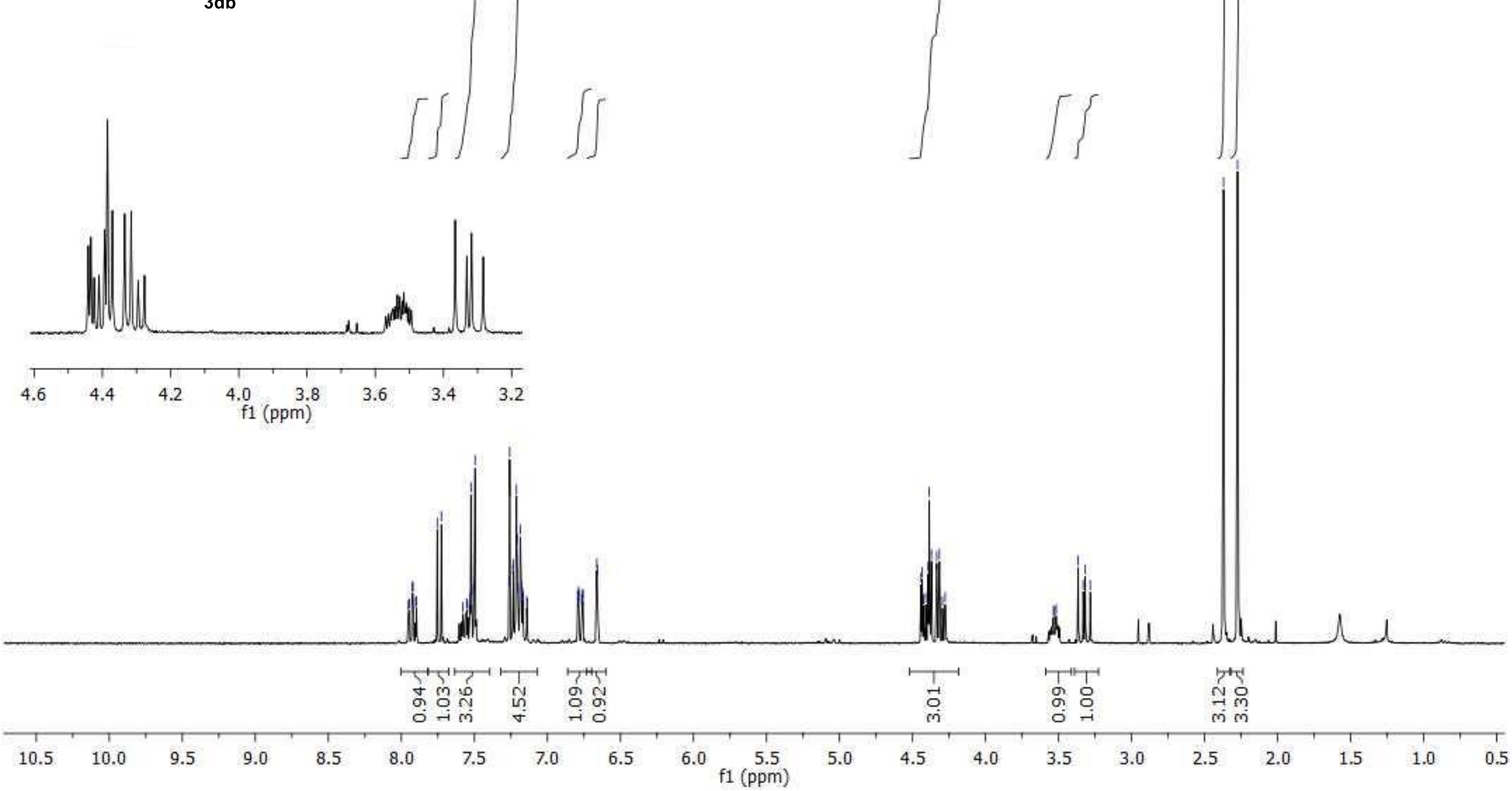

^{13}C NMR (75 MHz, $CDCl_3$) of **3db**

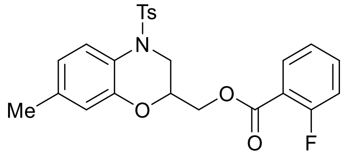

3db

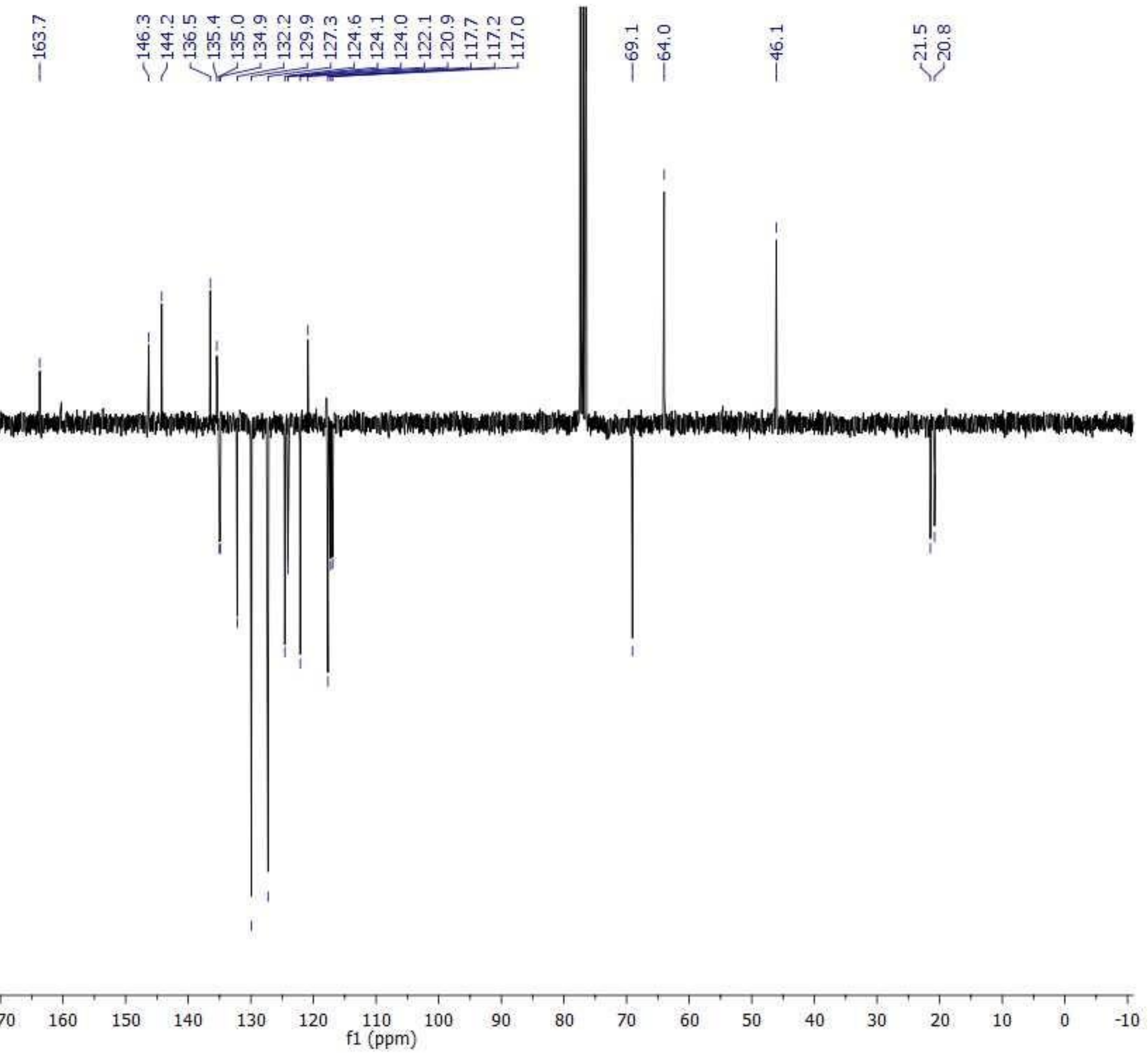

^{1}H NMR (300 MHz, $CDCl_3$) of **3eb**

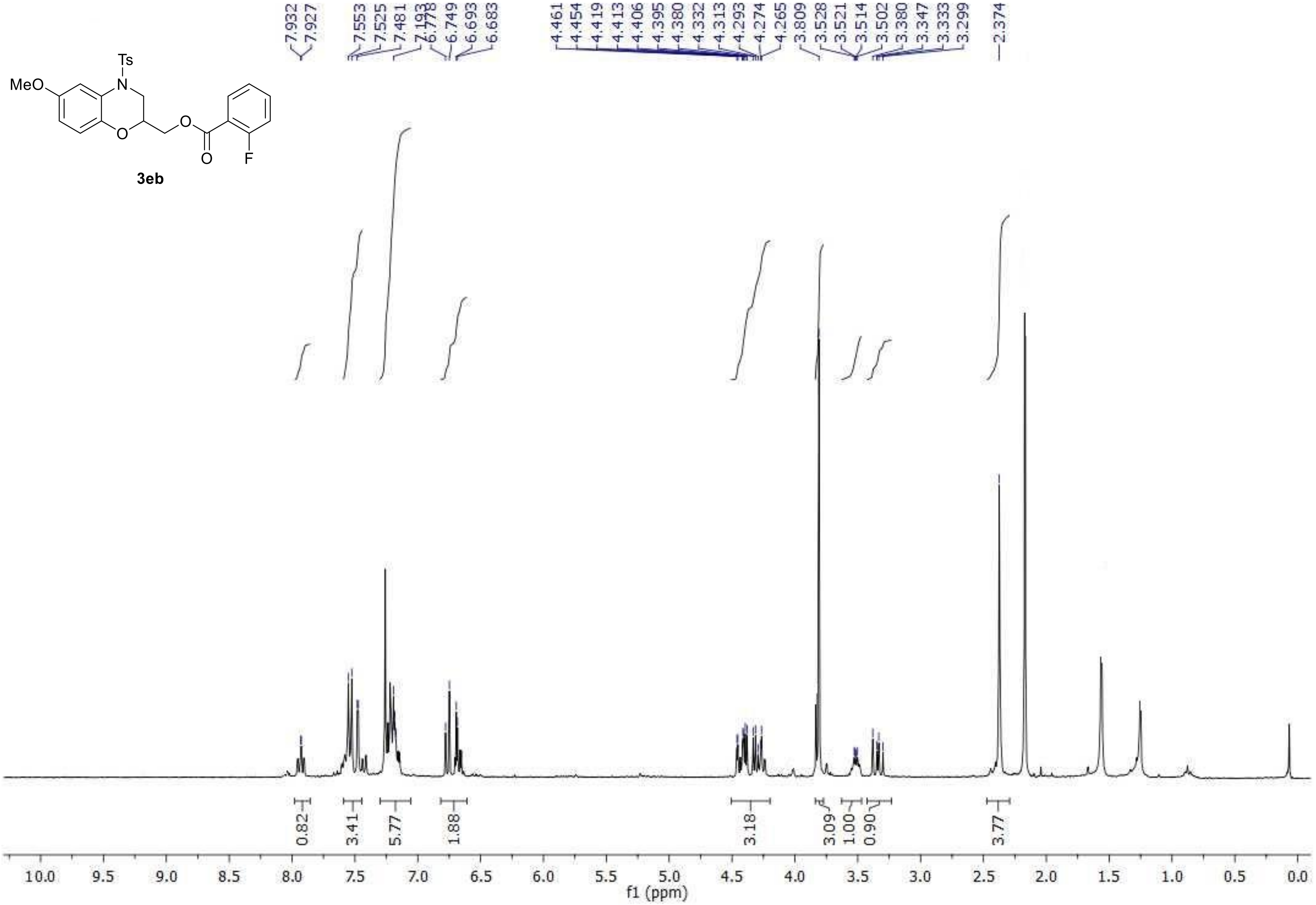

^{13}C NMR (75 MHz, $CDCl_3$) of **3eb**

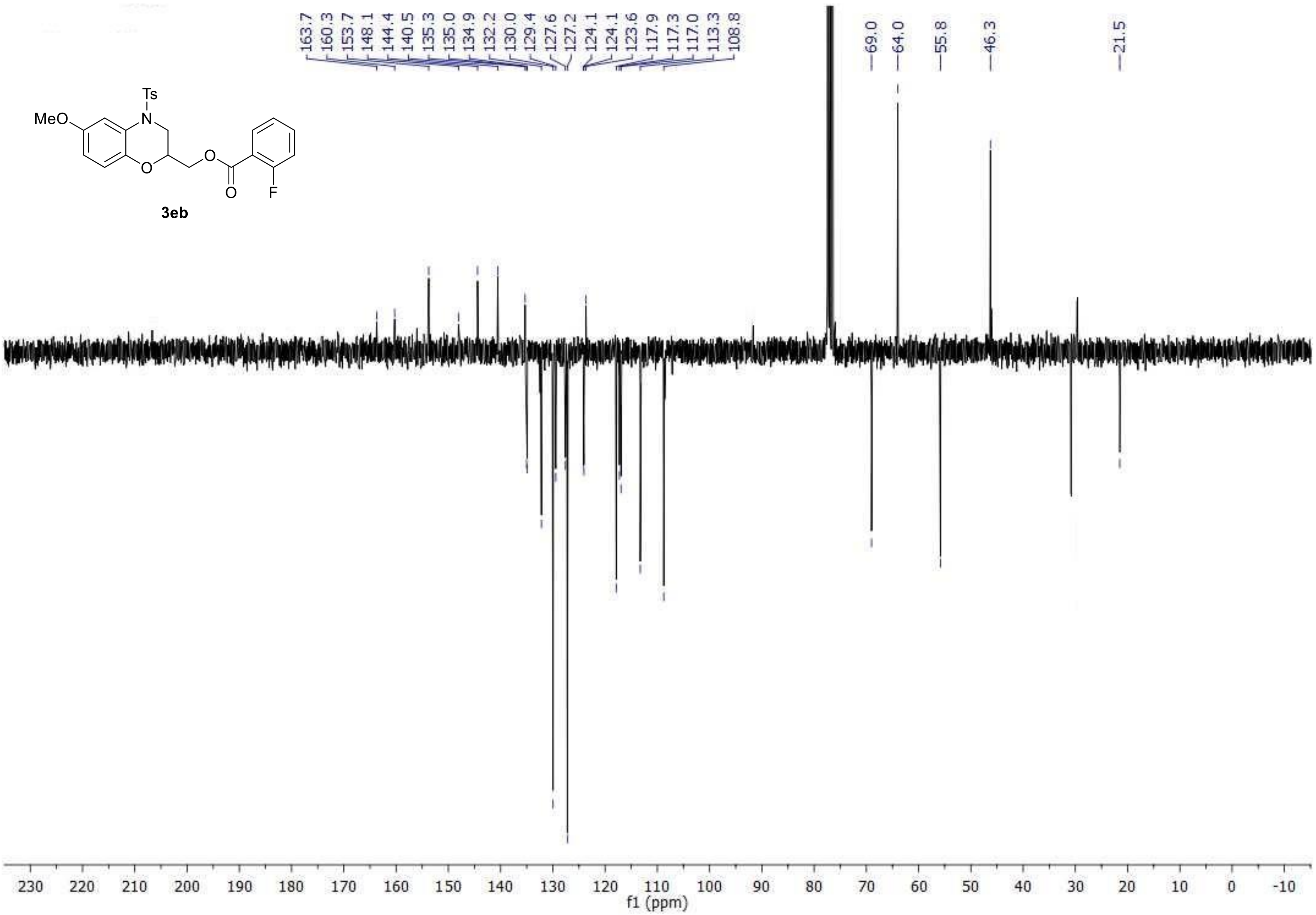

^{1}H NMR (300 MHz, $CDCl_3$) of **3fb**

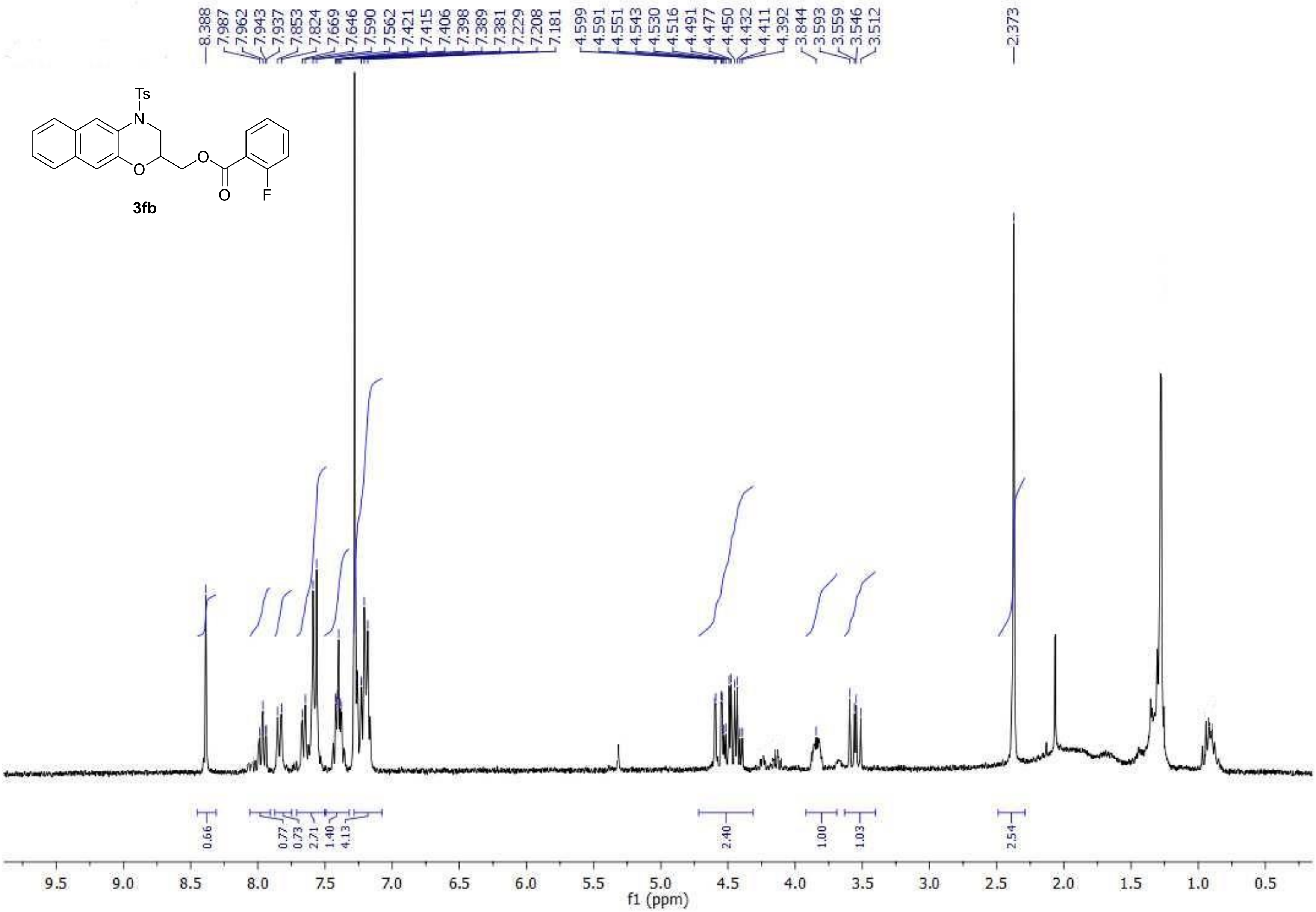

^{13}C NMR (75 MHz, $CDCl_3$) of **3fb**

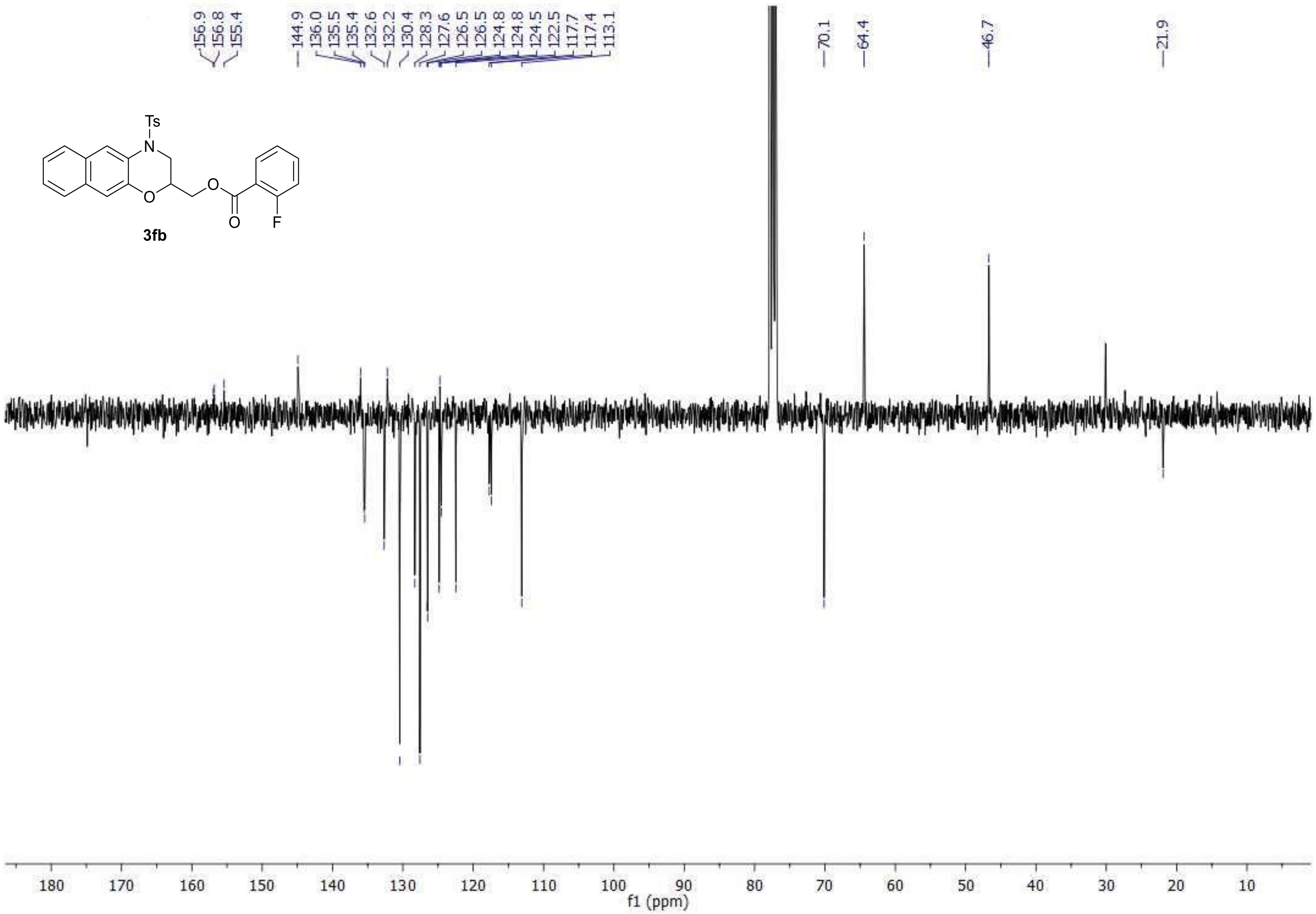

^{1}H NMR (300 MHz, $CDCl_3$) of **4aa**

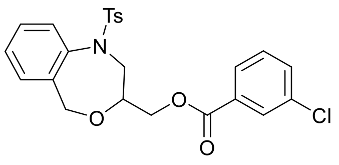

4aa

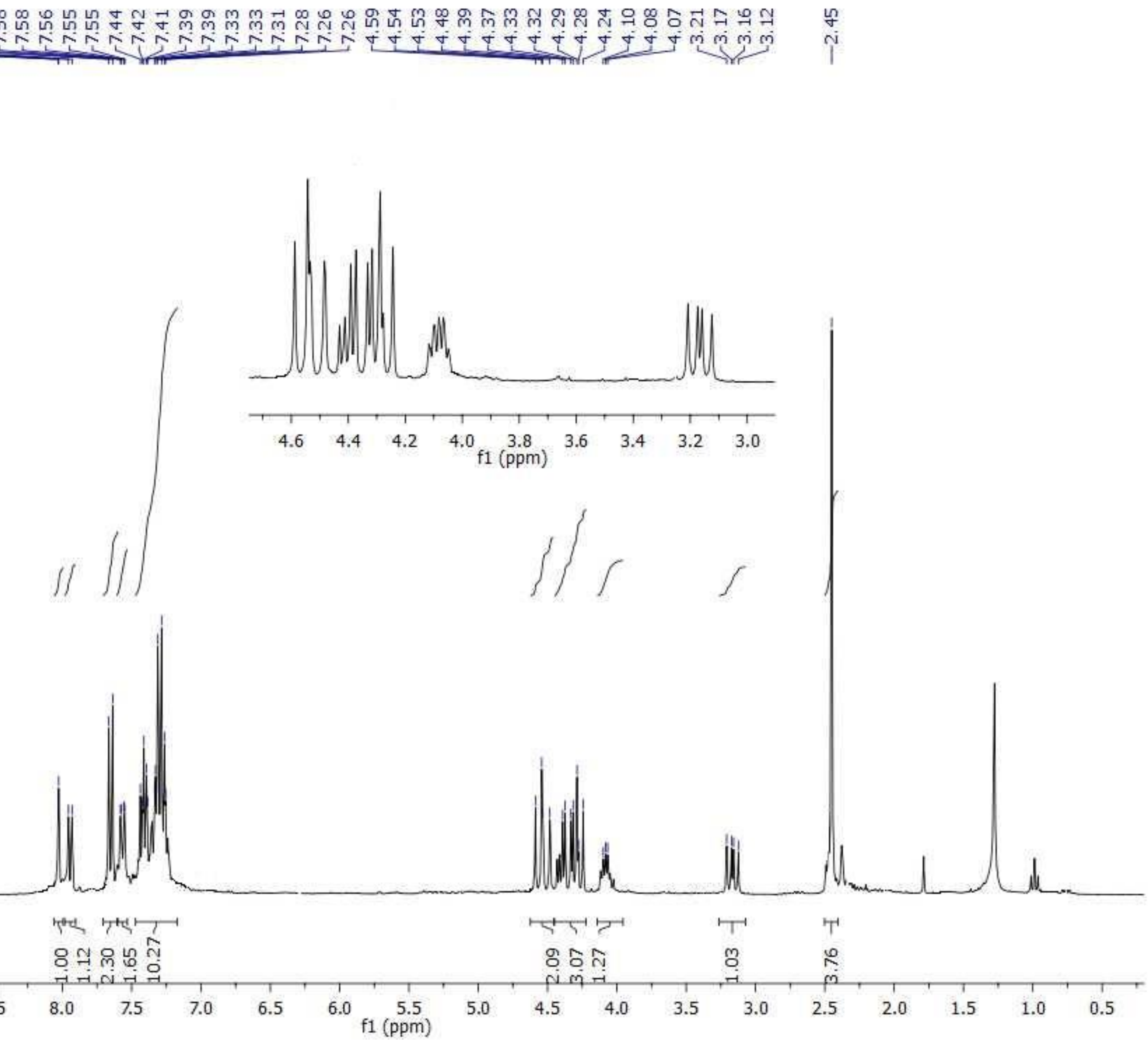

^{13}C NMR (75 MHz, $CDCl_3$) of **4aa**

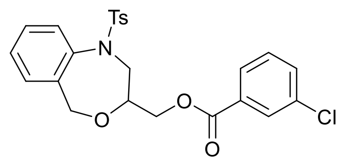

4aa

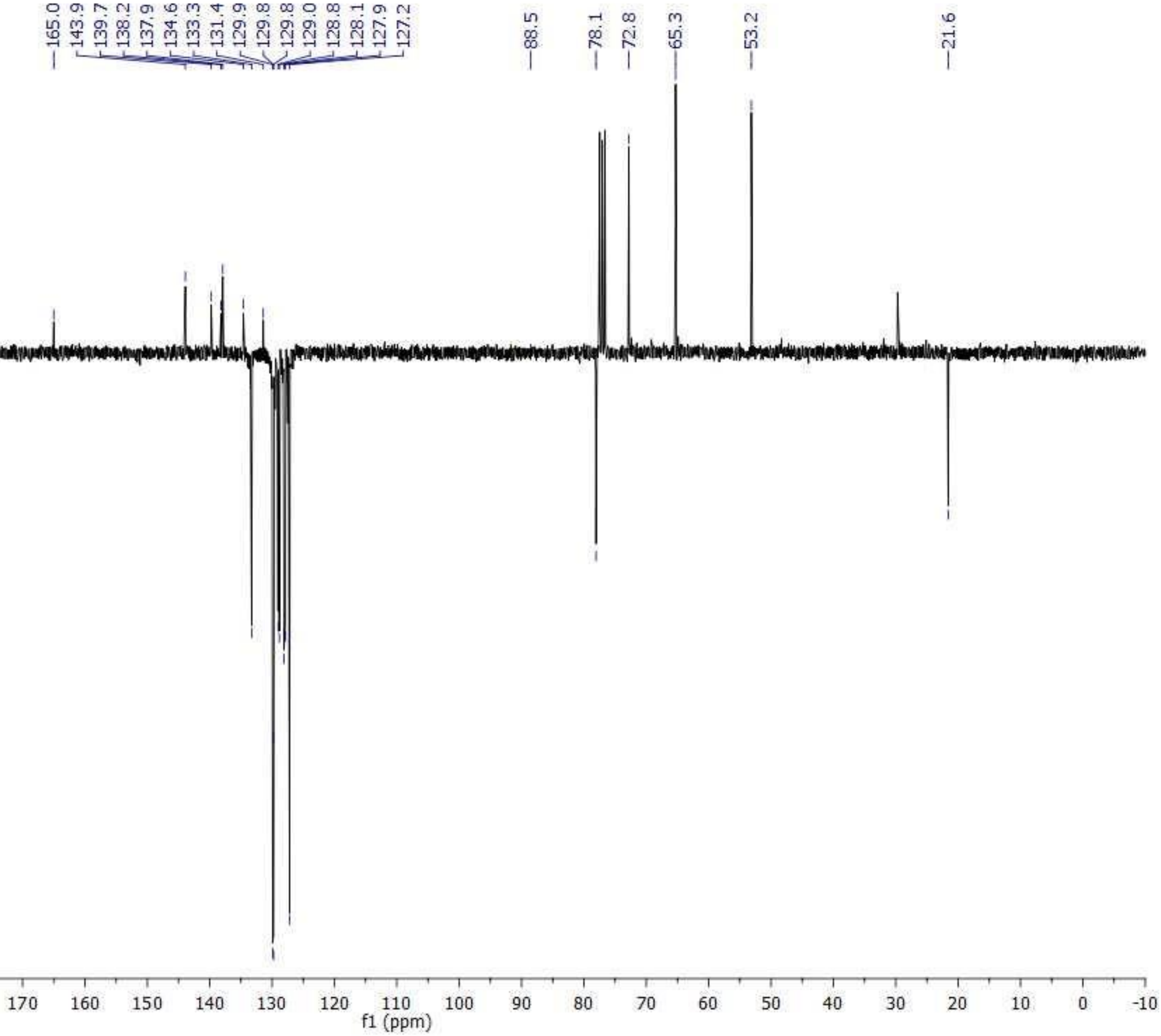

^1H NMR (300 MHz, $CDCl_3$) of **1i**

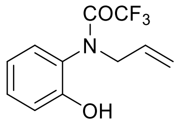

1i

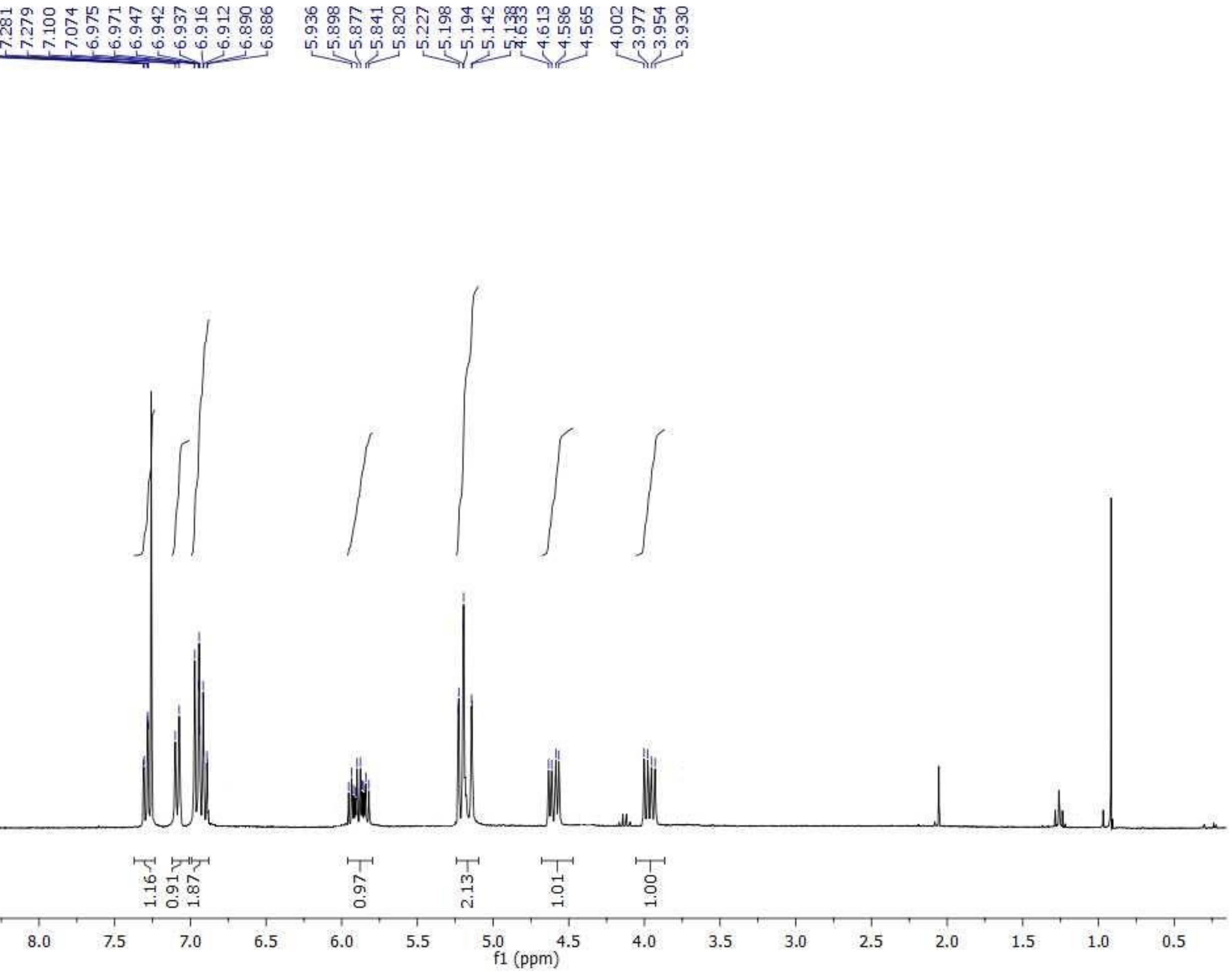

^{13}C NMR (75 MHz, $CDCl_3$) of **1i**

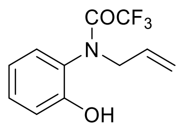

1i

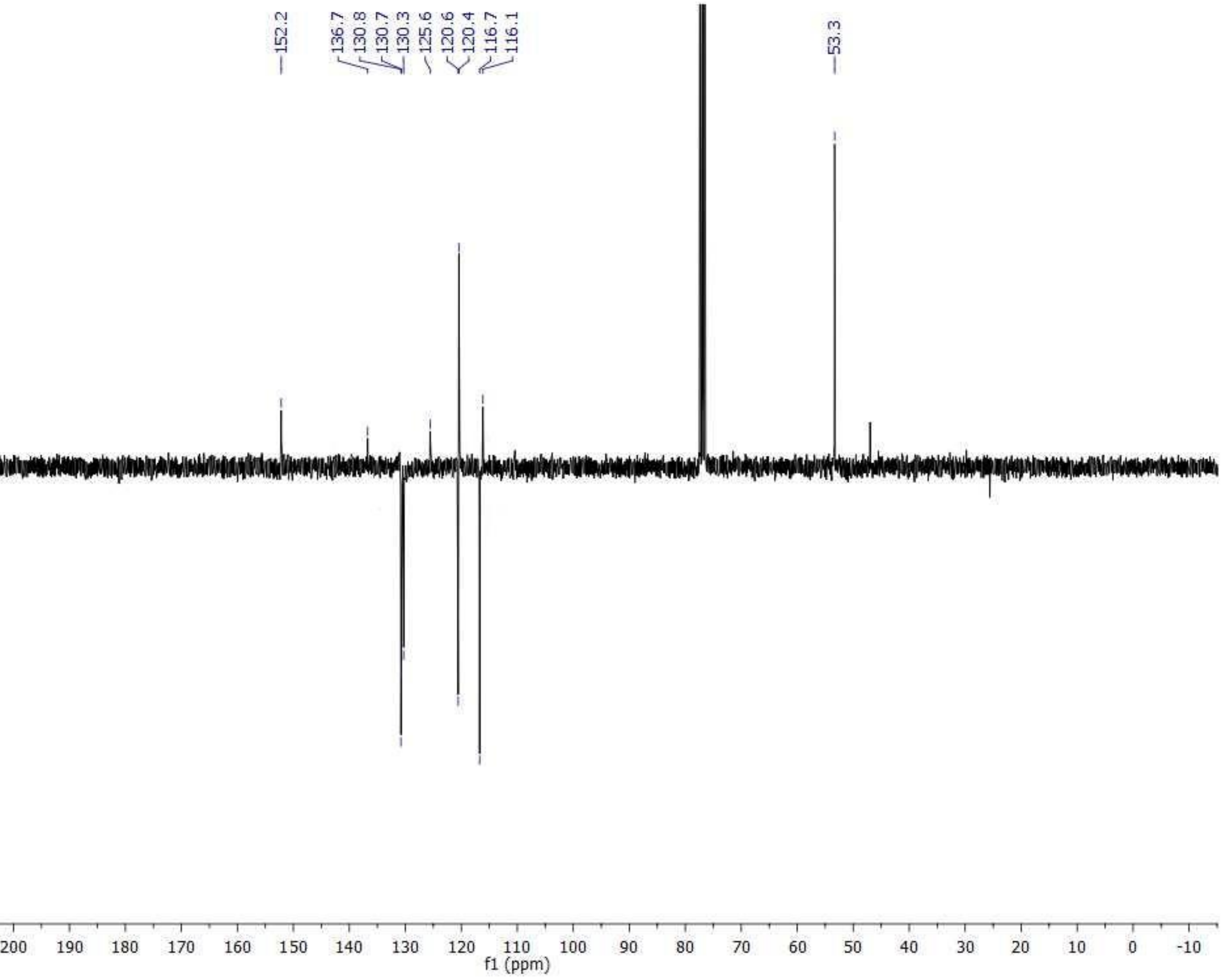

^{1}H NMR (300 MHz, $CDCl_3$) of **1j**

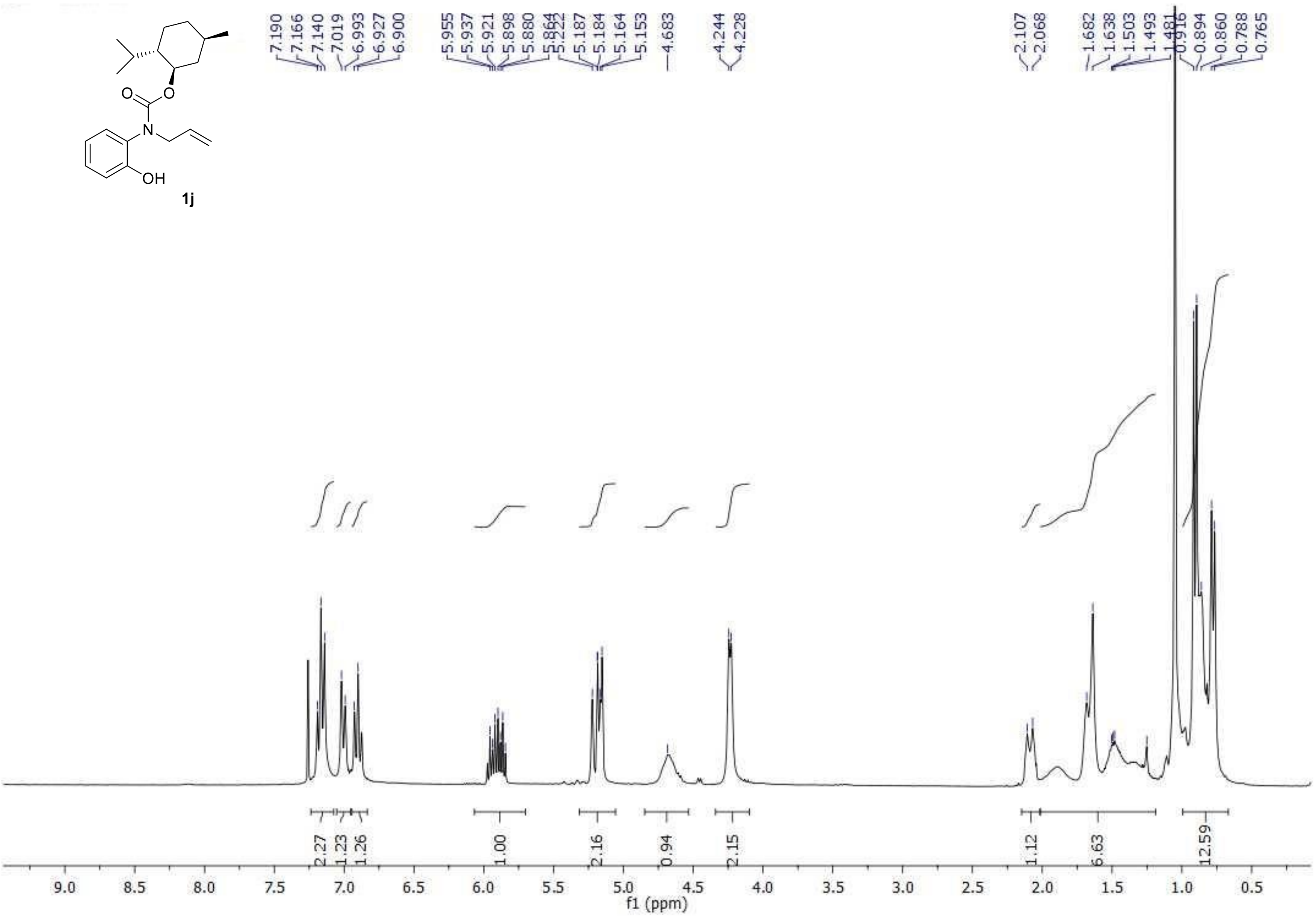

^{13}C NMR (75 MHz, $CDCl_3$) of **1j**

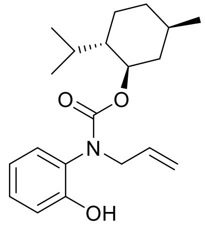

1j

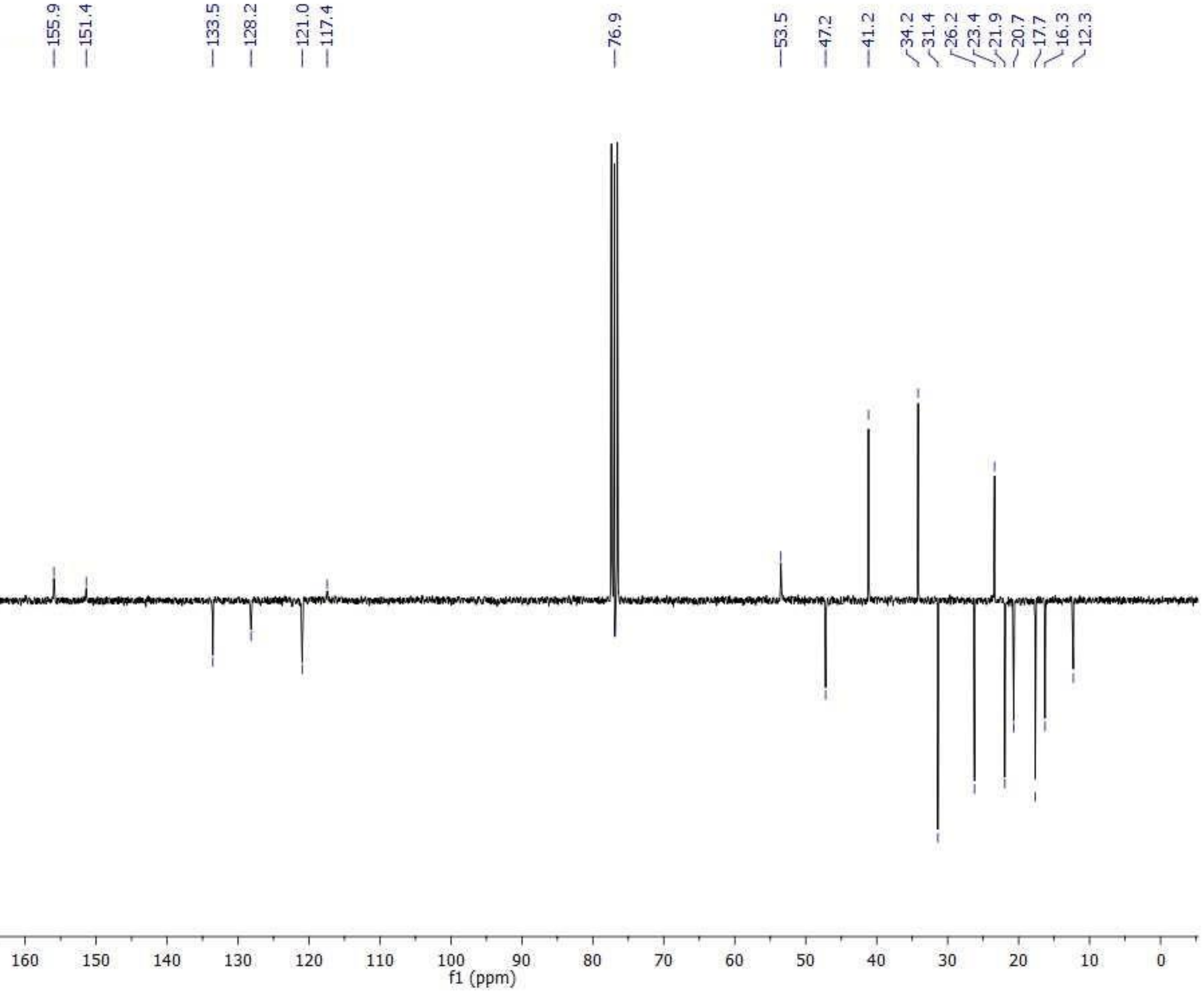

^1H NMR (500 MHz, $CDCl_3$) of **5aa**

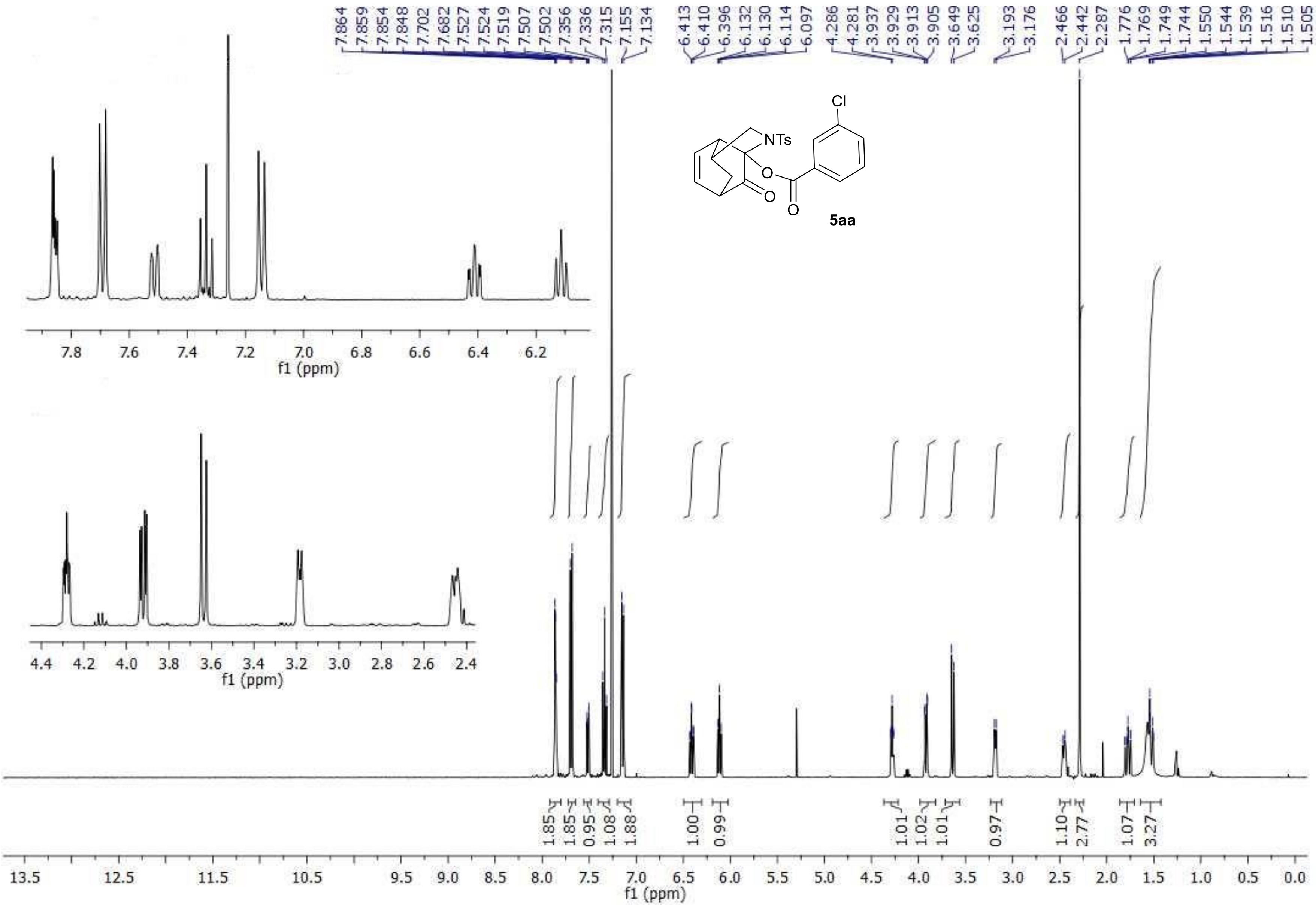

H-^{1}H COSY NMR (500 MHz, $CDCl_3$) of **5aa**

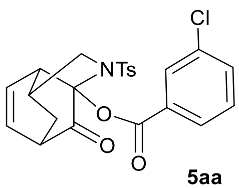

5aa

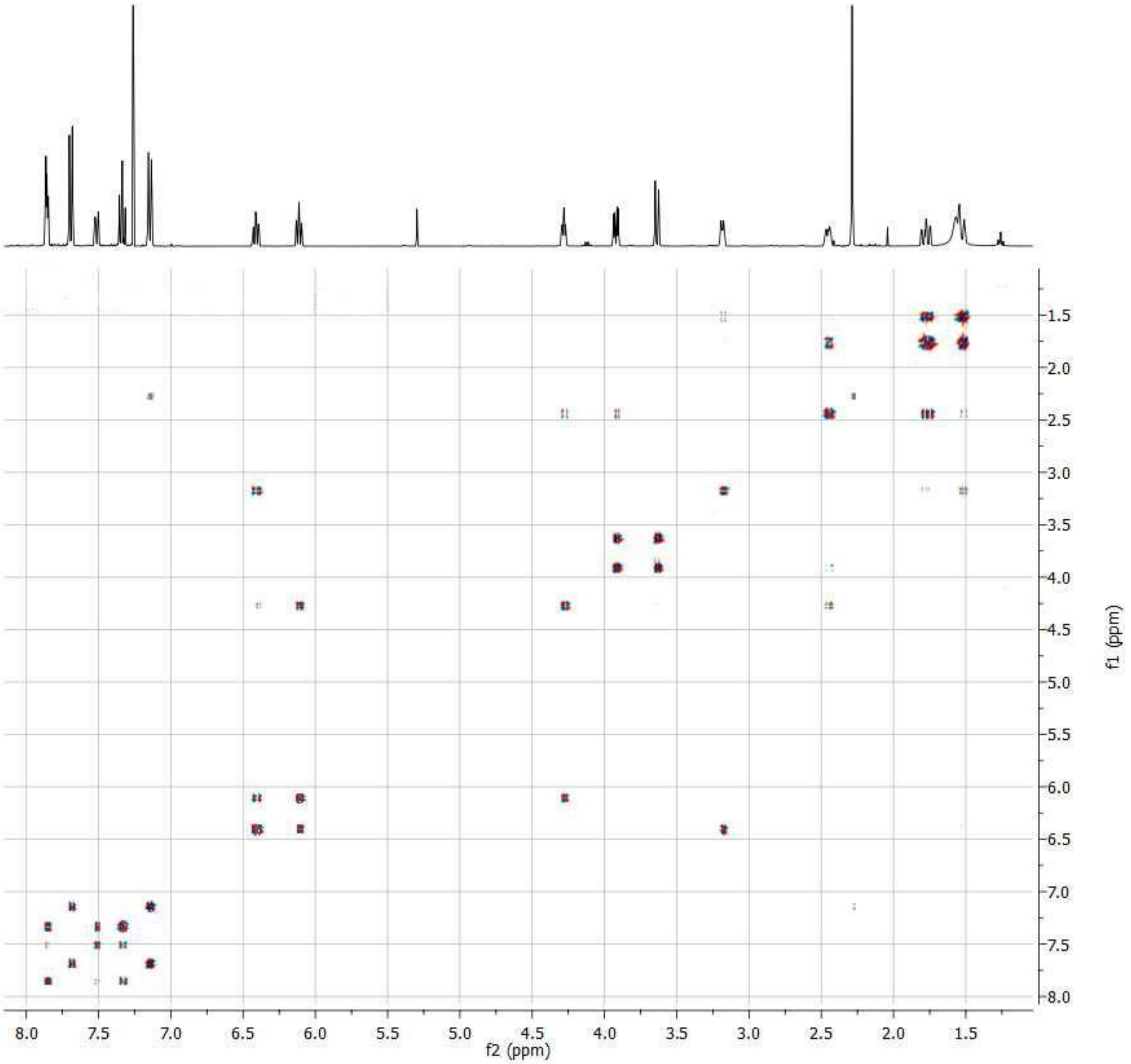

^{13}C NMR (126 MHz, $CDCl_3$) of **5aa**

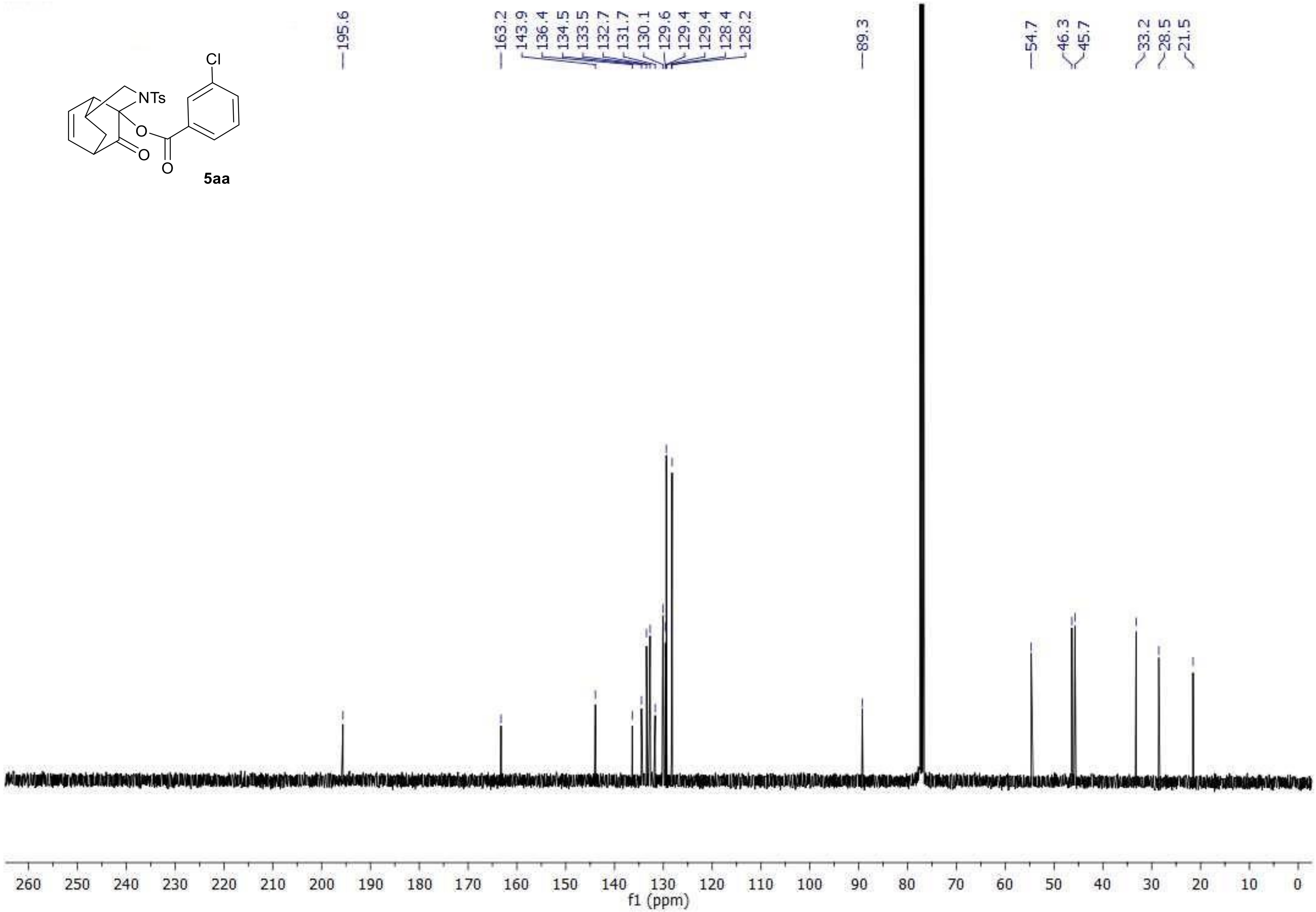

^{1}H NMR (300 MHz, $CDCl_3$) of **5ad**

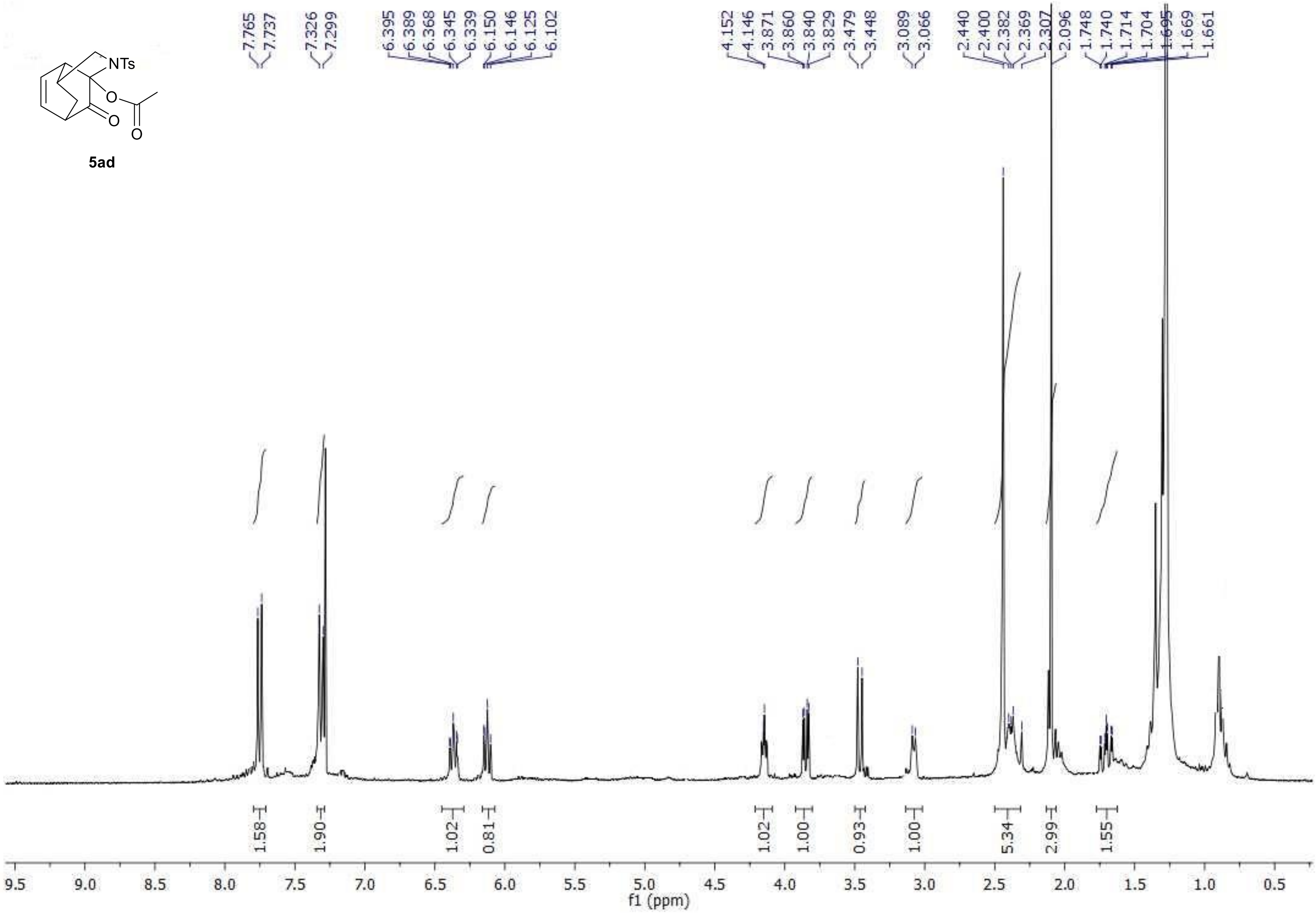

^{13}C NMR (75 MHz, $CDCl_3$) of **5ad**

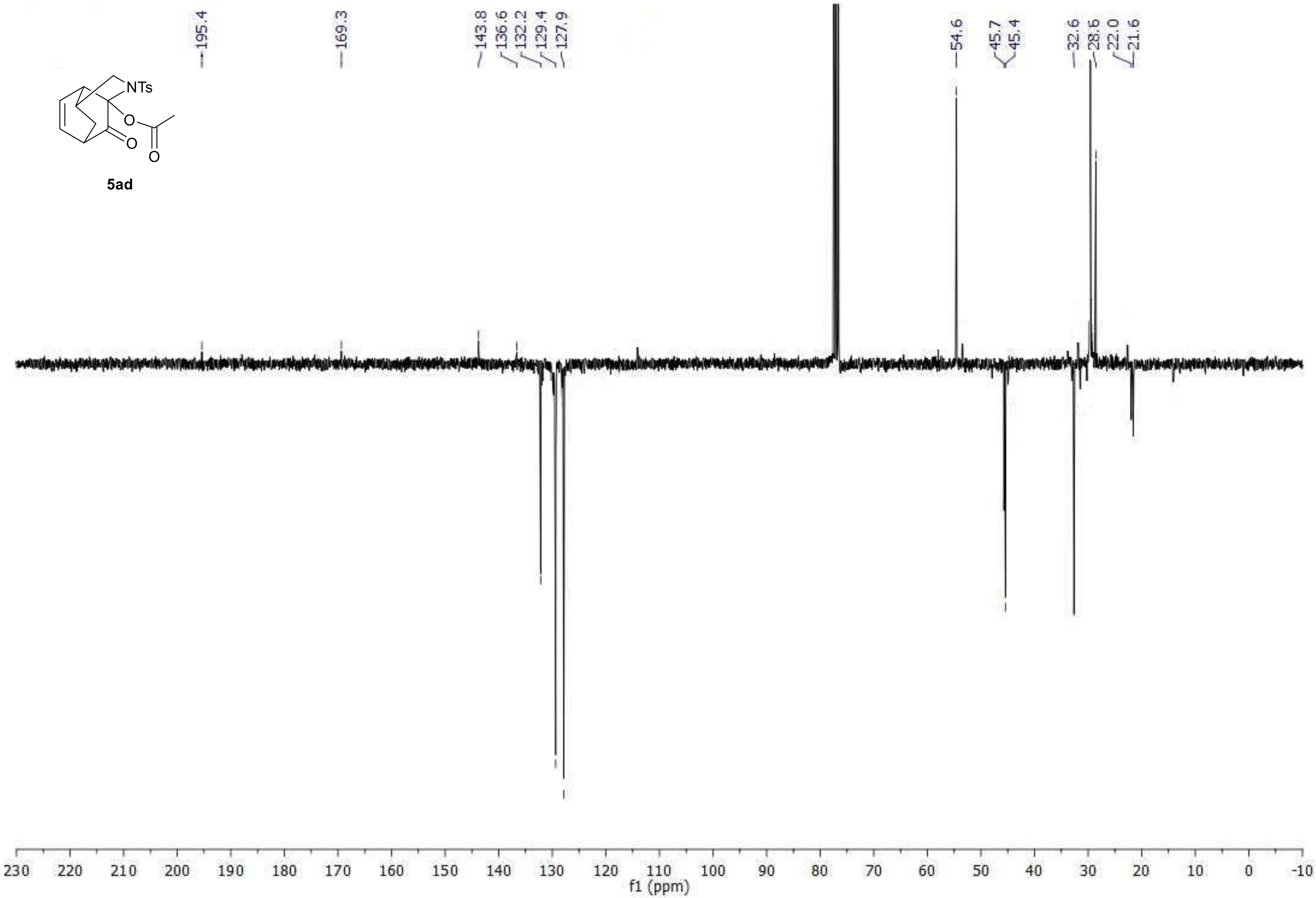

^{1}H NMR (300 MHz, $CDCl_3$) of **5ab**

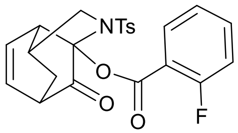

5ab

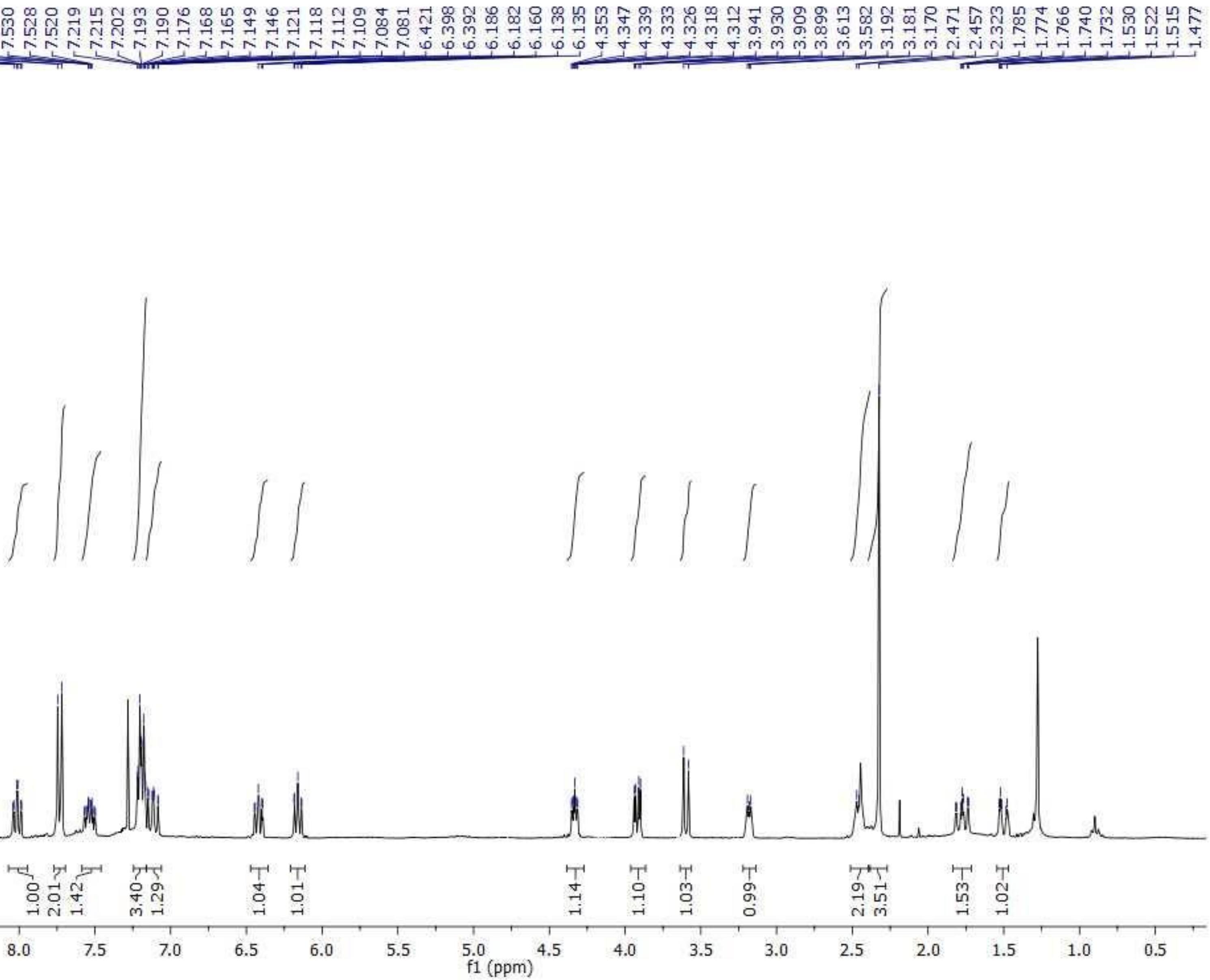

^{13}C NMR (75 MHz, CDCl$_3$) of **5ab**

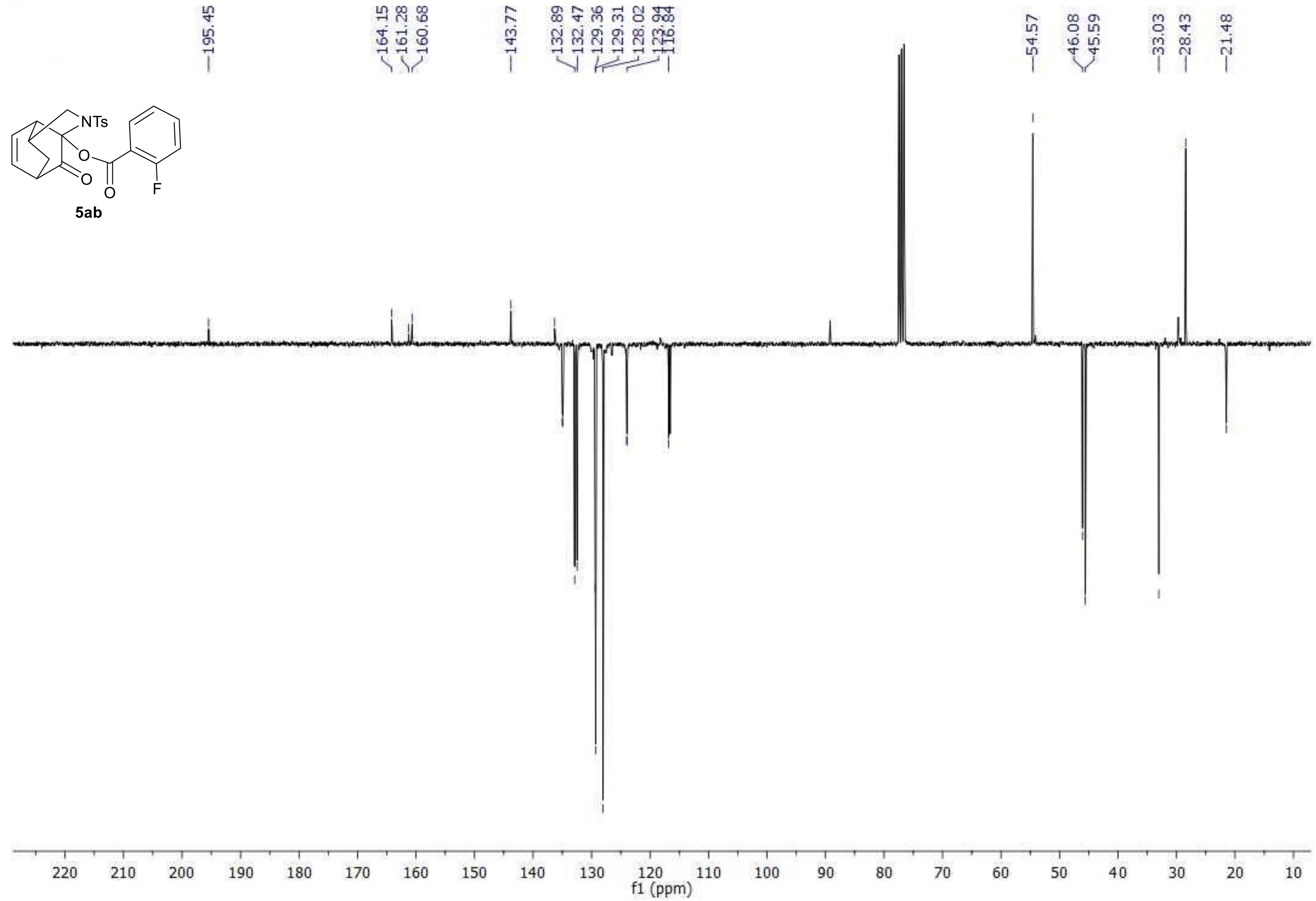

^{1}H NMR (300 MHz, $CDCl_3$) of **5ac**

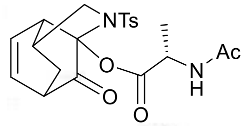

5ac

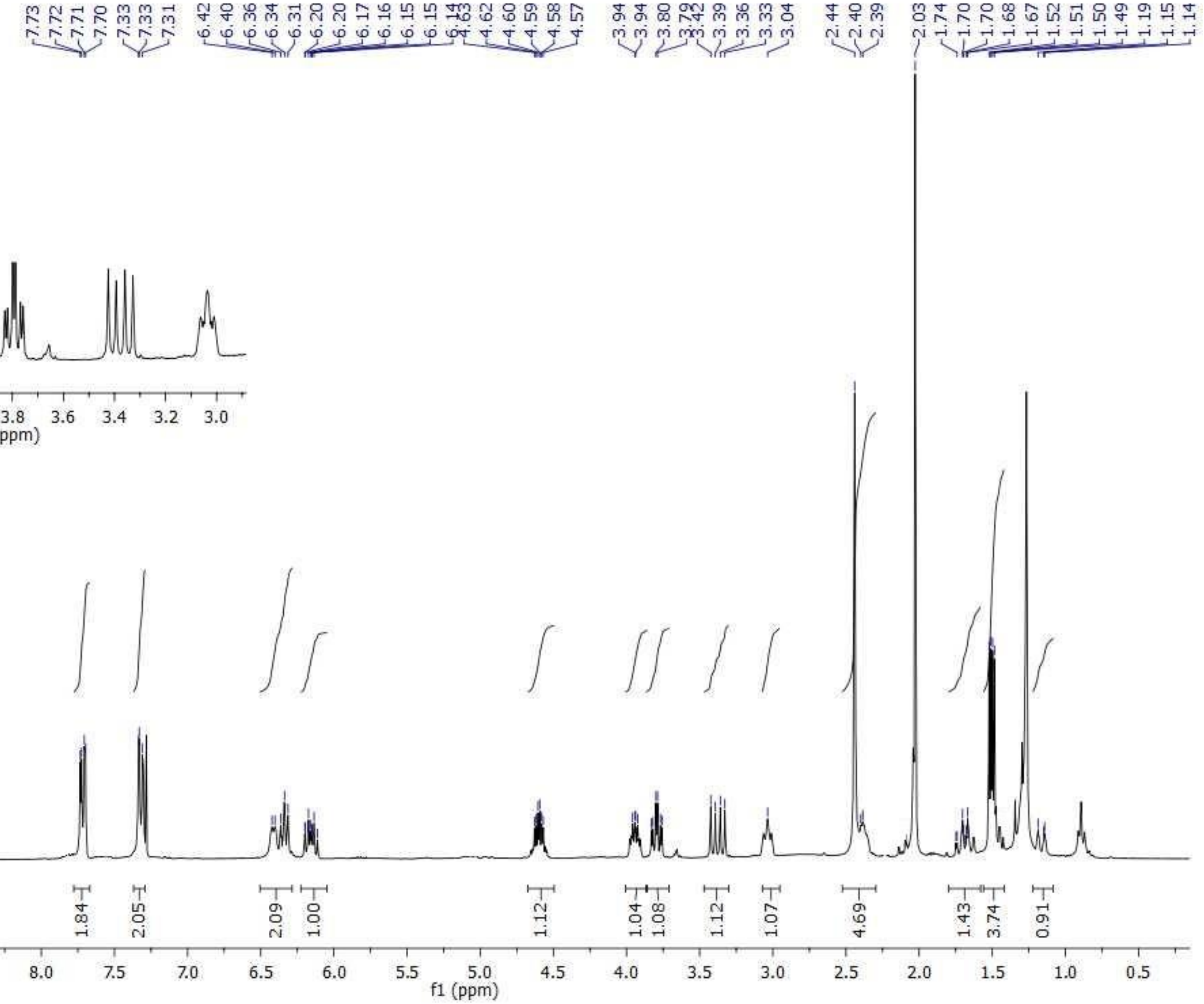

^{13}C NMR (75 MHz, $CDCl_3$) of **5ac**

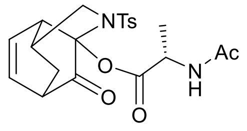

5ac

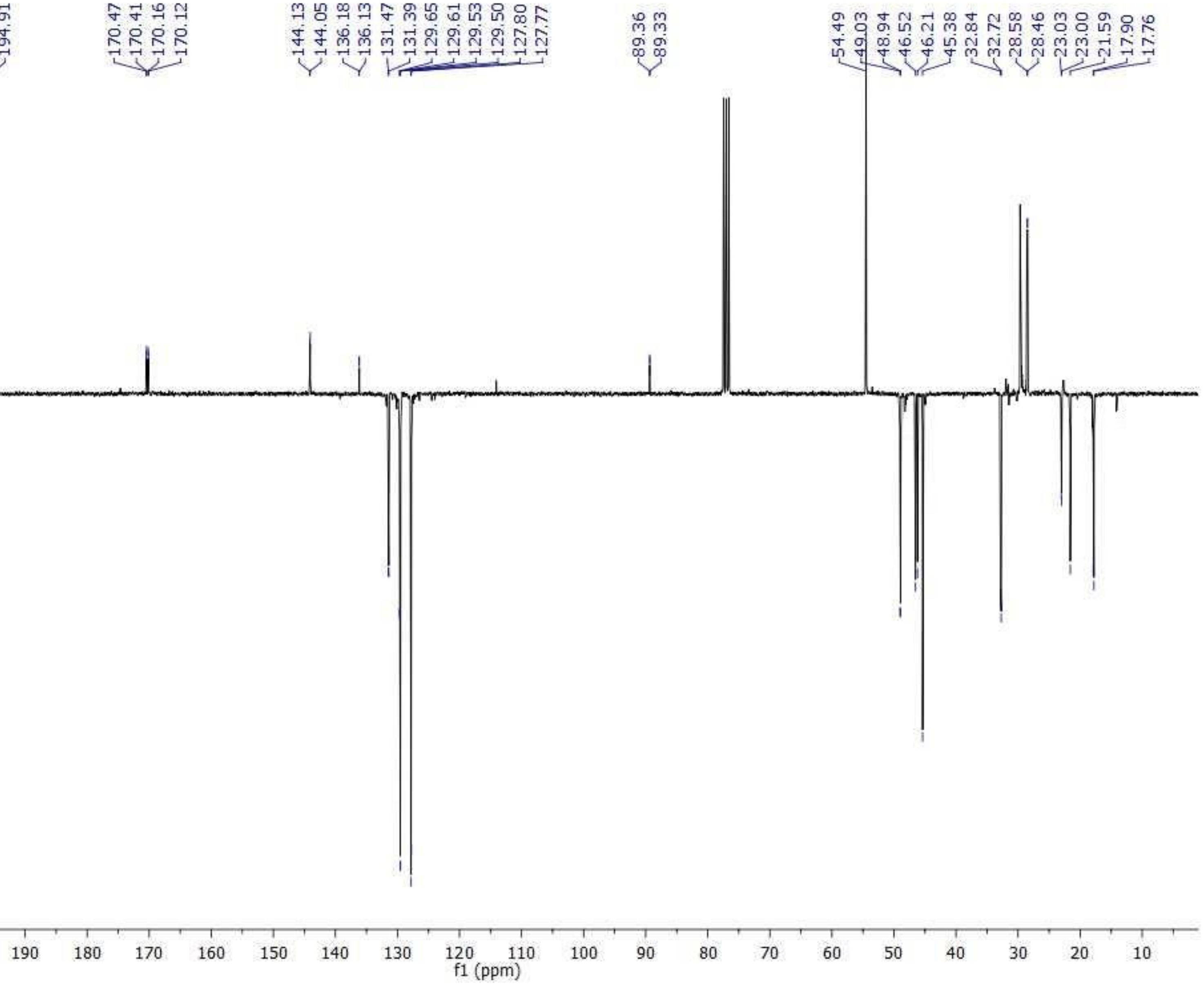

H-^{1}H COSY NMR (300 MHz, $CDCl_3$) of **5ac**

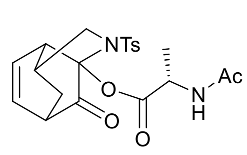

5ac

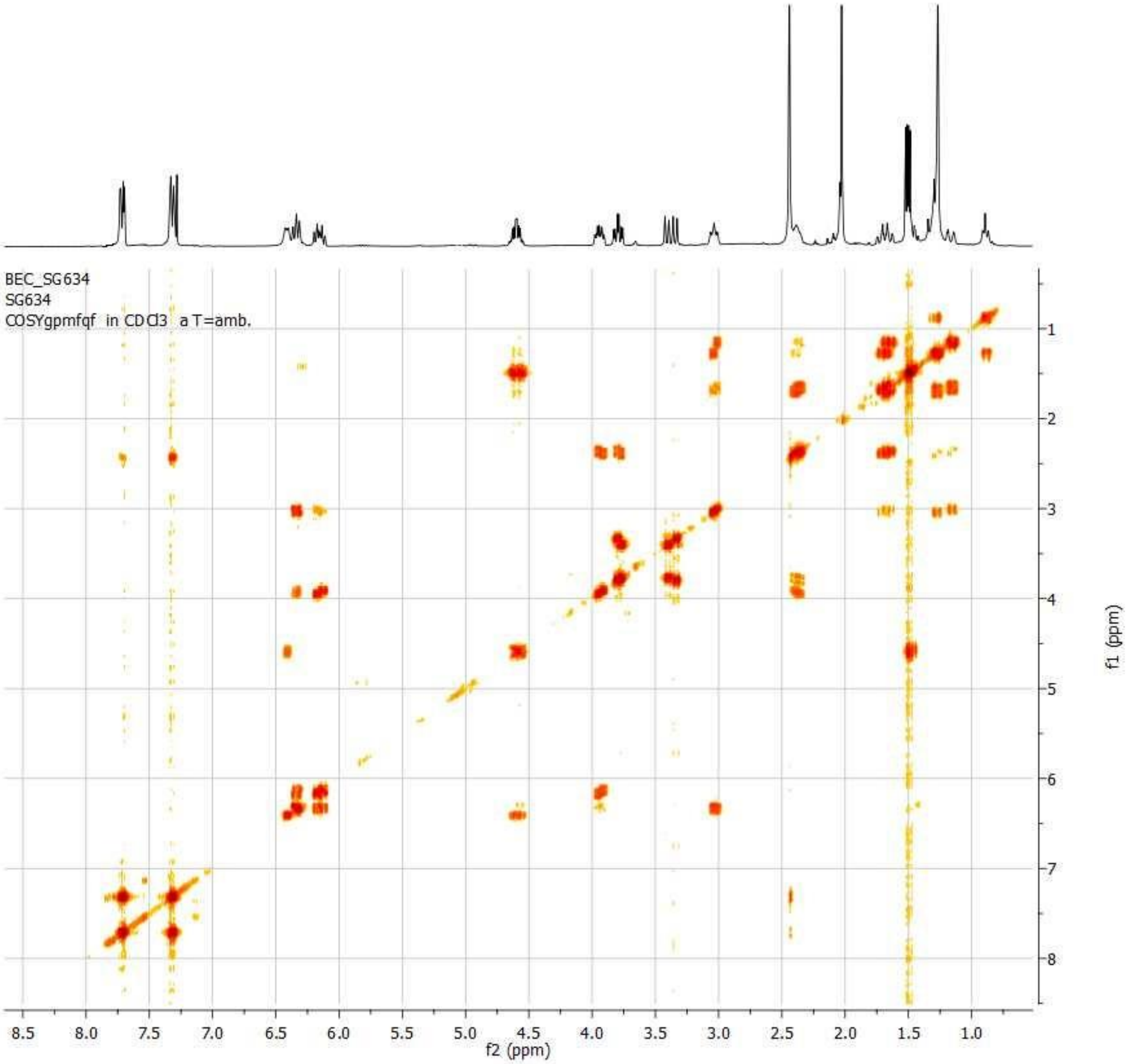

^{1}H NMR (300 MHz, $CDCl_3$) of **5ae**

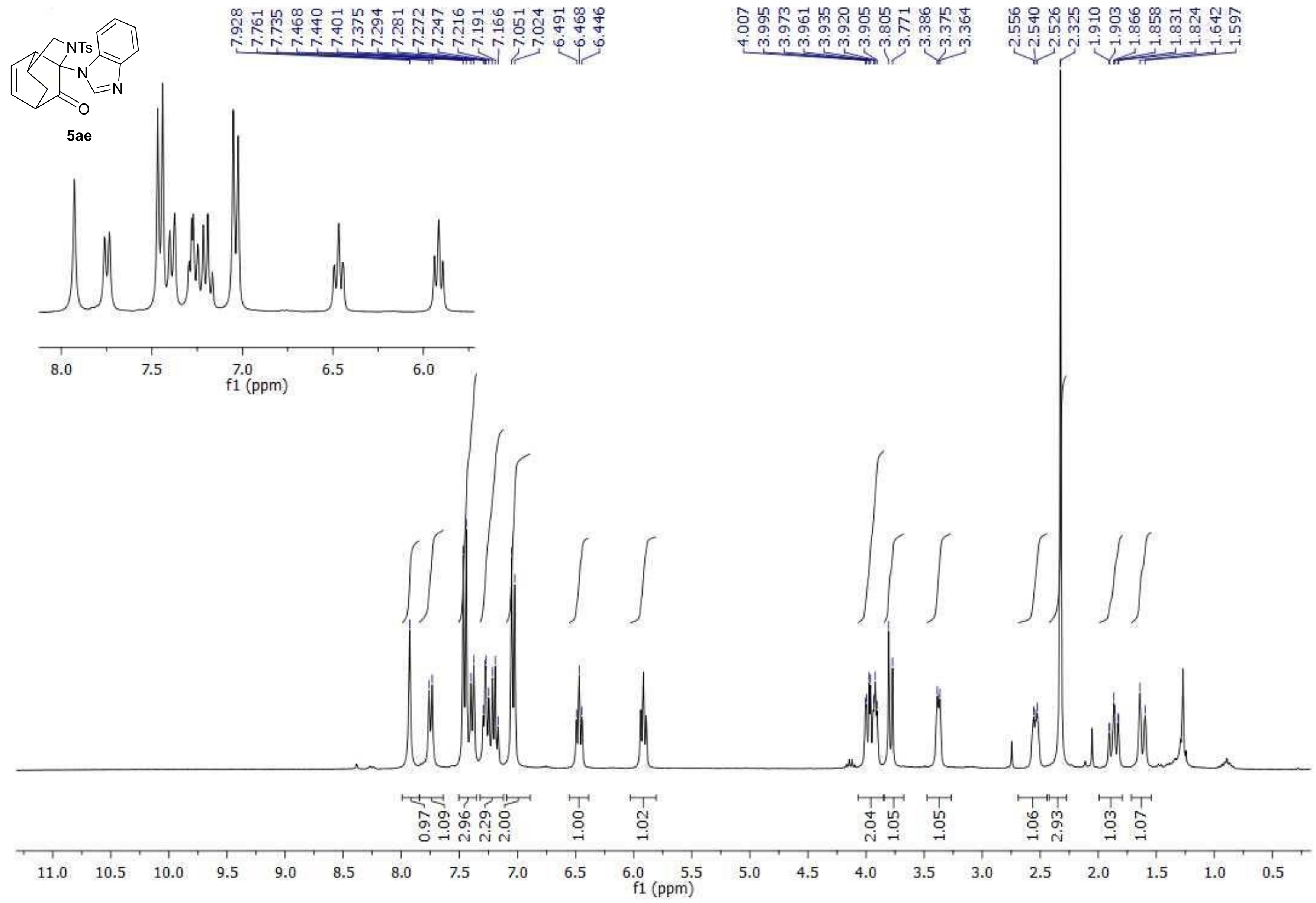

H-^{1}H COSY NMR (300 MHz, $CDCl_3$) of **5ae**

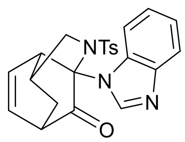

5ae

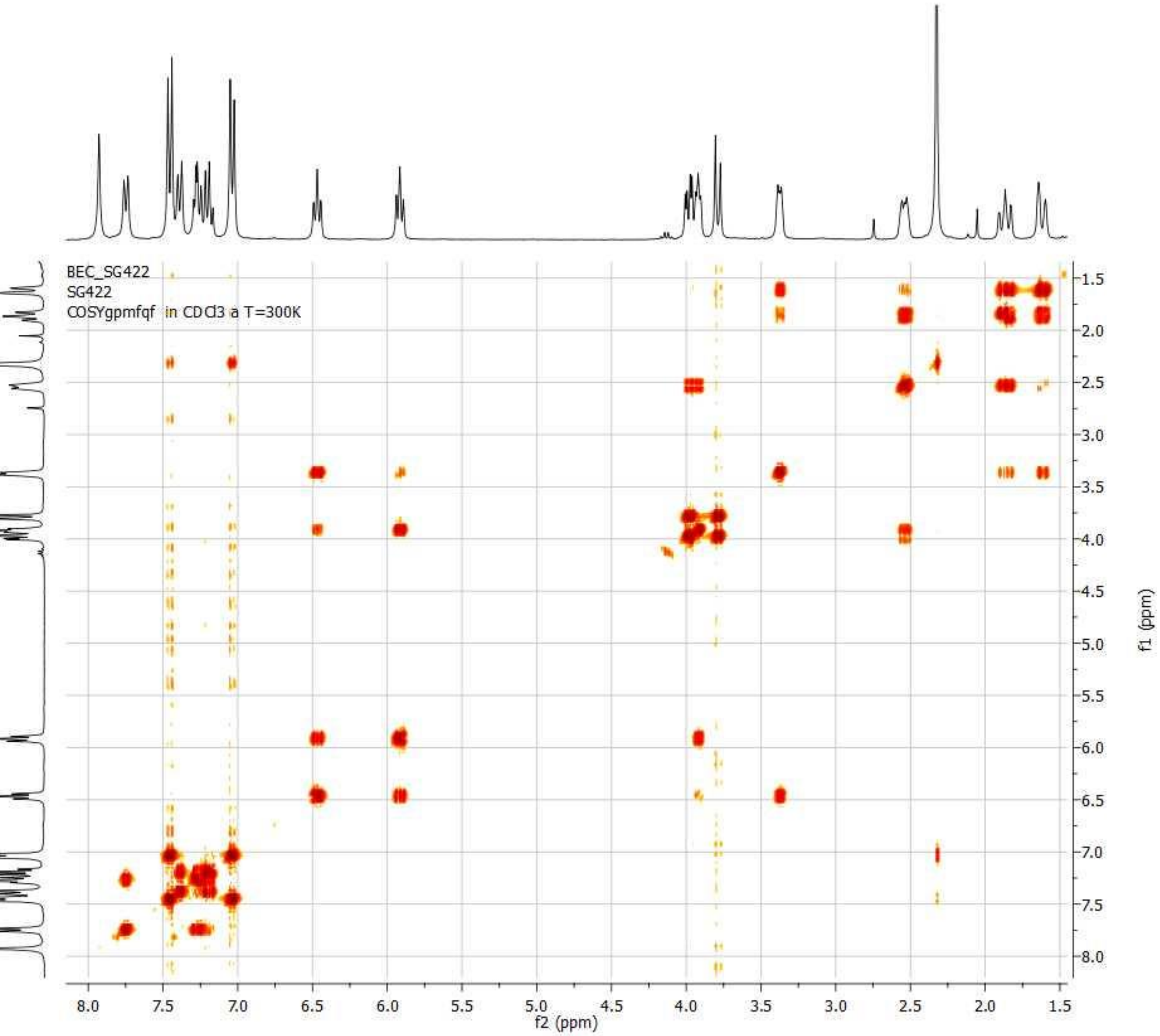

^{13}C NMR (75 MHz, $CDCl_3$) of **5ae**

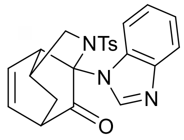

5ae

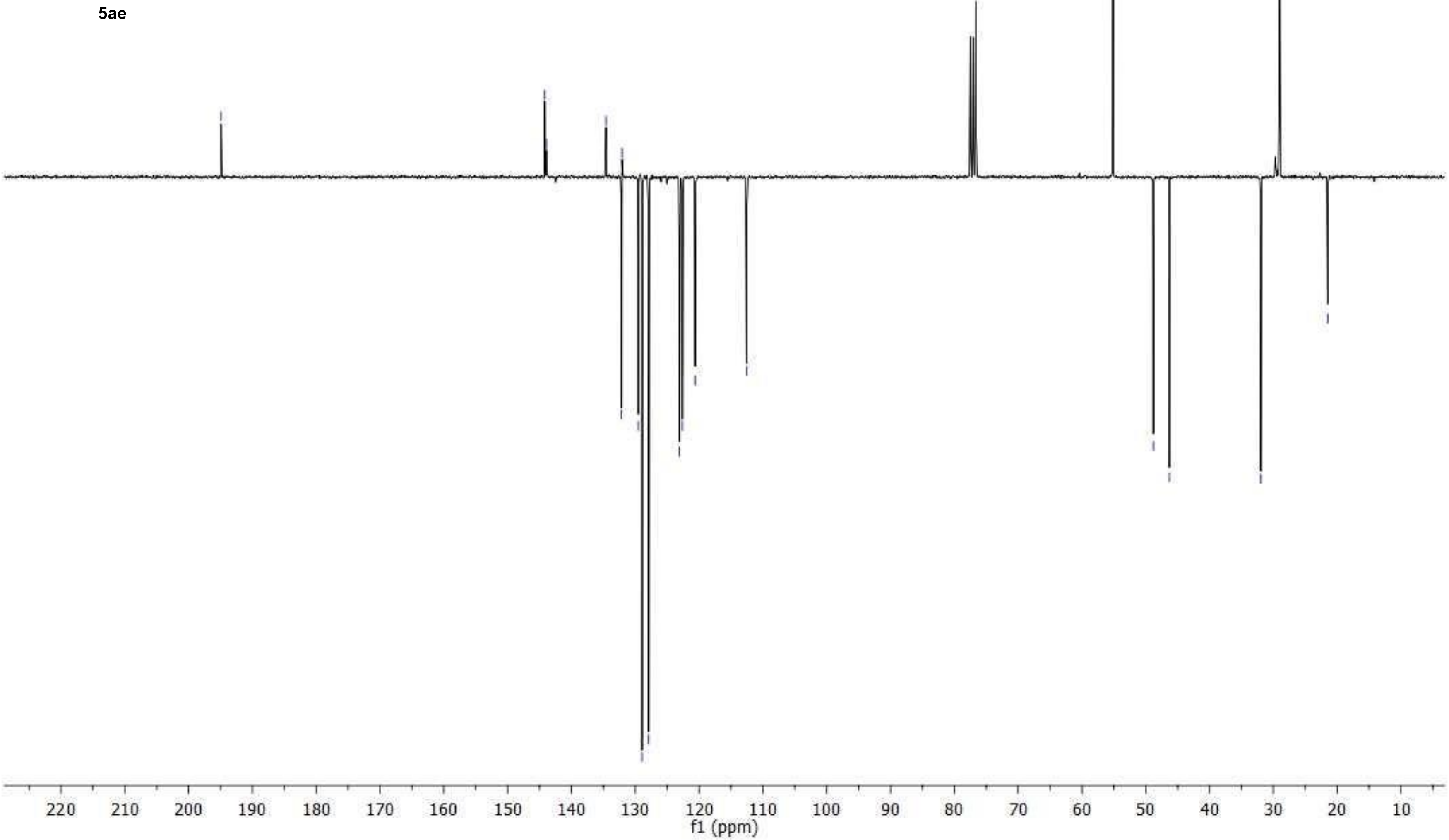

^{1}H NMR (500 MHz, CDCl$_3$) of **5ja**

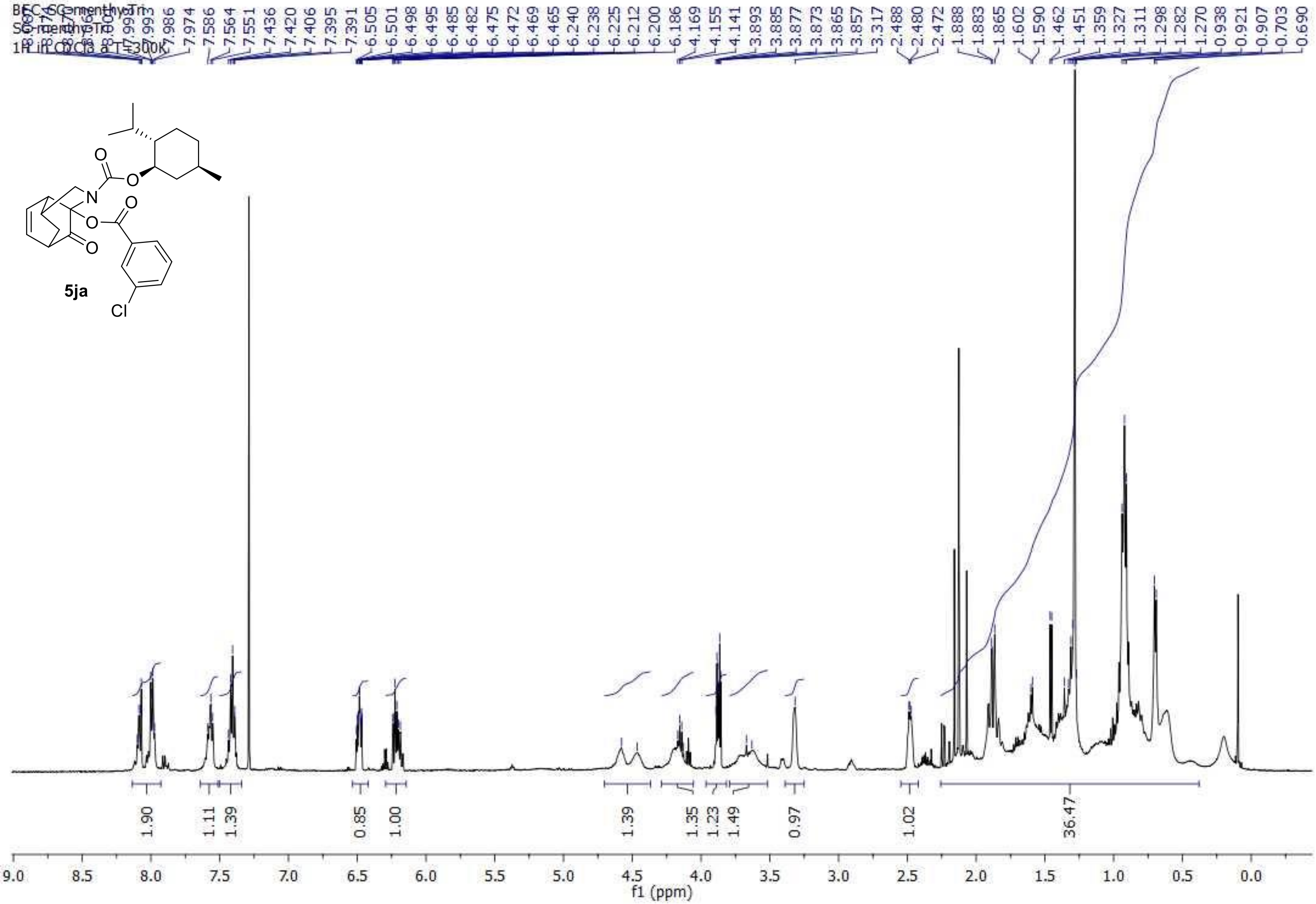

^{13}C NMR (126 MHz, $CDCl_3$) of **5ja**

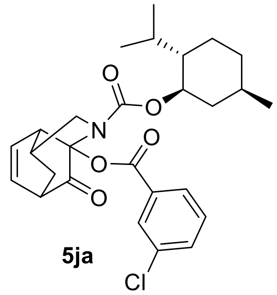

5ja

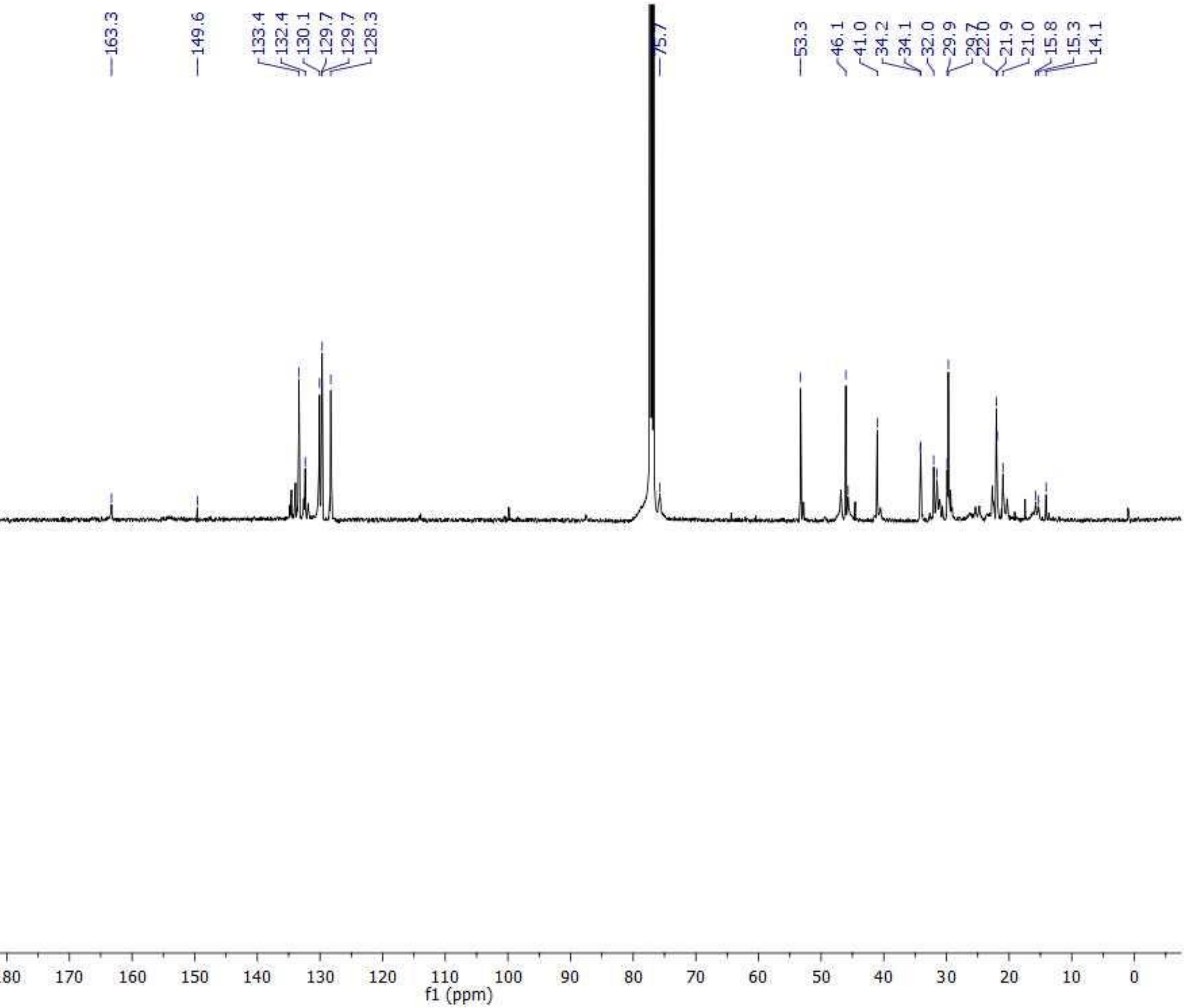

^1H NMR (300 MHz, $CDCl_3$) of **5da**

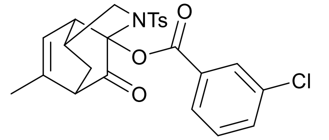

5da

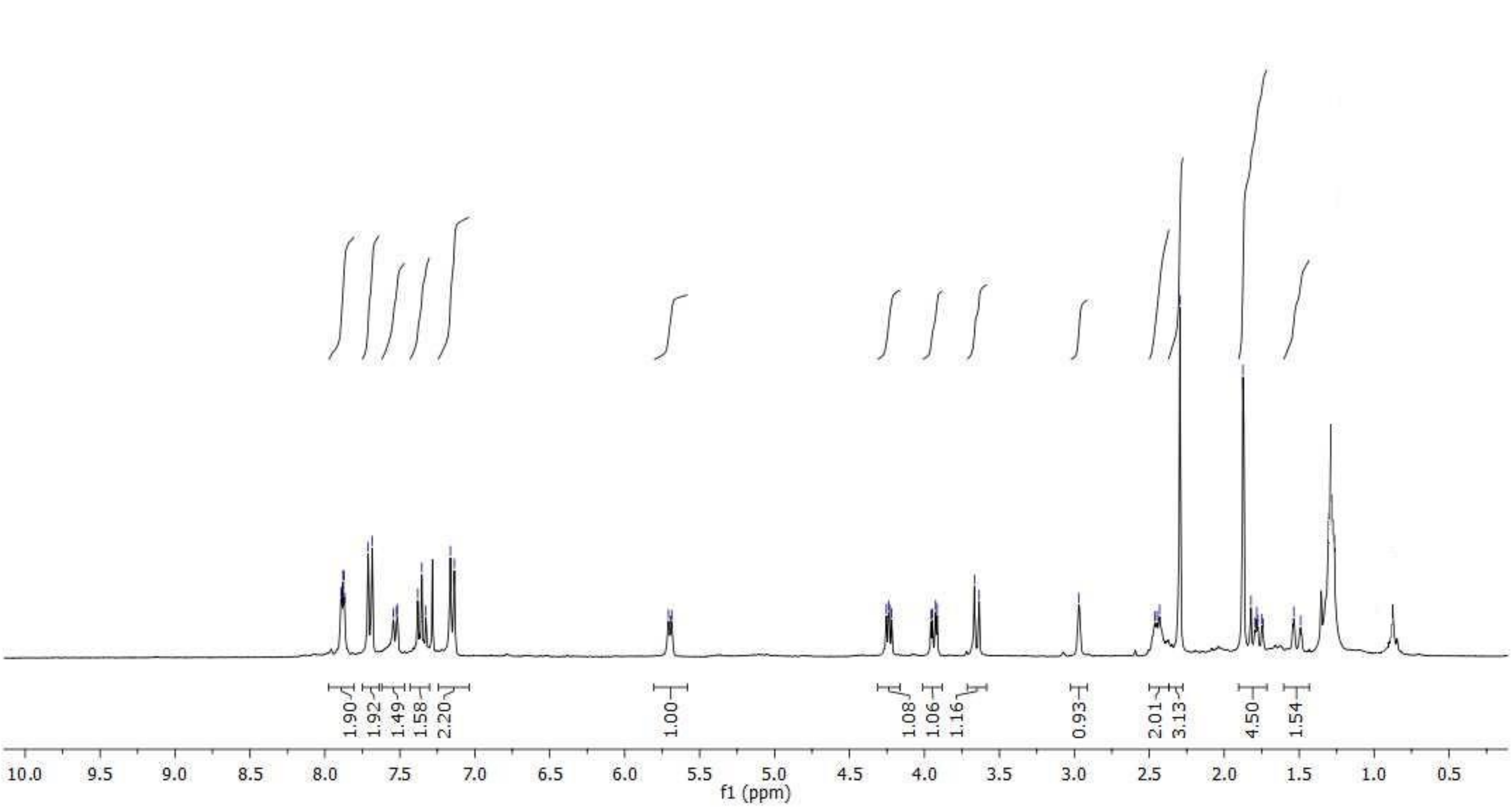

^{13}C NMR (75 MHz, $CDCl_3$) of **5da**

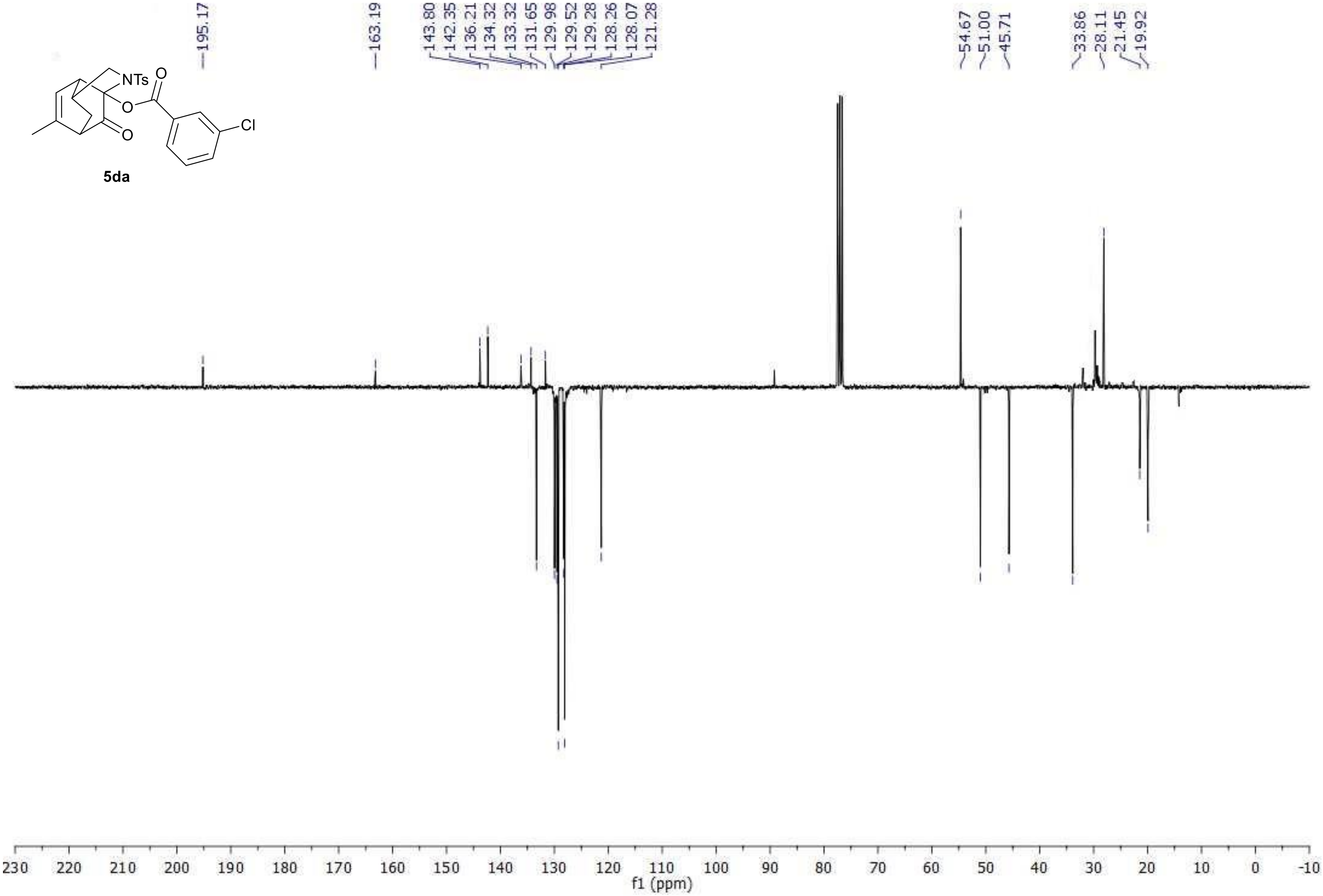

^{1}H NMR (300 MHz, $CDCl_3$) of **5ga**

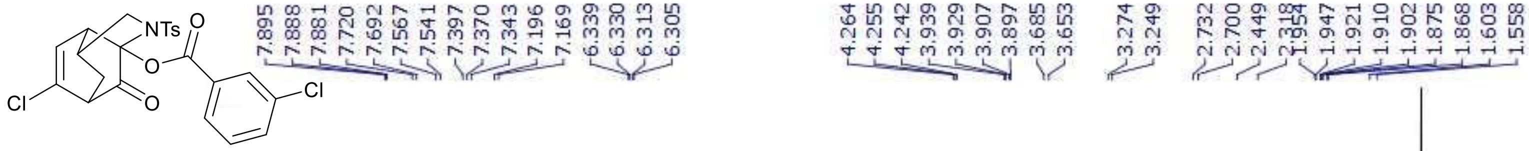

5ga

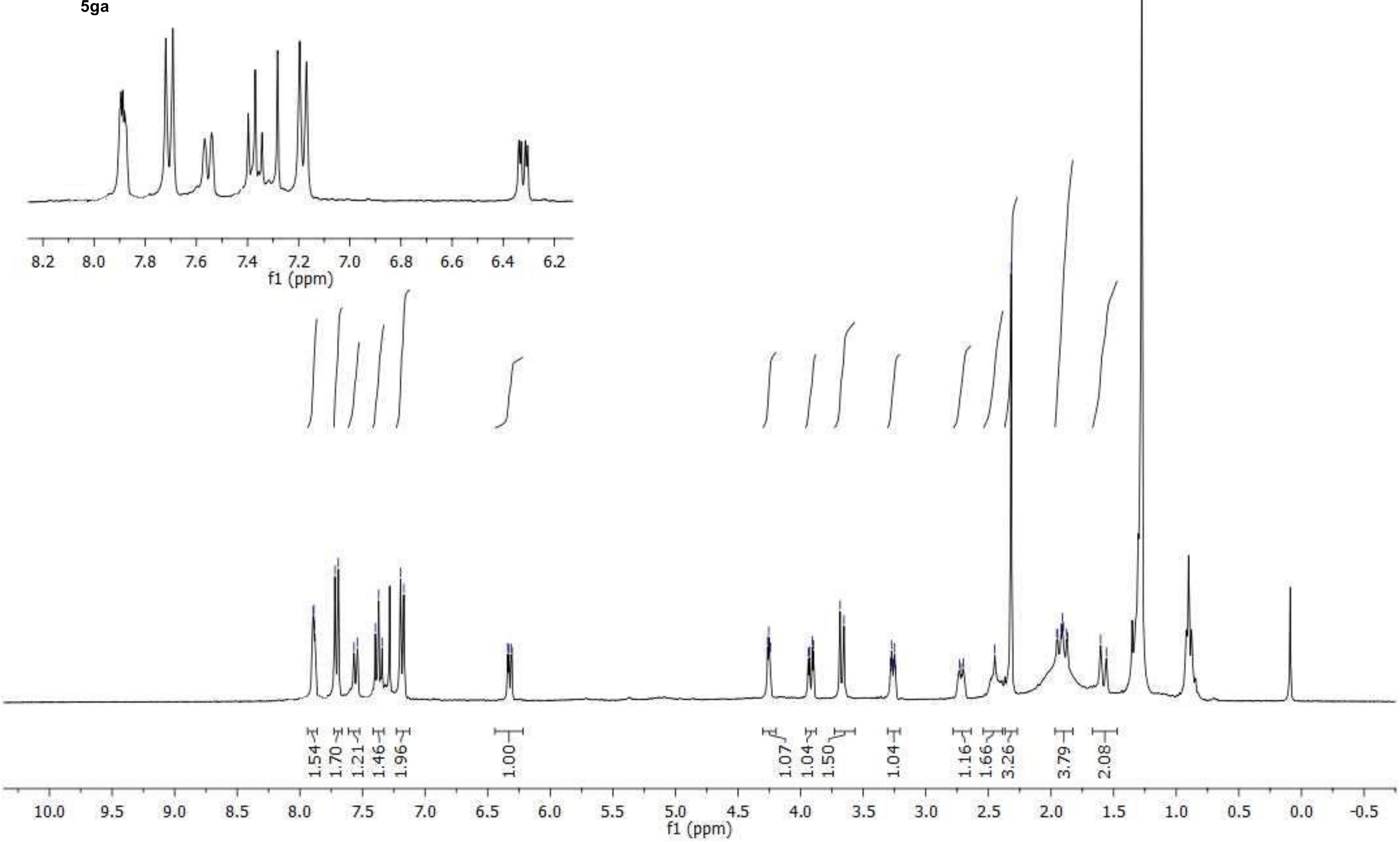

^{13}C NMR (75 MHz, $CDCl_3$) of **5ga**

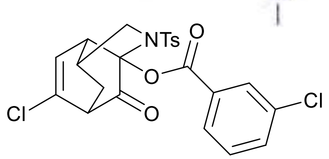

5ga

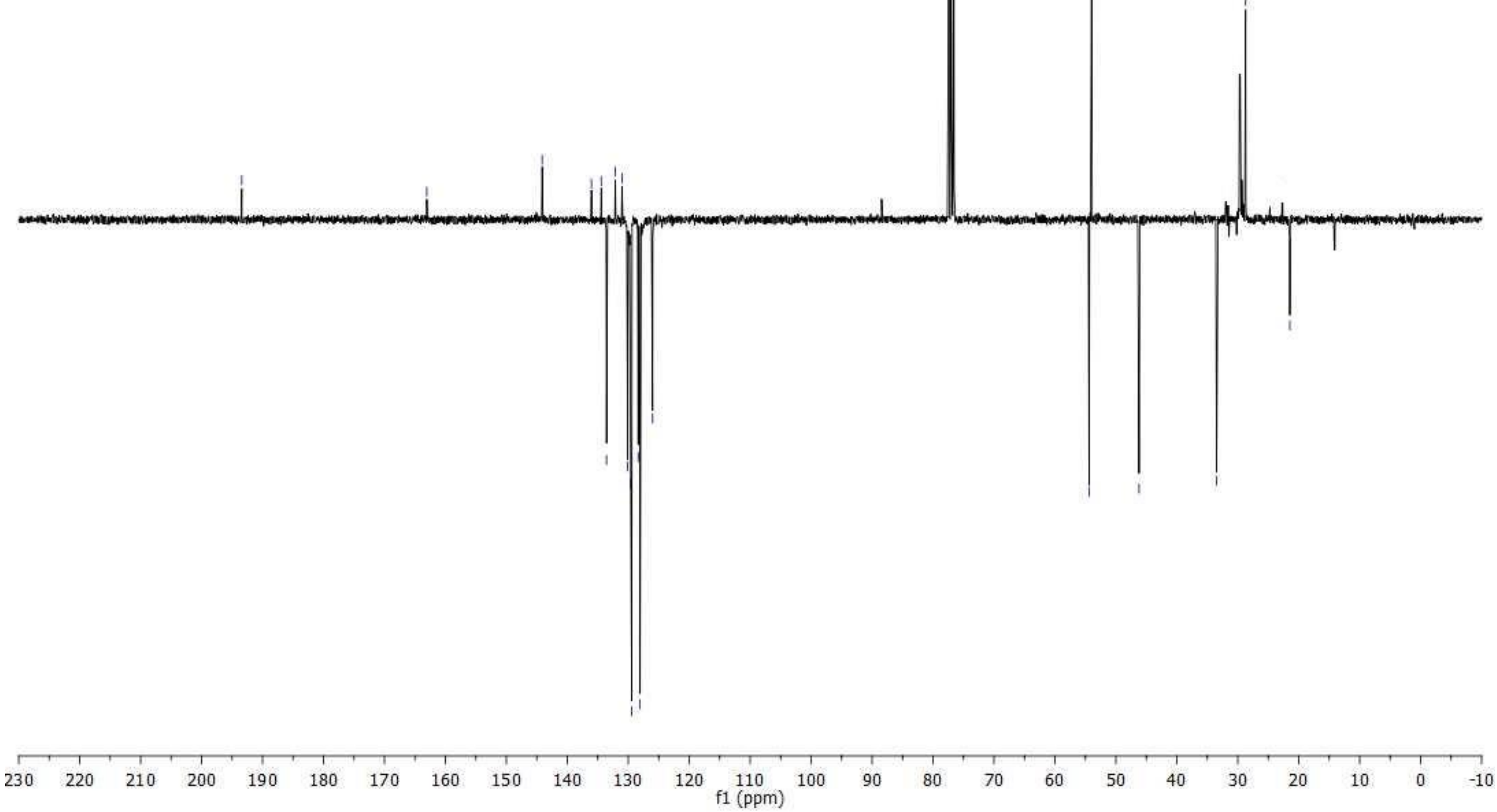

Supplement: Supplementary file 1 — ol1c02539_si_001.pdf [file ol1c02539_si_001.pdf]
